# *MAF* amplification licenses ERα through epigenetic remodelling to drive breast cancer metastasis

**DOI:** 10.1038/s41556-023-01281-y

**Published:** 2023-11-09

**Authors:** Alicia Llorente, María Teresa Blasco, Irene Espuny, Marc Guiu, Cecilia Ballaré, Enrique Blanco, Adrià Caballé, Anna Bellmunt, Fernando Salvador, Andrea Morales, Marc Nuñez, Guillem Loren, Francesca Imbastari, Marta Fidalgo, Cristina Figueras-Puig, Patrizia Gibler, Mariona Graupera, Freddy Monteiro, Antoni Riera, Ingunn Holen, Alexandra Avgustinova, Luciano Di Croce, Roger R. Gomis

**Affiliations:** 1 Cancer Science Program, Institute for Research in Biomedicine (IRB Barcelona), The Barcelona Institute of Science and Technology, Barcelona, Spain; 2 Centro de Investigación Biomédica en Red de Cáncer (CIBERONC), Madrid, Spain; 3 Centre for Genomic Regulation (CRG), Barcelona Institute of Science and Technology (BIST), Barcelona, Spain; 4 Biostatistics and Bioinformatics Unit, Institute for Research in Biomedicine (IRB Barcelona), The Barcelona Institute of Science and Technology, Barcelona, Spain; 5 Endothelial Pathobiology and Microenvironment Group, Josep Carreras Leukaemia Research Institute (IJC), Badalona, Barcelona, Spain; 6 Institució Catalana de Recerca i Estudis Avançats (ICREA), Barcelona, Spain; 7 Functional Genomics Core Facility, Institute for Research in Biomedicine (IRB Barcelona), The Barcelona Institute of Science and Technology, Barcelona, Spain; 8 Universitat de Barcelona, Barcelona, Spain; 9 Department of Oncology and Metabolism, University of Sheffield, Sheffield, UK; 10 Institut de Recerca Sant Joan de Déu, Esplugues de Llobregat, Spain; 11 Universitat Pompeu Fabra (UPF), Barcelona, Spain

**Keywords:** Breast cancer, Metastasis, Cancer epigenetics

## Abstract

*MAF* amplification increases the risk of breast cancer (BCa) metastasis through mechanisms that are still poorly understood yet have important clinical implications. Oestrogen-receptor-positive (ER^+^) BCa requires oestrogen for both growth and metastasis, albeit by ill-known mechanisms. Here we integrate proteomics, transcriptomics, epigenomics, chromatin accessibility and functional assays from human and syngeneic mouse BCa models to show that MAF directly interacts with oestrogen receptor alpha (ERα), thereby promoting a unique chromatin landscape that favours metastatic spread. We identify metastasis-promoting genes that are de novo licensed following oestrogen exposure in a MAF-dependent manner. The histone demethylase KDM1A is key to the epigenomic remodelling that facilitates the expression of the pro-metastatic MAF/oestrogen-driven gene expression program, and loss of KDM1A activity prevents this metastasis. We have thus determined that the molecular basis underlying MAF/oestrogen-mediated metastasis requires genetic, epigenetic and hormone signals from the systemic environment, which influence the ability of BCa cells to metastasize.

## Abstract (web summary)

Llorente, Blasco, Espuny and colleagues show that MAF regulates the genomic distribution of ERα and modulates the expression of metastasis genes via KDM1A, thereby driving metastatic spread in breast cancer.

## Main

Hormonal signalling can send systemic signals to different tissues and is often hijacked by tumours to support metastatic spread^1^. Breast cancer (BCa) provides a paradigm of systemic hormone-associated metastasis progression: both oestrogen (E2; 17-β-oestradiol) and progesterone signalling pathways are central to the proliferation and progression of BCa and are lethal in its metastatic stage. The E2-driven cancer cell proliferation in oestrogen-receptor-positive (ER^+^) BCa is frequently targeted clinically by endocrine therapy (for example, with tamoxifen), but with limited long-term success due to frequent intrinsic or acquired therapy resistance^2^. Importantly, some ER^+^ BCas are more metastatic than others, despite displaying the same oncogenic dependency. The determinants of this diversity of metastatic phenotypes, and whether and how the endocrine milieu plays a direct role in promoting the metastatic process, have yet to be ascertained.

Bone is the predominant metastatic site in hormone-dependent breast and prostate cancer^3^. Bone metastasis is supported by the interactions between cancer cells and stromal cells (mainly osteoblasts and osteoclasts), which collectively generate a so-called osteolytic vicious cycle^4,5^. Bone-modifying agents have been developed to disrupt this cycle and help manage skeletal-related events (for example, with bisphosphonates, which inhibit osteoclast activity)^3^. However, although bone metastases are comparatively manageable clinically, they frequently act as the prelude for subsequent, multi-organ metastatic spread and eventually patient demise^6^, perhaps due to metastasis-to-metastasis^7^ (metastases resulting from other metastases^8,9^).

**Fig. 1:**
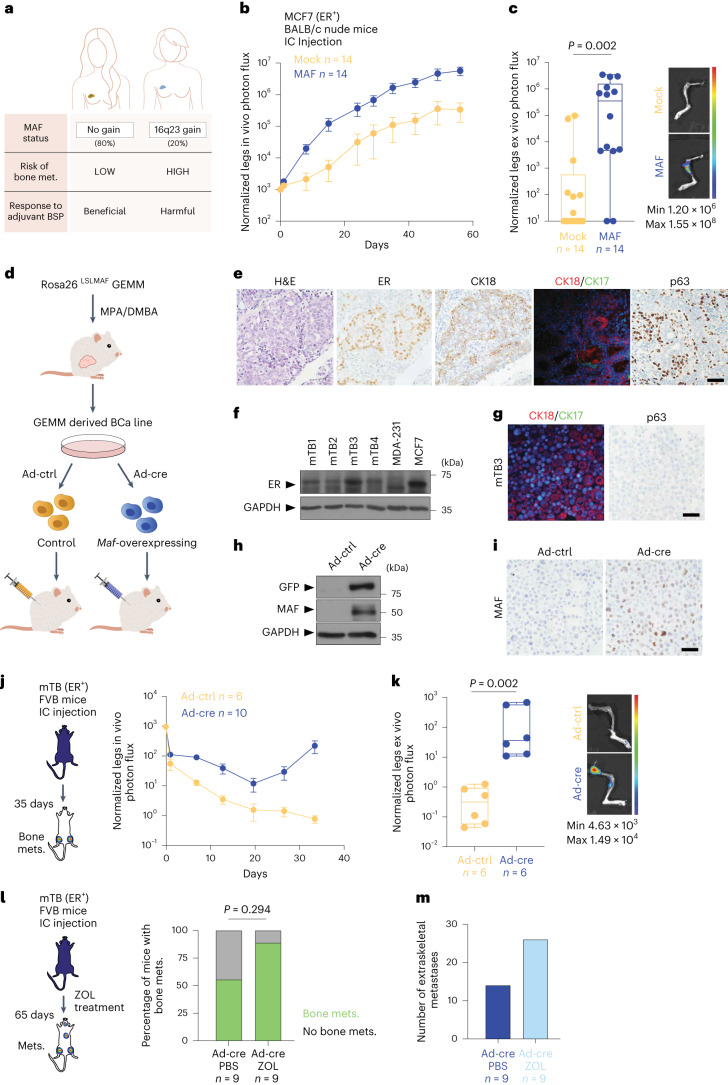
*MAF* amplification drives E2/ER signalling-dependent BCa metastasis. **a**, Schema of the relationship between *MAF* amplification in primary BCa, bone metastasis and bisphosphonate (BSP) treatment response in patients. **b,c**, Normalized photon flux quantification (mean ± s.e.m.) of bone metastasis in vivo, in BALB/c nude mice injected intracardially (IC) with control (mock, *n* = 14 limbs) or *MAF*-overexpressing (MAF, *n* = 14 limbs) MCF7 cells (**b**), and ex vivo, of leg bones from mice in **b** (**c**; representative images). In **c**, the median (centre line), first and third quartiles (box limits) and the minimum to maximum values (whiskers) are shown. Statistical significance determined by two-tailed Mann–Whitney test. The colour scale indicates the intensity of radiance. **d**, Experimental design for obtaining control and *Maf*-overexpressing mTB BCa cells. GEMM, genetically engineered mouse model. **e**, Representative paraffin sections of MPA-DMBA-induced mBCa tumours stained with H&E or against ER, CK18, CK17 or p63. Scale bar, 50 µm. **f**, Immunoblot analysis of ER protein expression in mTB-derived cell lines. MCF7 cells, positive control; MDA-MB-231 cells, negative control; GAPDH, loading control. **g**, Representative paraffin sections of mTB-derived cell pellets stained for CK18, CK17 or p63. Scale bar, 50 µm. **h**,**i**, Control and *Maf*-overexpressing mTB-derived cell lines (see **d**) at five days after infection, analysed by immunoblot for MAF and green fluorescent protein (GFP) expression (**h**), and as MAF-stained paraffin sections (**i**). GAPDH, loading control (**h**). Scale bar, 50 µm (**i**). **j**, Left: schema of the bone colonization experiment. Right: bone lesion in vivo photon flux quantification (mean ± s.e.m.), control (*n* = 6 limbs) or *Maf*-overexpressing (*n* = 10 limbs) mTB cells. **k**, Normalized photon flux of ex vivo legs of mice injected intracardially with control (*n* = 6 limbs) or *Maf*-overexpressing (*n* = 6 limbs) mTB cells. Representative images are shown. The median (centre line), first and third quartiles (box limits) and the minimum to maximum values (whiskers) are shown. Statistical significance determined by a two-tailed Mann–Whitney test. The colour scale indicates the intensity of radiance. **l**, Zoledronic acid (ZOL) experiment overview (left) and quantification of bone homing by MAF cells in mice treated with vehicle (PBS) (*n* = 9 mice) or ZOL (*n* = 9 mice) at day 65 (right). Statistical significance determined by a two-tailed Fisher’s exact test. **m**, Extraskeletal metastasis quantification in mice after intracardiac injection with mTB *Maf*-overexpressing cells with vehicle (*n* = 9 mice) or ZOL (*n* = 9 mice) at day 65. Source data

Amplification of *MAF* (16q23) (>2.5 copies per cell), as well as an increase in its expression levels, occurs in 20% of patients with BCa (herein termed *MAF*-positive) and is associated with increased risk of metastasis, particularly to bone, as well as shorter disease-free survival (DFS) and overall survival (OS), as demonstrated in two phase III clinical trials^10–12^ (Fig. 1a). Adjuvant treatment with bisphosphonates can prevent bone metastasis and significantly improve both five-year DFS and OS following primary tumour resection, but only in patients with non-amplified *MAF* (herein termed *MAF*-negative) BCa. Strikingly, however, adjuvant bisphosphonate treatment of *MAF*-positive patients is relatively ineffective and may even be harmful for pre-menopausal women, suggesting an interaction between *MAF* amplification and systemic hormone levels in the bone-metastatic process^10–12^.

In this Article we investigate a possible mechanistic role of E2, the ER ligand, that is one of the principal hormones affected by menopause, in BCa metastasis, and specifically in the context of *MAF* amplification. Using in vivo models of BCa metastasis, we demonstrate that *MAF*-positive cells are epigenomically primed to facilitate and enhance ER signalling, which subsequently promotes E2-driven metastasis. Inhibition of the histone demethylase KDM1A reverts this priming and causes a substantial reduction of *MAF*-driven BCa bone metastasis. Our findings may have important implications for the effectiveness of clinical treatment choices for ER-positive and/or *MAF*-positive/negative patients with BCa.

## Results

### *MAF* overexpression promotes bone metastasis in ER^+^ BCa cells

**Extended Data Fig. 1:**
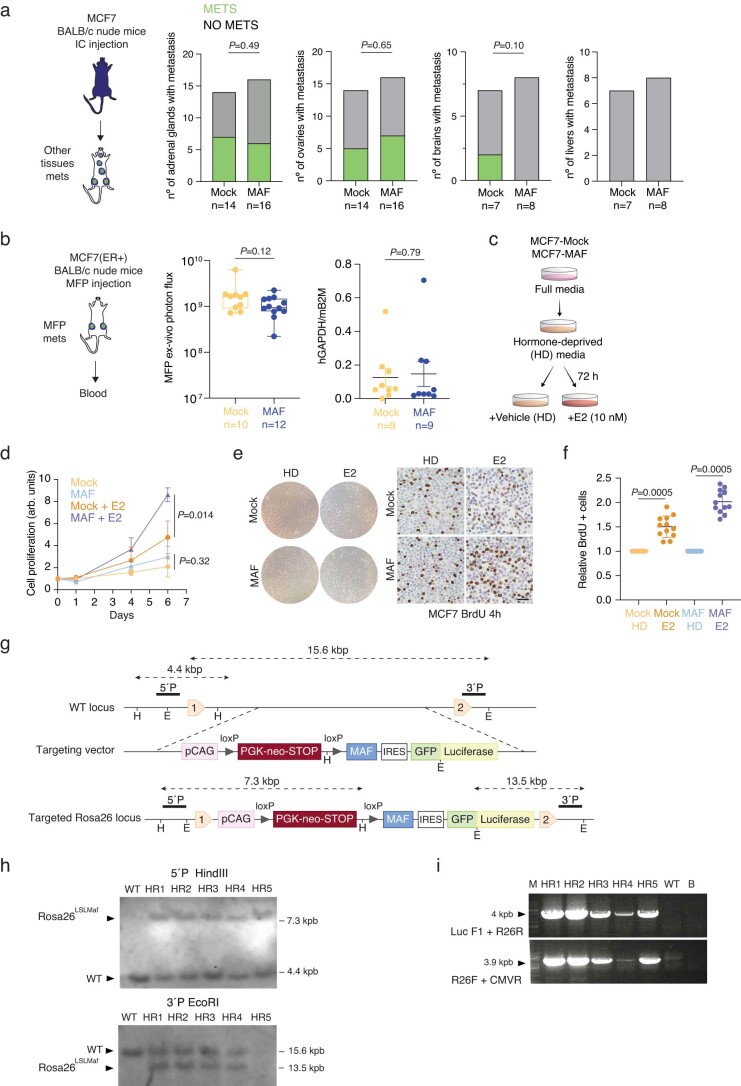
MAF overexpression metastasis models. **a**, Schema of the tissue-colonization experiment (left). Quantification of metastasis in adrenal glands (Mock *n* = 14, MAF *n* = 16), ovaries (Mock *n* = 14, MAF *n* = 16), brain (Mock *n* = 7, MAF *n* = 8) and liver (Mock *n* = 7, MAF *n* = 8), from mice injected with control or *MAF*-overexpressing MCF7 cells (right). Statistical significance, two-tailed Fisher’s exact test. **b**, Schema of circulating tumor cell experiment. Normalized *ex vivo* photon flux quantification of mammary fat pad (MFP) growth in BALB/c mice injected with control (*n* = 10 mice) or *MAF*-overexpressing (*n* = 12 mice) MCF7 cells. The median (centre line), first and third quartiles (box limits) and the minimum to maximum values (whiskers) are shown (left). MCF7 Mock and *MAF*-overexpressing circulating cells were measured by qPCR using a human *GAPDH* and mouse *B2m* mRNA probe (mean ± sd) in blood samples obtained from mice bearing mammary fat pad size-matched tumors (*n* = 9 tumors per group) (right). Statistical significances were calculated using two-sided Wilcoxon rank-sum tests. **c**, Schematic representation of the experimental design. Control (mock-infected) and *MAF*-overexpressing MCF7 cells are cultured in hormone-deprived (HD) media containing charcoal-stripped serum for 72 h to deprive cells of E2. Cells are then stimulated with 10 nM E2 or vehicle. **d**, Cell proliferation assay of control and *MAF*-overexpressing MCF7 cells cultured with HD medium and treated with vehicle or 10 nM E2, *n* = 3. Data is shown as mean ± sd from three biologically independent samples. Statistical significance was calculated using an unpaired two-tailed t-test. **e**, Representative images of culture plates (left) and BrdU stainings (right) of MCF7 control (mock-infected) and *MAF*-overexpressing MCF7 cells cultured under HD conditions and after 10 nM E2 administration. Scale bar, 50 μm. **f**, Quantitative analysis of BrdU positive cells per field. Data represents mean ± sd of 3 biological replicates (*n* = 3 fields per experiment). Statistical significance was calculated using a two-sided Wilcoxon rank-sum test. **g**, Gene targeting strategy. The floxed PGK-neo-STOP cassette followed by the *Maf* cDNA and an IRES-eGFP-Luciferase were targeted into the *Rosa26* locus. The position of the external probes (5’P and 3’P) used for Southern blot analysis and relevant restriction enzyme cleavage sites (H, HindII and E, EcoRI) are shown. The diagnostic HindIII DNA fragments for the wild-type (4.4 kbp) and the targeted allele (7.3 kbp) as well as the diagnostic EcoRI DNA fragments for the wild-type (15.6 kbp) and the targeted allele (13.5 kbp) are represented by dotted arrows. **h**, Southern blot analysis of DNA isolated from recombinant ES cell clones carrying wild-type (WT) and/or recombinant alleles (LSLMaf, HR1-HR5). The migration and sizes of the diagnostic HindIII DNA fragments probed with probe 5’P for the WT (4.4 kbp) and the LSLMaf (7.3 kbp) alleles are indicated by arrowheads. The migration and sizes of the diagnostic EcoRI DNA fragments probed with probe 5’P for the WT (15.6 kbp) and the LSLMaf (13.5 kbp) alleles are indicated by arrowheads. **i**, Long PCR analysis of DNA isolated from recombinant ES cell clones carrying WT or LSLMaf (HR1-5) alleles. R26F primer is located in the mouse genome, upstream the knock-in cassette, and CMVR primer binds to the CAG promoter. R26R primer is located in the mouse genome, downstream the knock-in cassette, and LucF primer binds to the luciferase sequence. Lane B, blank control. Lane M, DNA size marker. DNA fragment size is indicated. Source data

To analyse the role of *MAF* in ER^+^ BCa, we first analysed ER^+^ BoM2 BCa cells, selected for bone tropism in mice from a parental ER^+^ MCF7 BCa cell population^13^, for their ability to drive metastasis in vivo. Of note, MCF7 cells may have limitations^14–16^, but do not present confounding interactions between ER and androgen receptor activity, in contrast to other luminal BCa cells (such as T47D and ZR-75)^17^. In vivo, *MAF* overexpression was sufficient to significantly increase the bone metastasis rates of MCF7 cells when inoculated into the left cardiac ventricle of athymic nude mice, with no metastasis differences at other sites (Fig. 1b,c and Extended Data Fig. 1a). Oestrogen supplementation was required for in vivo tumour growth. We found no differences in circulating tumour cells when control MCF7 or *MAF*-positive MCF7 cells were implanted into mammary fat pads, suggesting that MAF supports late events in the metastatic cascade (Extended Data Fig. 1b). Next, we tested whether *MAF* overexpression enhances cell proliferation in vitro in an E2-dependent manner. Cell proliferation stimulated by E2 was enhanced in *MAF*-positive cells as compared to mock-E2-treated cells or hormone-deprived (HD) controls (Extended Data Fig. 1c–f), suggesting a biological interaction between *MAF* expression and E2.

**Extended Data Fig. 2:**
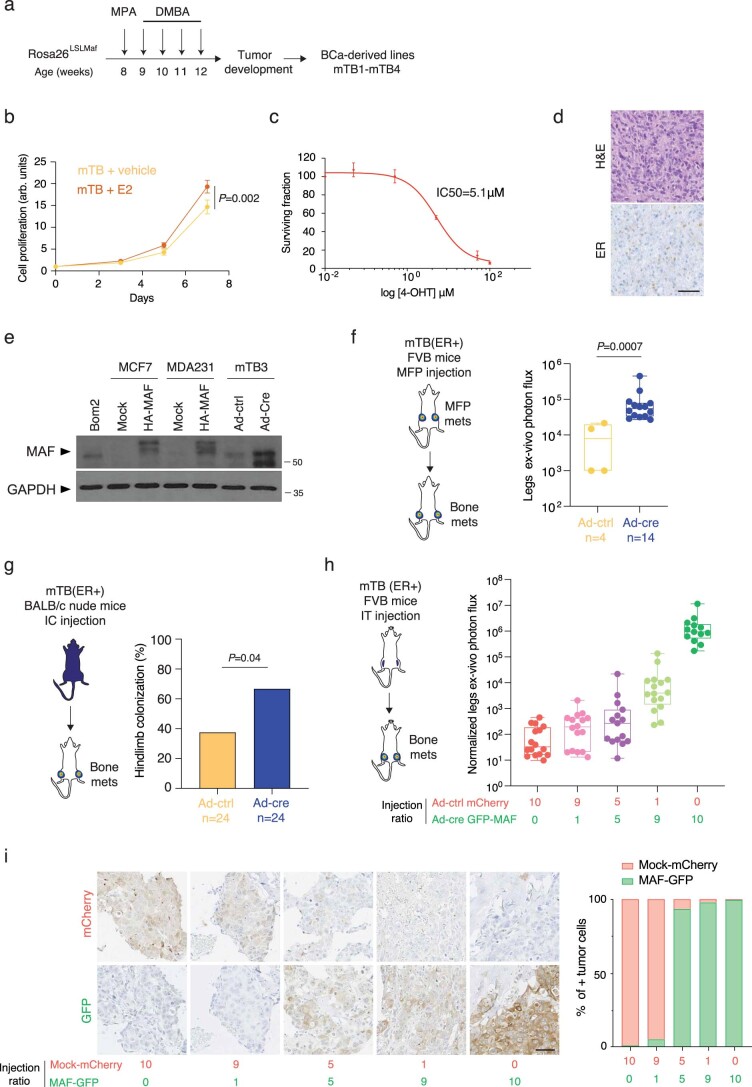
MAF overexpression provides a competitive advantage for bone colonization in ER+ BCa. **a**, Schematic representation of the experimental design to obtain mouse BCa cell lines (mTB1-TB4). **b**, Cell proliferation assay of mTB BCa cells cultured with HD medium and treated with vehicle or 10 nM E2, *n* = 3 biological replicates. Data is shown as mean ± sd. Statistical significance, two-sided Wilcoxon rank-sum test. **c**, 4-OHT dose-response curve (mean ± sd) and IC50 measurement in mTB BCa cells. *n* = 3 biological replicates. **d**, Representative H&E and ER staining in a tumor generated by mTB BCa cells. Scale bar, 50 μm. **e**, Immunoblot showing MAF overexpression in different BCa cell lines. **f**, Schematic representation of the bone-colonization experiment (left). Normalized *ex vivo* leg photon flux of mice injected in the mammary fat pad (MFP) with control (*n* = 4 limbs) or *Maf*-overexpressing (*n* = 14 limbs) mTB cells (right). The median (centre line), first and third quartiles (box limits) and the minimum to maximum values (whiskers) are shown. Statistical significance, two-tailed Mann–Whitney test. **g**, Schematic representation of the bone-colonization experiment (left). Quantification of bone colonization by control (*n* = 24 limbs) and *Maf*-overexpressing (*n* = 24 limbs) mTB cells at day 25 (right). Statistical significance, two-tailed Fisher’s exact test. **h**, Schematic representation of the bone-colonization experiment (left). Normalized *ex vivo* leg photon flux of mice injected intratibially with a mixture of Ad-ctrl-mCherry and Ad-cre MAF-GFP cells at different ratios (10:0 *n* = 16 limbs; 9:1 *n* = 16 limbs; 5:5 *n* = 15 limbs; 1:9 *n* 16 limbs; 0:10 *n* = 13 limbs)(right). The median (centre line), first and third quartiles (box limits) and the minimum to maximum values (whiskers) are shown. **i**, Representative staining images of mCherry and GFP (left) and quantification of mock (mCherry) and GFP-positive metastatic cells (right) in sized-matched bone metastasis from mice injected intratibially with a mixture of different proportions of control and MAF-GFP MCF7 cells. Scale bar, 50 μm (left); *n* = 3 limbs in each group (right). Source data

We next generated a conditional *Maf* overexpression knock-in mouse model (*Rosa26*^LSLMaf^) to (1) provide an independent approach, (2) rule out the presence of acquired genetic alterations in the human BCa cell lines and (3) directly test the effects of increased *MAF* expression in E2-dependent BCa metastasis seeding and progression (Extended Data Fig. 1g–i). To induce mouse BCa tumours, we treated *Rosa26*^LSLMaf^ mice with medroxyprogesterone acetate and 7,12-dimethylbenzanthracene (MPA-DMBA) using an established BCa chemical carcinogenesis protocol^18^ (Fig. 1d,e and Extended Data Fig. 2a). We expanded the mouse tumour-derived cell lines (mTB cells) in vitro; of note, these retained ER and keratin expression (Fig. 1f,g) and ER functional response (Extended Data Fig. 2b–d). *Maf* was then induced by infection with Ad-Cre particles (or not induced, as a control), giving us an isogenic cell-line pair with identical genomic background with or without *Maf* overexpression (Fig. 1d,h,i and Extended Data Fig. 2e). *Maf*-positive mTB cells gave rise to substantially more bone metastasis than the control *Maf*-negative counterparts, irrespective of injection site (Fig. 1j,k and Extended Data Fig. 2f). These findings were corroborated in T-cell-deficient nude mice (Extended Data Fig. 2g), ruling out that the increased bone metastasis potential of *Maf*-overexpressing cells was due to differential activation of the adaptive immune system. Finally, we confirmed that *Maf*-induced bone metastasis was refractory to bisphosphonate treatment, and that extraskeletal metastases (lung, liver, kidney, ovarian and brain) were significantly promoted, as described for patients^12^ (Fig. 1l,m).

By co-mixing different ratios of ER^+^ mTB cells with or without *Maf* amplification, we found that *Maf*-positive cells were required for bone colonization and represented the bulk of cells colonizing bones at all dilutions (Extended Data Fig. 2h). We then analysed sized-matched bone lesions from control or *MAF*-expressing MCF7 cells, co-mixed at different ratios; this revealed that *MAF*-positive cells dominated bone colonization unless an overwhelming amount of control cells were initially present (Extended Data Fig. 2i). Collectively, these results confirm that *MAF*-positive cells have a competitive advantage in the bone colonization process, which can be co-opted by *MAF*-negative cells, generating heterogeneous metastasis dominated by the *MAF*-positive clones. Thus, we next focused on elucidating (1) the molecular mechanisms of the interactions between E2 signalling and *MAF*-overexpression in ER^+^ BCa cells and (2) how these could promote bone metastasis.

### MAF, ER and chromatin factors interact in ER^+^ BCa cells

**Fig. 2:**
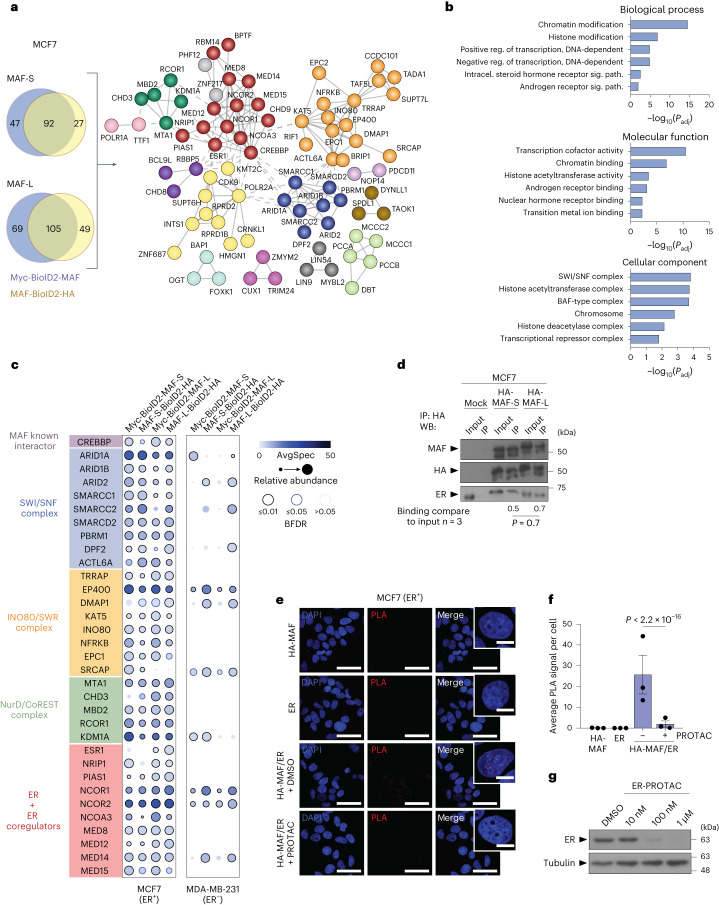
MAF interacts with the ER transcriptional complex. **a**, Network diagram of high-confidence MAF interactors (BFDR < 0.02; spectral counts show a threefold enrichment in BioID2-MAF samples as compared to the myc-BioID2 control) identified in MCF7 cells using BioID. Four BioID2-MAF fusion proteins (N- or C-terminal fusion, MAF-S or -L isoforms) were used as baits. The Venn diagrams show the MAF interactors discovered with each bait. The STRING database was used for visual representation using publicly available protein interaction data. Network edges indicate high-confidence protein–protein associations. Disconnected nodes in the network are hidden. The Markov cluster (MCL) algorithm was used. *n* = 2 biological replicates. For protein–protein interaction enrichment, *P* < 1 × 10^−16^ (two-sided). **b**, Biological process, molecular function and cellular component GO analyses of MAF interactors. Hypergeometric test, one-tailed. Significance was defined using the adjusted *P* value with the Benjamini and Hochberg multiple testing correction. **c**, Bait–prey dot plot for MAF interactors. Selected high-confidence MAF interactors discovered by BioID in MCF7 cells are organized by protein complexes or according to their known functions. Abundances are visualized in MCF7 and MDA-MB-231 cells. Dot colours indicate the average spectral counts for each indicated interactor. Dot size indicates the relative abundance of the interactor across the four different baits. Edge colour shows the BFDR value associated with each bait–prey interaction. **d**, Representative immunoblot (WB, western blot) showing anti-HA co-IP of endogenous ER with HA-tagged MAF (S or L isoforms). Co-immunoprecipitated ER band densities are normalized to the input. The means of three different experiments are shown below. Statistical significance determined by a two-tailed Mann–Whitney test. **e**, PLA of the HA or ER antibody, alone or together, in MAF-overexpressing MCF7 cells treated with DMSO or ER-PROTAC (1 μM) at 24 h before fixation. Representative confocal microscopy images for the PLA red signal and DAPI nuclear staining (with zoomed insets) are shown. Scale bar, 50 μm. Inset scale bar, 10 μm. **f**, PLA signal quantification. Each dot represents the average PLA signal from 123 to 201 nuclei per condition. *n* = 3 biological replicates. Bars represent mean ± s.e.m. Statistical significance determined by two-tailed Wilcoxon rank-sum test. **g**, Representative immunoblot showing ER degradation by different concentrations of ER-PROTAC in MCF7 cells. Tubulin, loading control. Source data

**Extended Data Fig. 3:**
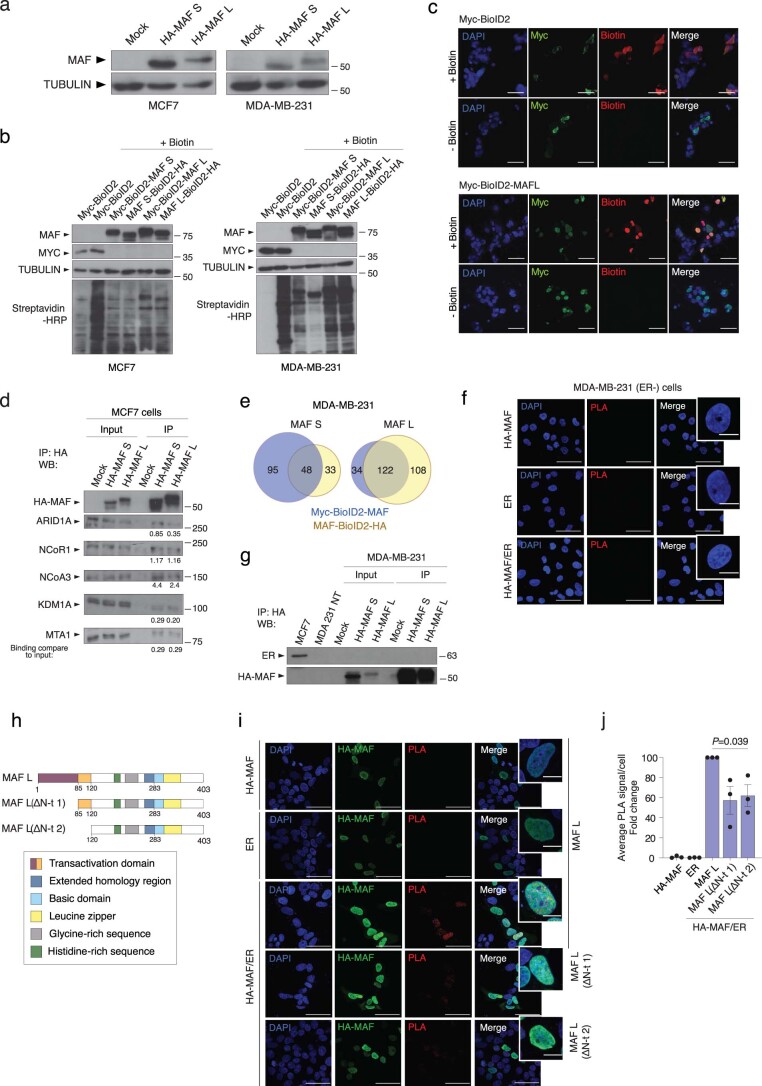
MAF interacts with ER through its N-terminal transactivation domain in ER+ BCa cells. **a**, Representative immunoblot showing MAF expression in control (mock-infected) or *MAF*-overexpressing (S and L isoforms) MCF7 cells (left), or MDA-MB-231 cells (right). Tubulin was used as a loading control. **b**, Representative immunoblot showing the expression of myc-BioID2 alone and BioID2-tagged MAF proteins (N- or C-terminal fusion, MAF S or L isoforms) in MCF7 cells (left) and MDA-MB-231 cells (right) analysed after 24 h biotin supplementation. Expression of the BioID2 biotin ligase leads to biotinylation of endogenous proteins, detected using streptavidin-HRP. **c**, Representative immunofluorescence images of MCF7 cells transfected with myc-BioID2 or myc-BioID2-MAF L plasmids. The myc-BioID2 tag alone is distributed throughout the nucleus and cytoplasm (top), whereas the myc-BioID2-MAF L fusion protein localizes primarily in the nucleus (bottom). Biotinylated proteins, detected with fluorescently-labelled streptavidin, colocalize with BioID2 when cells are cultured with excess biotin (50 μM). DNA is labelled with DAPI. Scale bar, 50 μm. **d**, Immunoblot showing anti-HA co-immunoprecipitation (co-IP) with HA-tagged MAF (S or L isoforms) of endogenous ARID1A, NCoR1, NCoA3, KDM1A and MTA1. Co-IP band densities were normalized to each respective input. **e**, Venn diagrams showing high-confidence MAF interactors (BFDR < 0.02 and spectral counts with a 3-fold enrichment in BioID2-MAF samples compared to the myc-BioID2 control) discovered by BioID in MDA-MB-231 cells. Four BioID2-MAF fusion proteins (N- or C-terminal fusion, MAF S or L isoform) were used as baits. *n* = 1 biological replicate. **f**, PLA of HA or ER antibody alone or both antibodies together in MAF L-overexpressing MDA-MB-231 cells. Representative confocal microscopy images for PLA red signal and DAPI nuclear staining with magnified inset are shown. Scale bar, 50 μm; inset scale bar, 10 μm. **g**, Immunoblot showing HA co-IP of HA-tagged MAF (S or L isoforms) with endogenous ER in MDA-MB-231 cells. **h**, Schematic representation of full-length MAF L and two N-terminal truncation mutants. **i**, PLA of HA or ER antibody alone or both antibodies together in MCF7 cells transfected with full-length HA-tagged MAF L and N-terminal truncation mutants 24 h prior to fixation. Representative confocal microscopy images for HA-MAF expression (green), PLA signal (red) and DAPI nuclear staining with magnified inset are shown. Scale bar, 50 μm; inset scale bar, 10 μm. **j**, PLA signal quantification. Each dot represents the average PLA signal from 65 to 135 nuclei per condition. *n* = 3 biological replicates. Bars represent mean ± sem. Statistical significance, two tailed Wilcoxon rank-sum test. Source data

*MAF* encodes an AP1 family transcription factor (TF), and its DNA binding (and thus its pro-metastatic activity) may depend on interaction partners with cell context-specific expression^19^. To identify proteins that interact with MAF in living BCa cells in an unbiased manner, we performed proximity-dependent biotin identification (BioID2^20,21^), which allows weak and transient interactions to be detected. Although not functionally distinct, we used both short (MAF-S) and long (MAF-L) isoforms to avoid any potential isoform-dependent biases. Each MAF isoform was fused in-frame to the N or C terminus of BioID2, resulting in MAF-BioID2-HA and myc-BioID2-MAF, respectively (Fig. 2a). MAF-BioID2 fusions were expressed in both MCF7 (ER^+^) and MDA-MB-231 (ER^−^) cells, resulting in the correct nuclear localization and in vivo biotinylation after biotin supplementation (Extended Data Fig. 3a–c). Following a streptavidin pulldown from biotin-treated cells containing BioID2-MAF or (as a control) BioID2, co-precipitated proteins were identified by tandem mass spectrometry (nanoLC-MS/MS). We obtained numerous high-confidence interactors (Methods): 139 for N-terminal-tagged MAF-S, 119 for C-terminal-tagged MAF-S, 174 for N-terminal-tagged MAF-L and 154 for C-terminal-tagged MAF-L (Fig. 2a). We then selected 92 interactors common to both MAF-S conditions, and 105 interactors common to both MAF-L conditions, to define a network of 126 MAF high-confidence interactors (of which 71 were common in all four conditions). This set of 126 included the well-characterized MAF interactor CREBBP^22^ and was strongly enriched (*P* < 1 × 10^−16^) for a highly interconnected network of known protein–protein interactions, indicating the existence of biologically relevant complexes among the MAF interactors (Fig. 2b). Indeed, some of the major chromatin-remodelling complexes, such as SWI/SNF, INO80, NurD and CoREST, were present. Gene ontology (GO)^23^ analyses also identified molecular functions that influence gene expression, including members of the hormone receptor signalling family. Notably, ER itself emerged as a biologically relevant MAF interactor (Fig. 2a–c). ER and other interactors were subsequently validated by co-immunoprecipitation (co-IP; Fig. 2d and Extended Data Fig. 3d). To address whether the presence of ER influences MAF interactions, BioID was performed using ER^−^ MDA-MB-231 BCa cells (Fig. 2c and Extended Data Fig. 3b,e). In these ER^−^ cells, MAF retained its interactions with some components of the SWI/SNF, INO80, NurD and CoREST chromatin remodelling complexes, but not with ER and/or critical ER coactivators (Fig. 2c and Extended Data Fig. 3f,g). Collectively, these data suggest that interactions between MAF and specific partners are cell type-dependent.

### The N-terminal transactivation domain of MAF interacts with ER

**Extended Data Fig. 4:**
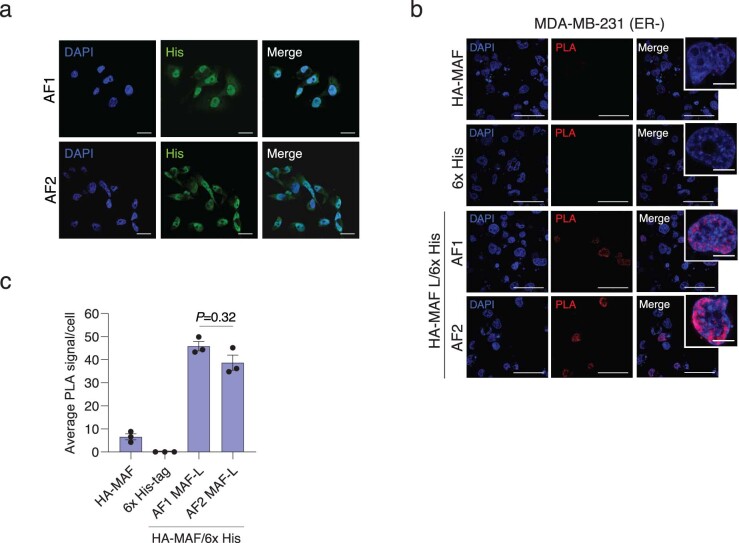
Interaction of ER AF1 and AF2 domains with MAF. **a**, Immunofluorescence showing the nuclear localization of AF1 and AF2 ER domains through the detection of the 6x His-tag. Scale bar, 20 μm. **b**, PLA of HA or 6x His-tag antibody alone or both antibodies together in MAF-overexpressing MDA-MB-231 cells transduced with AF1 or AF2 ER domains. Representative confocal microscopy images for PLA red signal and DAPI nuclear staining with magnified inset are shown. Scale bar, 50 μm; inset scale bar, 10 μm. **c**, PLA quantification. Each dot represents the average PLA signal from 64 to 208 nuclei per condition. *n* = 3 biological replicates. Bars represent mean ± sem. Statistical significance, two-tailed Wilcoxon rank-sum test. Source data

We next validated the MAF interactions by co-IP in MCF7 cells using HA-tagged MAF-S and MAF-L (Fig. 2d and Extended Data Fig. 3d). Using proximity ligation assays (PLAs), we then confirmed the MAF–ER interactions and colocalization, with significantly higher fluorescence signals detected and quantified in cell nuclei with the combination of HA and ER antibodies as compared to the single antibody controls (Fig. 2e,f). Based on an induced degradation of ER using an ER-specific PROTAC^24^ in MCF7 ER^+^ BCa cells, we confirmed the specificity of the MAF–ER interactions (Fig. 2e–g). We then used PLA to investigate the protein domain of MAF that interacts with ER by comparing the full-length MAF-L with truncated versions lacking part or all of the transactivation domain (ΔN-t 1, aa 85–403 and ΔN-t 2, aa 120–403, respectively)^19^. The truncated versions had similar interactions with ER that were weaker than the full-length isoform, suggesting that ER interacts within aa 1–85 of the MAF transactivation domain (Extended Data Fig. 3h–j). Both the ligand-binding domain (AF2, aa 310–595) and transactivation domain (AF1, aa 1–183) of ER were located in the cell nucleus (Extended Data Fig. 4a) and interacted with MAF when tested in PLA assays (Extended Data Fig. 4b,c). However, we cannot exclude that interactions with the ER AF1 or AF2 domain have different roles, as previously described^25^.

### The MAF–ER interaction results in transcriptional reprogramming

**Fig. 3:**
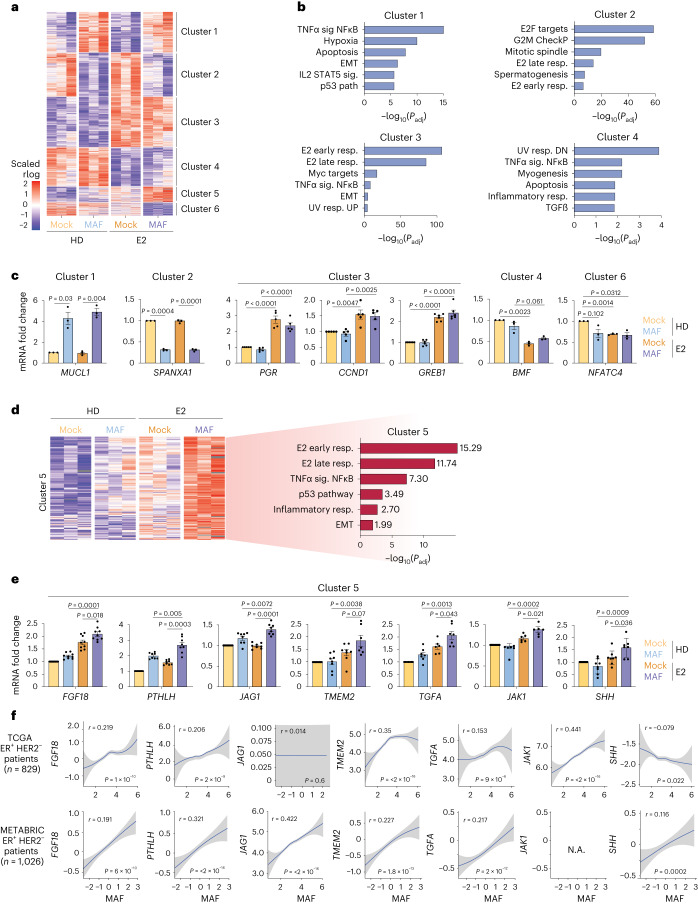
MAF regulates the E2/ER-induced metastasis transcriptional gene program. **a**, RNA-seq heatmap of differentially expressed genes in *MAF*-overexpressing compared to control (mock-infected) MCF7 cells, HD or E2-treated (10 nM, 6 h). Expression profiles are grouped in six clusters based on comparisons between the four conditions. Cluster 1, MAF upregulated genes; cluster 2, MAF downregulated genes; cluster 3, E2 upregulated genes; cluster 4, E2 downregulated genes; cluster 5, E2 and MAF upregulated genes; cluster 6, E2 and MAF downregulated genes. The colour scale indicates expression levels. *n* = 3 biological replicates. **b**, Associated GO terms for genes in the clusters in **a**. Hypergeometric test, one-tailed. Significance is defined by the adjusted *P* value using the Benjamini and Hochberg multiple testing correction. **c**, Quantitative reverse transcriptase-polymerase chain reaction (qRT-PCR) expression analysis of selected genes of clusters 1–4 and 6 in control (mock-infected) and *MAF*-overexpressing MCF7 cells, either HD or E2-treated. Expression is normalized to the housekeeping gene *GAPDH*. *MUCL*, *n* = 3; *SPANXA1*, *n* = 3; *PGR*, *n* = 5; *CCDN1*, *n* = 5; *GREB1*, *n* = 6; *BMF*, *n* = 3; *NFATC4*, *n* = 3. Three to six biological replicates per gene. Data are presented as mean ± s.e.m. *P* values were calculated using a two-sided *t*-test. **d**, Differentially expressed genes in cluster 5 as determined by RNA-seq (left) (see also **a**) and significantly enriched hallmark GO terms for the indicated cluster (right). Hypergeometric test, one-tailed. Significance was defined by the adjusted *P* value using the Benjamini and Hochberg multiple testing correction. **e**, qRT–PCR expression analysis of selected genes of cluster 5 in control (mock-transfected) and *MAF*-overexpressing MCF7 cells, either HD or E2-treated. Expression was normalized to the housekeeping gene *GAPDH*. *FGF18*, *n* = 9; *PTHLH*, *n* = 8; *JAG1*, *n* = 8; *TMEM2*, *n* = 8; *TGFA*, *n* = 7; *JAK1*, *n* = 6; *SHH*, *n* = 7. Six to nine biological replicates per gene. Data are presented as mean ± s.e.m. *P* values were calculated using a two-sided *t*-test. **f**, Correlation of *MAF* expression with *FGF18*, *PTHLH*, *JAG1*, *TMEM2*, *TGFA*, *JAK1* and *SHH* in patients with ER^+^ HER2^−^ BCa. Gene expression data were retrieved from TCGA^39^ and METABRIC^38^. Data are presented as mean ± s.d. NA, not available. Correlation was determined using the two-tailed Spearman’s correlation test. Unadjusted *P* values. Source data

**Extended Data Fig. 5:**
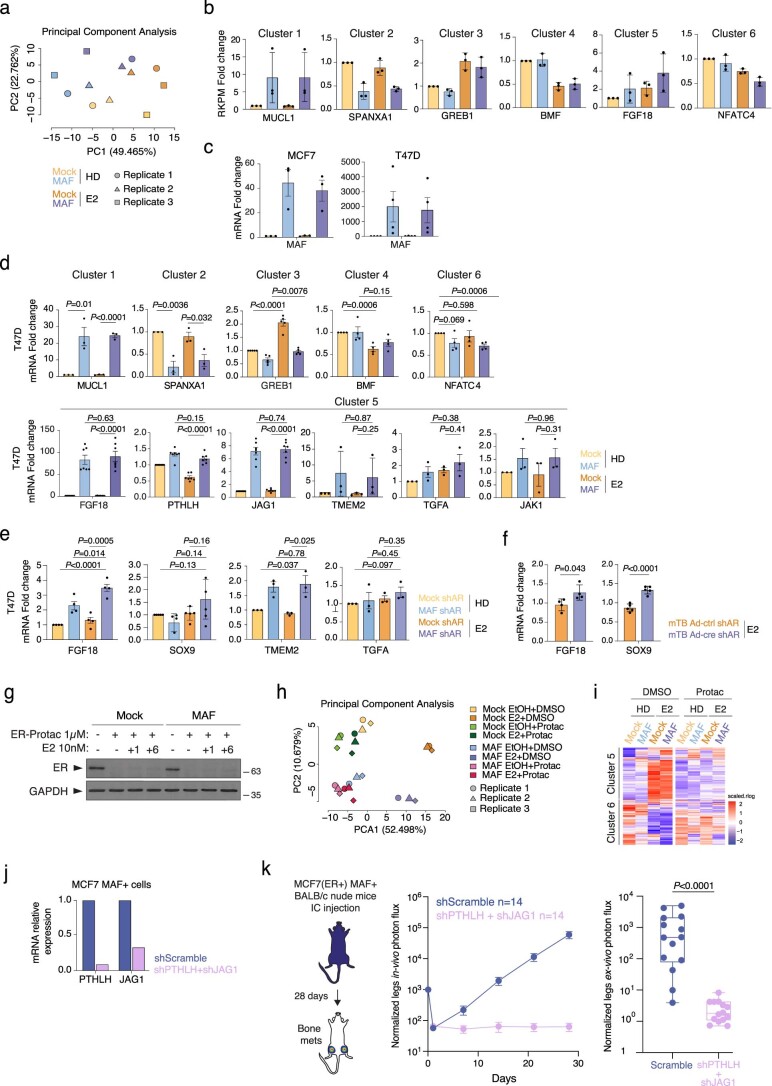
MAF-E2 induced transcriptional changes in BCa cell lines and ER dependency. **a**, Principal component analysis (PCA) on the rlog normalized expression matrix (showing first two components). Batch effect was adjusted gene-wise using a linear model. **b**, RNA-seq expression of a selected gene from each cluster represented by fold-change of RPKM values. Data represents the average of 3 biological replicates. **c**, Analysis of MAF expression in control and MAF-overexpressing MCF7 and T47D cell lines under hormone deprivation (HD) conditions and after 10 nM E2 administration by qRT-PCR. Expression was normalized to the housekeeping gene *GAPDH*. *n* = 3 independent biological replicates. Data are mean ± s.d. **d**, qRT-PCR expression analysis of a selected gene in each cluster in control (mock-infected) and MAF-overexpressing T47D cells under HD conditions and after 10 nM E2 administration. Expression was normalized to the housekeeping gene *GAPDH*. *MUCL, n* = 3; *SPANXA1*, *n* = 3; *GREB1*, *n* = 5; *BMF*, *n* = 4; *NFATC4*, *n* = 4; *FGF18*, *n* = 7; *PTHLH*, *n* = 7; *JAG1*, *n* = 7; *TMEM2*, *n* = 3; *TGFA*, *n* = 3 and *JAK1*, *n* = 3. 3 to 7 biological replicates per gene. Data are mean ± s.d. The *P* value was calculated using a two-sided *t*-test. **e**, qRT-PCR expression analysis of selected genes in control (mock-infected) and MAF-overexpressing T47D cells transduced with Androgen Receptor (AR)-targeted shRNA and cultured under HD conditions and after 10 nM E2 administration. Expression was normalized to the housekeeping gene *GAPDH*. *n* = 3 independent biological replicates. Data are mean ± s.d. The *P* value was calculated using a two-sided *t*-test. **f**, qRT-PCR expression analysis of selected genes in control (Ad-ctrl) and MAF-overexpressing (Ad-cre) mouse BCa cells transduced with AR-targeted shRNA and cultured under HD conditions or E2 treated (10 nM). Expression was normalized to the housekeeping gene *GAPDH*. *n* = 3 independent biological replicates. Data are mean ± s.d. The *P* value was calculated using a two-sided *t*-test. **g**, Immunoblot showing ER degradation by using ER-PROTAC in control (mock) and MAF-overexpressing MCF7 cells 1 and 6 hours after E2 stimulation. GAPDH was used as a loading control. **h**, Principal component analysis (PCA) on the rlog normalized expression matrix (showing first two components). Batch effect was adjusted gene-wise using a linear model. **i**, RNA-seq heatmap of differentially expressed genes, already defined clusters 5 and 6, in MAF-overexpressing compared to control (mock-infected) MCF7 cells upon ER degradation followed by administration of E2 (10 nM for 6 h). Color scale indicates expression levels. Red indicates upregulation and blue shows downregulation. *n* = 3 biological replicates. **j**, qRT-PCR expression analysis of *PTHLH* and *JAG1* in MAF-overexpressing MCF7 cells transduced with *PTHLH* and *JAG1*-targeted shRNAs. Expression was normalized to the housekeeping gene *GAPDH*. **k**, Schematic representation of the experimental design. Normalized *in vivo* photon flux quantification (mean ± sem) of bone metastasis in BALB/c nude mice injected intracardially with control (scramble, *n* = 14 limbs) and knockdown of *PTHLH* or *JAG1* (*n* = 14 limbs) in *MAF*-overexpressing MCF7 cells (left). Normalized *ex vivo* photon flux quantification of bone metastasis. The median (centre line), first and third quartiles (box limits) and the minimum to maximum values (whiskers) are shown. Statistical significance, two-tailed Mann–Whitney test (right). Source data

We next performed RNA sequencing (RNA-seq) on MCF7 cells that had been hormone-deprived (HD) or treated with E2 for 6 h (Fig. 3a,b, Extended Data Fig. 5a–c and Supplementary Table 1). We identified transcriptional changes that required (1) *MAF* overexpression (clusters 1 and 2), (2) E2 (clusters 3 and 4) and (3) both MAF and E2 (herein, MAF/E2-dependent; clusters 5 and 6) (Fig. 3a). Consistent with E2-stimulated proliferation (Extended Data Fig. 1d–f and Supplementary Table 2), E2 treatment positively correlated with E2 early and late gene responses (cluster 3) (Fig. 3a,b), including the well-established ER-mediated response genes *GREB1, CCND1* and *PGR* (Fig. 3c). Notably, the MAF/E2-dependent gene signature (cluster 5) positively correlated with E2 early and late responses, inflammation and the epithelial to mesenchymal transition (EMT) gene response, including *PTHLH*, *JAG1*, *FGF18*, *TMEM2*, *TGFA*, *JAK1* and *SHH*; these gene products support metastasis-adapting capabilities, especially to bone^26–37^ (Fig. 3d,e). Consistently, the MAF/E2-dependent responses were observed across different BCa cell lines (Extended Data Fig. 5d). In the T47D and mTB cell lines, some of the E2 gene responses required low androgen receptor (AR), as previously reported^17^ (Extended Data Fig. 5e,f). Importantly, we observed a positive correlation between the expression levels of *MAF* and those of *FGF18*, *PTHLH*, *JAG1*, *TMEM2*, *TGFA*, *JAK1* and *SHH* in ER^+^ BCa-patient gene expression datasets, including the Molecular Taxonomy of Breast Cancer International Consortium (METABRIC) and The Cancer Genome Atlas (TCGA) BCa cohorts^13,38,39^ (Fig. 3f). Of note, when we targeted ER for degradation using ERα-PROTAC^24^, the MAF/E2-dependent gene responses were lost (Extended Data Fig. 5g–i). Furthermore, *PTHLH* and *JAG1* depletion in *MAF*-expressing MCF7 cells significantly reduced bone metastasis in vivo (Extended Data Fig. 5j,k), demonstrating their pro-metastatic functions. Collectively, these data show that *MAF* overexpression expands the transcriptional repertoire of ER^+^ BCa cells upon E2 stimulation in an ER-dependent manner. We propose that *MAF* overexpression supports BCa progression beyond proliferation and primary tumour growth (Extended Data Fig. 1b–f), possibly by directly activating the bone microenvironment to a more metastasis-receptive state.

### MAF redistributes ER on chromatin to target metastasis-associated genes

**Fig. 4:**
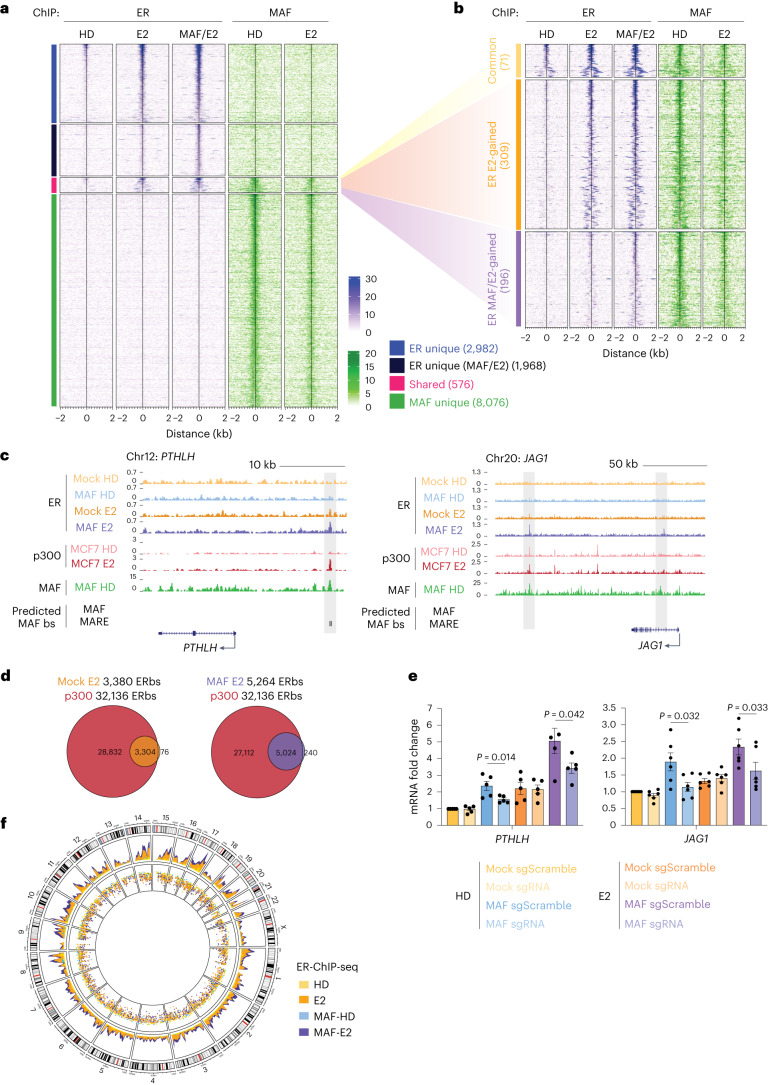
MAF chromatin binding overlaps with and expands binding of ER. **a**, ER and MAF ChIP-seq peaks in control and *MAF*-overexpressing MCF7 cells that were HD or E2-treated (for 1 h). Groups of binding sites are defined based on the positional overlap between ER and MAF binding. Colour scale bars indicate the scale for Reads Per Genome Content (RPGC) normalized coverage (deepTools^58^). The number of peaks is indicated for each group. **b**, Shared MAF/ER binding sites from **a** are divided into common and increased ER binding sites in the presence of E2 (ER E2-gained) or in the presence of E2 and MAF overexpression (ER MAF/E2-gained). **c**, UCSC genome browser (http://genome.ucsc.edu, February 2009 (GRCh37/hg19)) screenshots of ChIP-seq profiles at representative target genes, showing ER ChIP-seq tracks from control and *MAF*-overexpressing MCF7 cells, either HD or E2-treated. p300 ChIP-seq peaks (from ref. ^40^) depict active enhancer regions. MAF ChIP-seq tracks from *MAF*-overexpressing MCF7 cells under HD conditions are shown. Predicted MAF binding sites (using the MAF or MARE matrices) within ER peaks are represented in black. bs, binding sites. **d**, Venn diagrams showing the overlap between p300 and ER binding sites in control (left) or *MAF*-overexpressing MCF7 cells (right) after E2 treatment. **e**, qRT–PCR expression analysis of *PTHLH* (left) and *JAG1* (right) in control (mock-infected < yellow/orange) and *MAF*-overexpressing (blue/purple) MCF7 cells transduced with a lenti-dCas9-KRAB and a lentiGuide-puro expressing sgScramble (dark) or specific sgRNAs against *PTHLH* and *JAG1* herein uncovered enhancer sequences (light). Cells were cultured under HD conditions and stimulated with 10 nM E2. Expression was normalized to the housekeeping gene *B2M*. *n* = 5 (*PTHLH*) and *n* = 6 (*JAG1*) biological replicates. Data are presented as mean ± s.e.m. *P* values were calculated using a one-sided *t*-test. **f**, Circos plot summarizing the chromosomal distribution of ER ChIP-seq reads in control or *MAF*-overexpressing MCF7 cells under HD conditions and after E2 treatment (10 nM, 1 h). The outermost circle represents the ideograms of each chromosome with labels in Mb of physical distance. The inner two circles show the density of ER ChIP-seq reads. Source data

**Extended Data Fig. 6:**
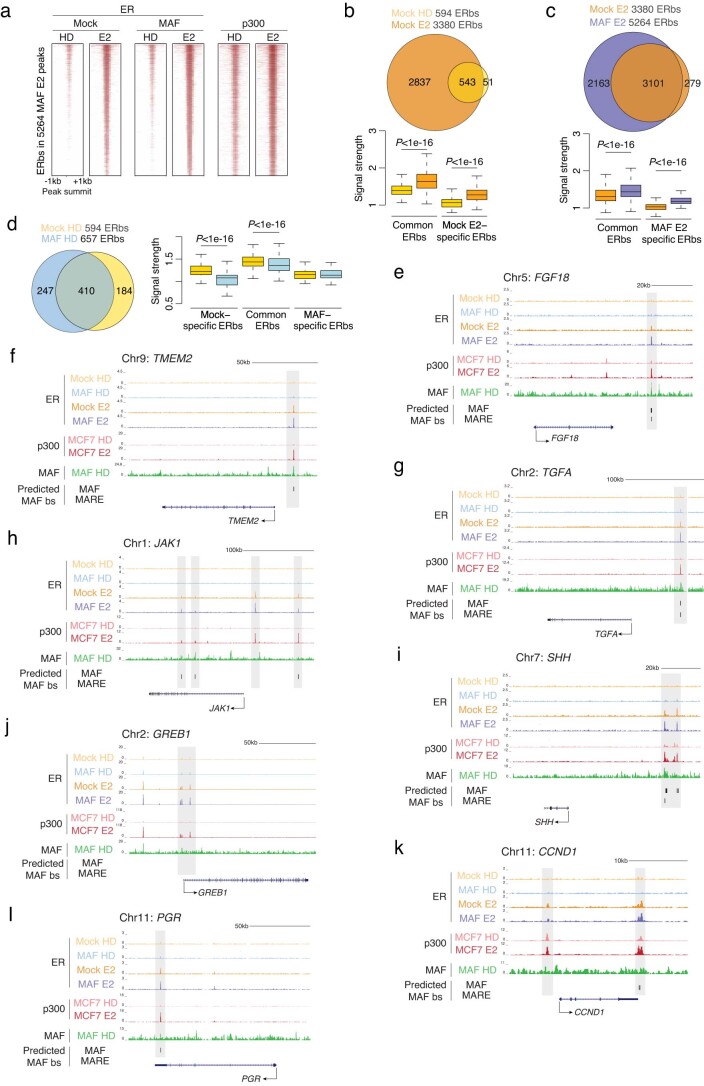
MAF expands ER binding throughout the genome. **a**, ChIP-seq heatmap showing the distribution of ER reads (in control and MAF-overexpressing MCF7 cells) and p300 on the 5264 ER binding sites found in MAF-overexpressing cells after E2 stimulation (peak summit ±1 kb). Enrichment levels were normalized for the total number of mapped reads of each sample. Peaks were ranked by the intensity of ER signal in MAF-overexpressing cells after E2 stimulation. **b**, Venn diagrams showing ER binding sites (ERbs) (ChIP-seq peaks) identified in control (mock-infected) MCF7 cells under hormone deprivation (HD) conditions and after 1 h 10 nM E2 administration (top). Boxplot showing ER ChIP-seq signal intensity for common peaks in both HD conditions and after E2 stimulation (543 peaks) and for peaks that appeared only after E2 administration (2837 peaks) (bottom). Boxes represent the first, second (median) and third quartiles. Whiskers indicate maximum and minimum values with outliers being excluded. *n*= number of peaks (594 in Mock HD, 3380 in Mock E2) from 1 biological replicate per condition. Two-tailed Mann-Whitney test, unadjusted *P* values. **c**, Venn diagrams showing ERbs identified in control and MAF-overexpressing MCF7 cells after 1 h E2 stimulation (top). Boxplot represents ER ChIP-seq signal intensity for common ER binding sites in both conditions (3101 peaks) and for specific ER binding sites in MAF-overexpressing cells (2163 peaks) (bottom). Boxes represent the first, second (median) and third quartiles. Whiskers indicate maximum and minimum values with outliers being excluded. *n*= number of peaks (3380 in Mock E2, 5264 in MAF E2) from 1 biological replicate per condition. Two-tailed Mann-Whitney test, unadjusted *P* values. **d**, Venn diagrams showing ERbs (ChIP-seq peaks) identified in control and MAF-overexpressing MCF7 cells cultured in HD conditions (left). Boxplot represents ER ChIP-seq signal intensity for mock-specific ERbs (184 peaks), common ERbs in both conditions (410 peaks), and MAF-specific ERbs (247 peaks) (right). Boxes represent the first, second (median) and third quartiles. Whiskers indicate maximum and minimum values with outliers being excluded. *n*= number of peaks (594 in Mock HD, 657 in MAF HD) from 1 biological replicate per condition. Two-tailed Mann-Whitney test, unadjusted *P* values. **e to l**, UCSC genome browser (http://genome.ucsc.edu, Feb. 2009 (GRCh37/hg19)) screenshot of ChIP-seq profiles of the indicated genes. ER ChIP-seq tracks from control and MAF-overexpressing MCF7 cells under HD conditions and after E2 stimulation are shown. MAF ChIP-seq tracks from MAF-overexpressing MCF7 cells under HD conditions are shown. p300 ChIP-seq peaks depict active enhancer regions. Predicted MAF binding sites (using the MAF or MARE matrices) within ER peaks are represented in black.

**Extended Data Fig. 7:**
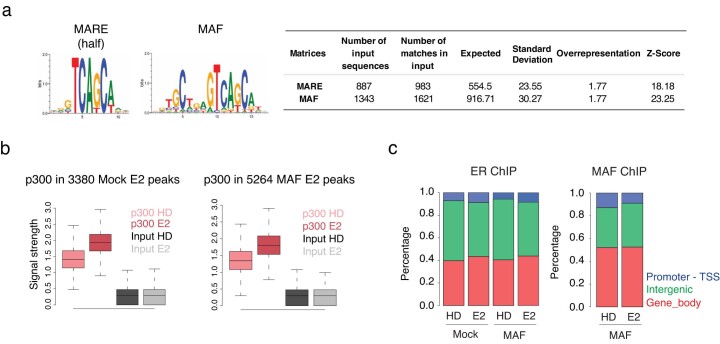
Characterization of ER and MAF chromatin binding regions. **a**, Position-weight matrices of two described MAF binding motifs obtained from the MatBase database (Genomatix software). MARE, MAF response element (half sites) (left). Prediction of MAF binding sites in the vicinity of ER ChIP-seq peaks identified in MAF-overexpressing cells after E2 stimulation generated with the MatInspector program from Genomatix. The number of input sequences with at least one match of the MARE or MAF matrix, the total number of matches in all input sequences, the expected match numbers in an equally sized sample of the genome and its standard deviation, the overrepresentation and the Z-score are shown. A Z-score below –2 or above 2 can be considered statistically significant. **b**, Boxplots showing enrichment of p300, a marker of active enhancers, in ERbs identified in control (left) and MAF-overexpressing MCF7 cells (right) after E2 administration. Boxes represent the first, second (median) and third quartiles. Whiskers indicate maximum and minimum values with outliers being excluded. *n*= number of peaks (3380 in Mock E2, 5264 in MAF E2) from 1 biological replicate per condition. **c**, Percentage of ER ChIP-seq peaks and MAF ChIP-seq peaks annotated according to promoter-TSS / intergenic / gene body.

To gain insight into the genome-wide localization of MAF, and to assess its potential direct interaction with ERα upon E2 treatment, we performed ERα and MAF chromatin immunoprecipitation followed by high-throughput sequencing (chromatin immunoprecipitation followed by sequencing, ChIP-seq) in control and *MAF*-overexpressing MCF7 BCa cells treated or not with E2. Indeed, extensive ER binding to chromatin was observed only after E2 treatment, indicating its role in ER recruitment to chromatin (Fig. 4a and Extended Data Fig. 6a). In contrast, MAF binding was largely independent of E2 (Fig. 4a). Interestingly, however, both ER and MAF bound a shared set of genomic sites (576) in response to E2, of which some (196) were bound by ER only in cells with *MAF* overexpression (Fig. 4a,b and Extended Data Fig. 6a–d). Visual inspection of these loci in the genome browser revealed an E2-induced, MAF-dependent ER binding to previously unknown target sites in enhancers associated with MAF–E2 target metastasis supporting genes, such as *FGF18*, *PTHLH*, *JAG1*, *TMEM2*, *TGFA*, *JAK1* and *SHH* (Fig. 4c and Extended Data Fig. 6e–l; note that *GREB1*, *CCND1* and *PGR* were used as bona fide ER/E2 targets). These putative *cis*-regulatory elements are enriched for MAF consensus binding motifs (MARE and MAF) and coincided with ChIP-seq peaks of the enhancer marker p300^40^ (Fig. 4d and Extended Data Fig. 7a,b). Consistently, an enrichment for ER consensus binding motifs (ERE) coincided with the MAF ChIP-seq peaks (Supplementary Table 3). Annotation of both ER and MAF ChIP-seq binding sites suggests a predominant localization of MAF on intergenic and intronic potential enhancer regions (Extended Data Fig. 7c).

Next, we used CRISPR interference (CRISPRi) to repress these putative MAF response elements in the vicinity of the *PTHLH* and *JAGGED1* transcription start sites (TSSs) to confirm their functional role. Upon inactivation of the shared MAF/ER peak site using a specific single guide RNA (sgRNA) against the site and dCas9, the MAF/ER-dependent transcriptional induction of *PTHLH* and *JAGGED1* was significantly blunted, irrespective of E2 (Fig. 4e). Overall, our data suggest that the presence of MAF increases ER-binding to chromatin, either directly or indirectly. In addition, MAF interacts directly with ERα, so its overexpression in BCa associates with and expands the ER cistrome (Fig. 4f). We hypothesize that *MAF* overexpression, through its direct interaction with chromatin remodellers (Fig. 2c), initiates chromatin priming, which, in response to E2, promotes ER gene regulation, ultimately facilitating the metastatic process of ER^+^ BCa.

### ER/MAF cooperation confers a specific chromatin landscape

**Fig. 5:**
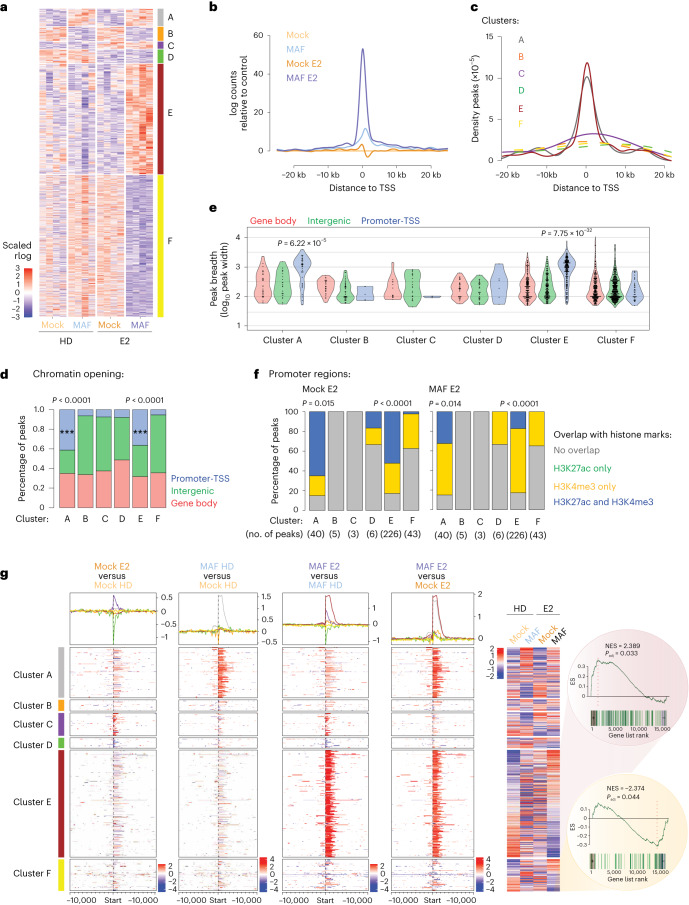
*MAF* amplification causes a change in the chromatin landscape. **a**, Heatmap of ATAC-seq normalized data showing most differential accessible peaks in *MAF*-overexpressing compared to control (mock-infected) MCF7 cells under HD or E2-treated (10 nM) conditions. Cluster A, MAF-dependent open chromatin; cluster B, MAF-dependent closed chromatin; cluster C, E2-dependent open chromatin; cluster D, E2-dependent closed chromatin; cluster E, MAF/E2-dependent open chromatin; cluster F, MAF/E2-dependent closed chromatin regions. *n* = 4 biological replicates. The colour scale indicates opening levels (regularized log (rlog) + *z*-score normalization of counts per peak matrix). **b**, ATAC-seq signals that were centred on TSSs in control (mock-infected) and *MAF*-overexpressing MCF7 cells, either HD or E2-treated (10 nM); signals were normalized to mock HD. **c**, Density distribution of distances to TSSs for peaks according to the clusters from **a**. **d**, Percentage of annotated peaks (promoter-TSS/gene body/intergenic) in the different cluster groups from **a**. Statistical significance determined by a permutation test (one-sided). **e**, Peak breadth plot according to peak annotation (promoter-TSS/gene body/intergenic) and cluster (from **a**). Statistical significance determined by two-sided Wilcoxon rank sum test. **f**, Plot depicting the percentage of promoter-TSS annotated ATAC-seq peaks from ATAC-seq data corresponding to clusters (from **a**) that overlap with H3K27ac (active enhancers and promoters), H3K4me3 (active promoters) or both (active promoters—broad peaks). Statistical significance determined by one-sided permutation test. **g**, Integration of ATAC-seq and RNA-seq data. For comparison, only peaks annotated as promoter-TSS were considered. ATAC-peak candidates were assigned a gene using Homer annotations^59^; gene signatures corresponding to each cluster were used to perform Gene Set Analysis (GSA) on the RNA-seq data. The colour scale indicates DESeq2 test statistics in peaks near TSSs (±10 kb). ES, Enrichment Score; NES, Normalized Enrichment Score.

**Extended Data Fig. 8:**
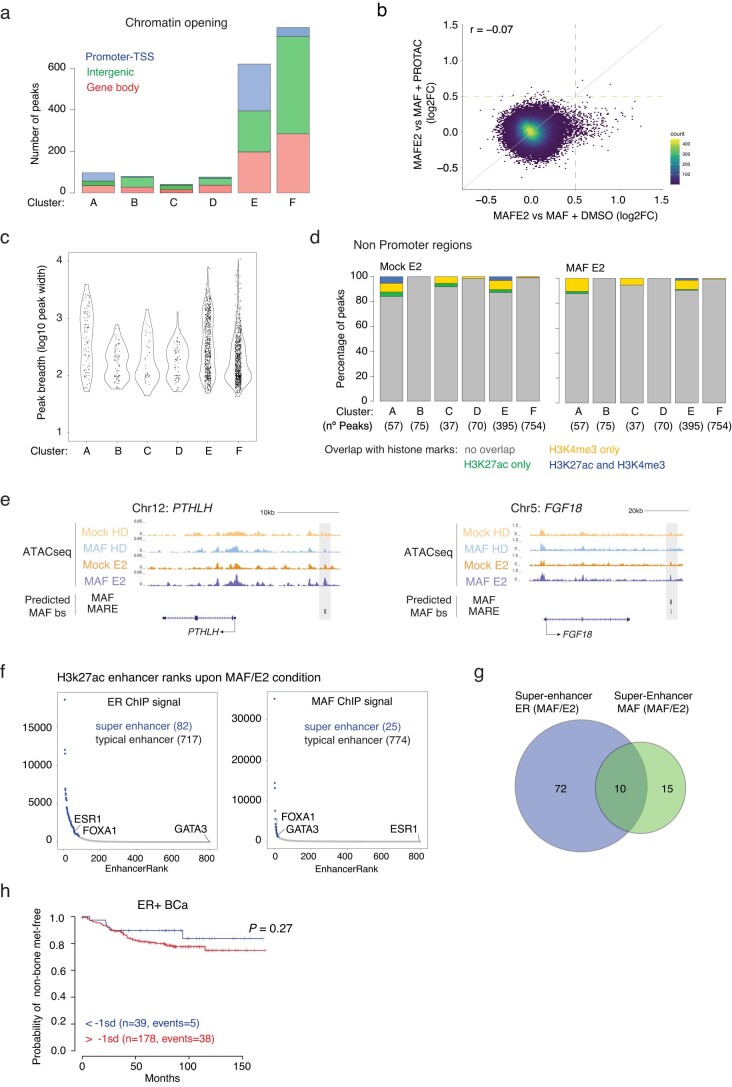
Characterization of ER and MAF impact on chromatin accessibility landscape. **a**, Total number of ATAC peaks in the different cluster groups from **5a** according to promoter-TSS / gene body / intergenic annotations. **b**, Scatter plot reporting the correlation between PROTAC and DMSO conditions in MAF-E2 ATACseq peaks. **c**, Peak breadth plot of ATAC-seq peaks of clusters from **5a. d**, Plot depicting the percentage of ATAC-seq peaks from non-promoter regions corresponding to clusters described in **4a** that overlap with H3K27ac (active enhancers and promoters), H3K4me3 (active promoters) or both (active promoters – broad peaks). Statistical significance, one-sided permutation test. **e**, UCSC genome browser (http://genome.ucsc.edu, Feb. 2009 (GRCh37/hg19)) screenshots of ATAC-seq profiles of *PTHLH* (left) and *FGF18* (right) target genes. ATAC-seq tracks from control and MAF-overexpressing MCF7 cells under HD conditions and after E2 stimulation are shown. Predicted MAF binding sites (using the MAF or MARE matrices) are represented in black. **f**, Enhancers were ranked based on increasing ER (left) and MAF (right) signal. Genes within super-enhancers in both conditions cells are highlighted. **g**, Venn diagram showing overlap of defined super-enhancers occupied by ER (in MAF/E2 conditions) and MAF. **h**, Kaplan Meier curves representing the probability of non-bone metastasis-free survival in ER+ BCa patients (MSKCC-EMC data set; Gawrzak et al., 2018) stratified according to the expression of a MAF-dependent gene signature generated from the integration of RNA-seq transcriptomic and MAF- and MAF/E2-dependent ER ChIP-seq data. Long-rank test, two tailed.

We next generated chromatin accessibility maps of highly metastatic, MAF-positive MCF7 BCa cells upon E2 stimulation (Extended Data Fig. 1c). Both MAF overexpression and E2 stimulation left distinct footprints on cells’ chromatin state compared to control cells, as measured by changes in chromatin accessibility; we could define MAF-dependent clusters A and B (97 and 80 peaks, respectively), E2-dependent clusters C and D (40 and 76 peaks, respectively) and MAF/E2-dependent clusters E and F (621 and 797 peaks, respectively) (Fig. 5a–c). The widespread chromatin remodelling specific for MAF/E2-dependent clusters was lost upon ER depletion (Extended Data Fig. 8a,b), predominantly at annotated promoters/TSSs (Fig. 5d and Extended Data Fig. 8a). Importantly, the breadth of ATAC (assay for transposase-accessible chromatin) peaks was significantly increased in promoter/TSS regions of the MAF-dependent clusters A and E as compared to the remaining clusters (Fig. 5e and Extended Data Fig. 8c). Notably, the MAF-dependent ATAC peaks overlapped with histone marks in BCa cells and associated with activation of transcription (H3K27Ac, H3K4me3) in promoter regions (Fig. 5f and Extended Data Fig. 8d). As MAF itself binds gene bodies as well as intergenic regions (Extended Data Fig. 7c), we postulate that MAF binding at (super)enhancers leads to the activation of broad epigenetic domains^41^, thus changing cell lineage characteristics and potentially the metastatic capacity of ER^+^ BCa cells. Indeed, a subset of AP1 TF binding motifs were enriched in the MAF/E2-dependent open chromatin regions (Extended Data Fig. 8e and Supplementary Table 4). We next analysed data from genome-wide H3K27Ac ChIP-seq upon ER (MAF/E2 condition) or MAF ChIP-seq signal peaks in MCF7 cells with the ROSE algorithm^42,43^ to find super-enhancers characterized by extensive H3K27Ac over ER or MAF common peaks (Extended Data Fig. 8f). Overall, we defined 82 and 25 super-enhancers occupied by ER or MAF, respectively (Extended Data Fig. 8g).

To determine whether these identified alterations in chromatin accessibility peaks reflect the observed transcriptional changes (Fig. 3a), we integrated ATAC-seq and RNA-seq datasets. ATAC-seq data from MAF-overexpressing E2-treated cells were significantly enriched in genes whose expression was regulated by both MAF and E2 (Fig. 5g; clusters A to F, as defined in Fig. 5a).

### MAF and ER regulate a metastasis gene expression program

**Fig. 6:**
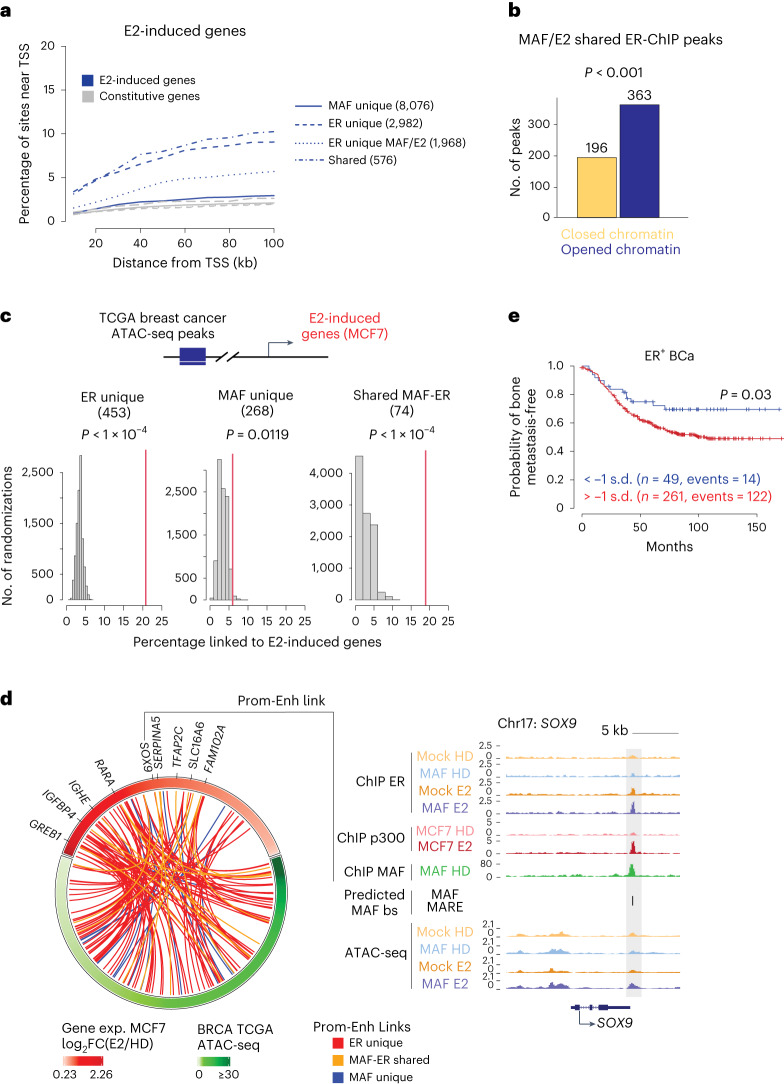
Shared ER-MAF binding sites control the E2-induced metastasis gene program. **a**, Enrichment of chromatin binding sites from MCF7 cells in the vicinity of E2-induced genes compared with an equal number of constitutively expressed genes in MCF7 cells. Lines illustrate the cumulative percentage of sites within a given distance from the TSSs. **b**, MAF/E2-shared ER-ChIP peaks that overlap with ATAC-seq peaks are enriched in the MAF/E2 condition (MAF/E2 versus mock comparison in ATAC-seq data; *P* < 0.10). Only one peak per gene is considered. Permutation test, two-tailed. **c**, Percentage of BCa ATAC-seq peaks (from TCGA^44^) occupied in MCF7 cells by both MAF and ER, or only MAF or ER, that are connected to E2 target gene promoters through promoter–enhancer connections (red line). The distribution of connections between the same number of random ATAC-seq peaks and E2 target gene promoters is shown for comparison (grey); 10,000 iterations were performed to estimate the distribution to test the null hypothesis. Permutation test, one-tailed. **d**, Identified functional promoter–enhancer links between E2-induced genes and MAF/ER-, MAF- and ER-occupied BCa ATAC-seq peaks. **e**, Kaplan–Meier curves representing the probability of bone metastasis-free survival in ER^+^ BCa patients (MSKCC-EMC dataset^13^), stratified according to the expression of a MAF-dependent gene signature generated from the integration of RNA-seq transcriptomics and MAF- and MAF/E2-dependent ER ChIP-seq data. Log-rank test, two-tailed.

To link these chromatin binding events to transcriptional regulation, we further characterized the MAF/E2-dependent genes, using the previously described RNA-seq data (Fig. 3a). GO analysis showed that some genes upregulated by E2 are known oestrogen-response genes (for example, *MYC* and cell-cycle mediators). Other MAF/E2-dependent genes belong to pathways not previously linked to E2 signalling (Fig. 3b,d), including genes linked to an aggressive and highly metastatic BCa phenotype (for example, EMT, inflammation). Importantly, chromatin-binding sites shared between MAF and ER were the most enriched sites adjacent to the recently uncovered E2-targeted genes, along with the classical E2 responses (Fig. 6a). Similarly, differentially open and closed chromatin regions were enriched in ER ChIP peaks (363 peaks in open versus 193 peaks in closed) in the MAF/E2 conditions (Fig. 6b). Collectively, these data suggest that previously unappreciated *cis*-regulatory elements near the herein uncovered E2 target genes are likely to be used by MAF to support ERα binding and activate transcription.

Based on the correlation of the RNA-seq and ATAC-seq signals within 500 kb across multiple TCGA patient tumours^44^, we could infer target genes directly controlled by MAF–ER binding in patient samples (without relying solely on gene proximity). Importantly, we found that ATAC-seq peaks in patient breast tumours that are occupied by both ER and MAF in MCF7 cells are functionally connected to control of the E2 target gene expression through promoter–enhancer connections, to a higher degree than expected by chance (for example, *SOX9* versus an approximate random permutation, *P* < 0.0001; Fig. 6c,d). In contrast, sites bound by MAF alone did not show this substantial enrichment (Fig. 6c). Collectively, these results indicate that E2 largely signals through ER sites, yet, upon MAF expression, ER is able to bind to previously unappreciated genomic sites together with MAF, thus expanding the E2-induced transcriptional repertoire from the classical E2-responsive gene program. Indeed, a MAF-dependent gene signature generated by integrating RNA-seq transcriptomic and MAF- and MAF/E2-dependent ER ChIP-seq data identified a subset of ER^+^ patients with higher likelihood to initially experience skeletal relapse (Fig. 6e and Extended Data Fig. 8h).

### An epigenetic switch defining ER BCa metastasis

**Fig. 7:**
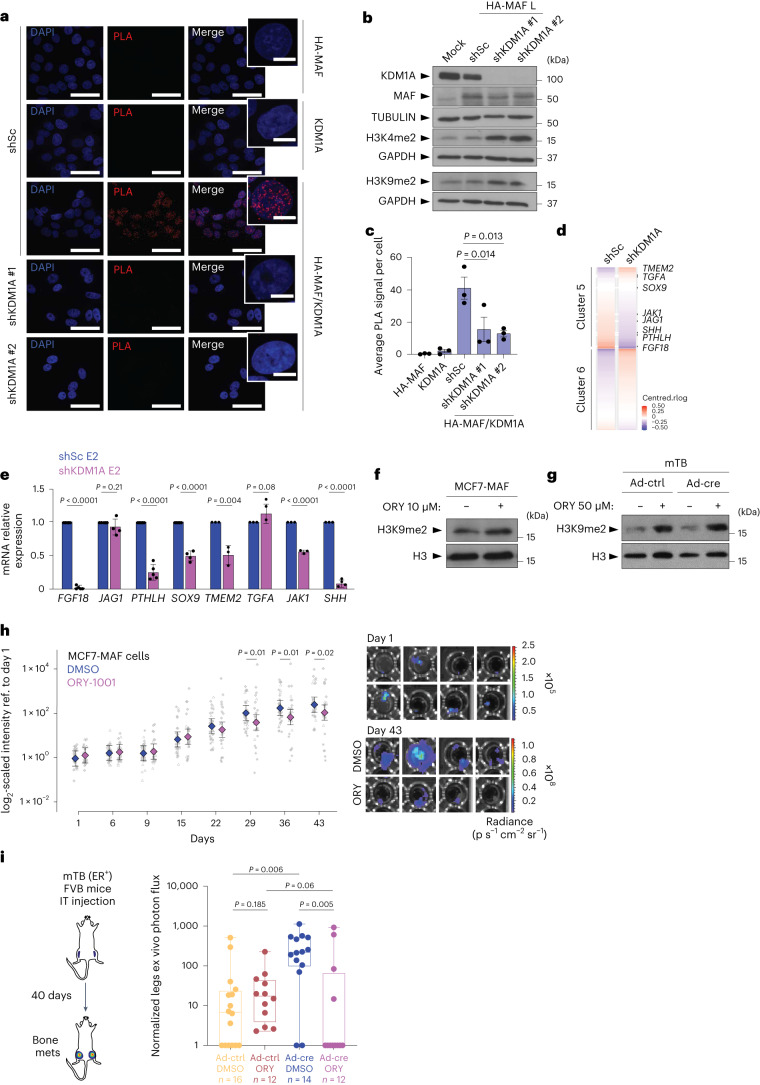
KDM1A inhibition disrupts E2/ER and MAF-dependent signalling and prevents metastasis. **a**, PLA of HA, KDM1A or HA plus KDM1A antibodies together in HA-MAF-L-overexpressing MCF7 cells transduced with scrambled (shSc) or KDM1A-targeted shorthairpin RNA (shRNAs). Representative confocal microscopy images staining, with zoomed insets, are shown. Scale bars, 50 μm. Inset scale bars, 10 μm. **b**, Representative immunoblot showing MAF and KDM1A expression in control and *MAF*-overexpressing MCF7 cells with or without *KDM1A* knockdown. Tubulin and GAPDH, loading controls. **c**, PLA signal quantification. Each dot represents the average PLA signal of 140 to 252 nuclei/condition/biological replicates (*n* = 3 biological replicates). Bars indicate mean ± s.e.m. Statistical significance, two-tailed Wilcoxon rank-sum test. **d**, RNA-seq heatmap showing expression profiles of genes from clusters 5 and 6 in *MAF*-overexpressing cells treated with E2 and with or without *KDM1A* knockdown. **e**, qRT–PCR expression analysis of selected genes in cluster 5 in *MAF*-overexpressing MCF7 cells treated with E2 and with or without *KDM1A* knockdown. Expression was normalized to *GAPDH*. *FGF18, n* = 5; *JAG1*, *n* = 6; *PTHLH*, *n* = 5; *SOX9*, *n* = 4; *TMEM2*, *n* = 3; *TGFA, n* = 3; *JAK1, n* = 3 and *SHH, n* = 4 biological replicates per gene. Data are presented as mean ± s.d. Statistical significance, two-sided *t*-test. **f**,**g**, Immunoblot showing methylation of H3K9 after ORY-1001 treatment in *MAF*-overexpressing or control MCF7 cells (**f**) or mTB cells (**g**). Total H3, loading control. **h**, BICA after ORY-1001 treatment of *MAF*-overexpressing MCF7 bone lesions. Each dot represents an independent bone fragment; *n* = 24 to 30 bone fragments (from three different mice) in each group. Representative bioluminescence images show MAF-overexpressing MCF7 cells treated with DMSO or ORY-1001 at day 1 or day 43. Data are presented as mean ± s.e. Linear mixed model fit by REML with *z*-tests for individual comparisons, two-tailed. Significance was defined by the adjusted *P* value using the Benjamini and Hochberg multiple testing correction. **i**, Schematic diagram of bone colonization (left) and normalized ex vivo bone metastasis photon flux quantification (right), in which mice with intratibial (IT) injections of control or *MAF*-overexpressing mTB cells were treated with DMSO (Ad-ctrl, *n* = 16 limbs; Ad-cre, *n* = 14 limbs) or ORY-1001 (Ad-ctrl, *n* = 12 limbs; Ad-cre, *n* = 12 limbs) for 40 days. The median (centre line), first and third quartiles (box limits) and the minimum to maximum values (whiskers) are shown. Statistical significance, two-tailed Mann–Whitney test. Source data

**Extended Data Fig. 9:**
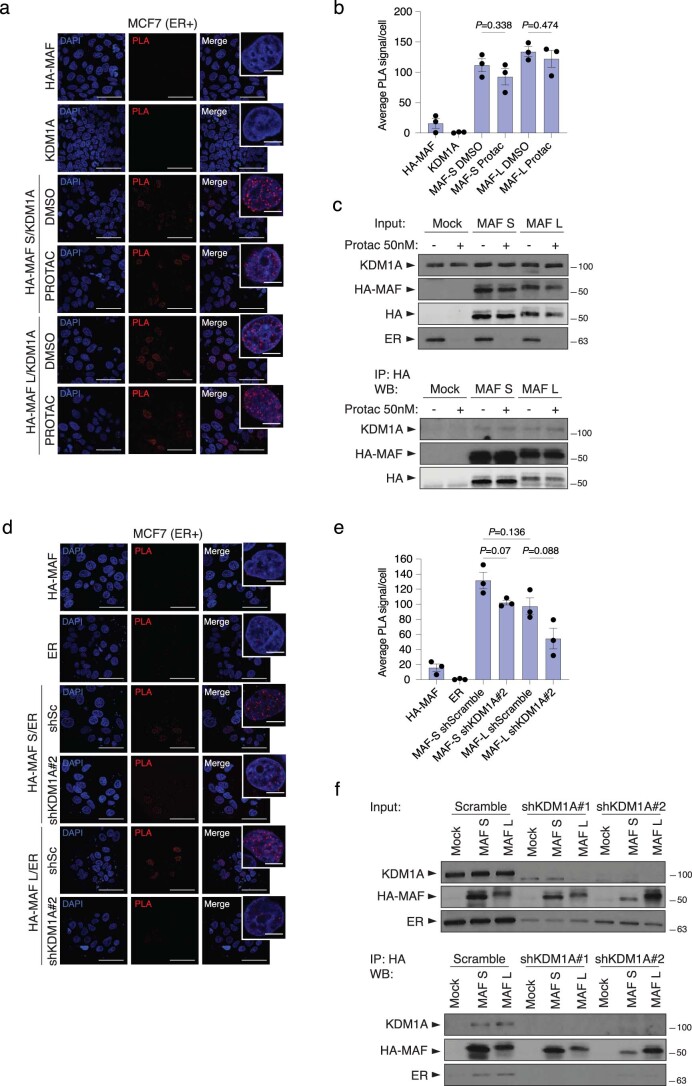
ER and MAF interaction dependency on KDM1A. **a**, Proximity ligation assay (PLA) of HA or KDM1A antibody alone or both antibodies together in MAF-overexpressing MCF7 cells treated with DMSO or ER-PROTAC (50 nM) 24 h prior to fixation. Representative confocal microscopy images for PLA red signal and DAPI nuclear staining with magnified inset are shown. Scale bar, 50 μm; inset scale bar, 10 μm. **b**, PLA signal quantification. Each dot represents the average PLA signal from 50 to 142 nuclei per condition. *n* = 3 biological replicates. Bars represent mean ± sem. Statistical significance, two-tailed Wilcoxon n-test. **c**, Representative immunoblots showing HA co-IP of HA-tagged MAF (S or L isoforms) with endogenous KDM1A upon ER degradation with a PROTAC. **d**, PLA of HA or ER antibody alone or both antibodies together in MAF-overexpressing MCF7 cells transduced with scrambled (shSc) or KDM1A-targeted shRNA. Representative confocal microscopy images for PLA red signal and DAPI nuclear staining with magnified inset are shown. Scale bar, 50 μm; inset scale bar, 10 μm. **e**, PLA quantification. Each dot represents the average PLA signal from 53 to 126 nuclei per condition. *n* = 3 biological replicates. Bars represent mean ± sem. Statistical significance, two-tailed Wilcoxon rank-sum test. **f**, Representative immunoblots showing HA co-IP of HA-tagged MAF (S or L isoforms) with endogenous ER in cells transduced with scrambled or *KDM1A*-targeted shRNAs. Source data

We next focused on high-confidence MAF interactors that could be connected to the MAF-mediated global chromatin opening via histone methylation and that have previously been linked to cancer. KDM1A, which gave a significance analysis in the interactome of Bayesian false discovery rate (BFDR) < 0.001 (Fig. 2c), encodes a flavin-dependent monoamine oxidase that demethylates mono- and dimethylated lysines (K), and specifically histone 3 and lysines 4 and 9 (H3K4 and H3K9). Notably, high KDM1A activity is present in many cancer types, including BCa^45,46^. To determine the effects of the inhibition of KDM1A activity on E2-mediated transcriptional rewiring in the context of MAF overexpression, we performed PLA assays and co-IP of KDM1A and MAF in MCF7 BCa cells (Fig. 7a and Extended Data Fig. 3d). We confirmed that KDM1A and MAF are within interacting distance and can directly interact, independently of ER (Extended Data Fig. 9a–c). Cells with a *KDM1A* knockdown had a significantly reduced PLA signal as compared to control cells (with shorthairpin scramble (shSc)) (Fig. 7a–c). Strikingly, upon KDM1A depletion, we observed a reduced interaction between MAF and ER (Extended Data Fig. 9d–f), even considering ER expression diminution in KDM1A-depleted cells. These results confirm that MAF directly binds to KDM1A and suggests that this interaction helps to facilitate the MAF–ER interaction.

**Extended Data Fig. 10:**
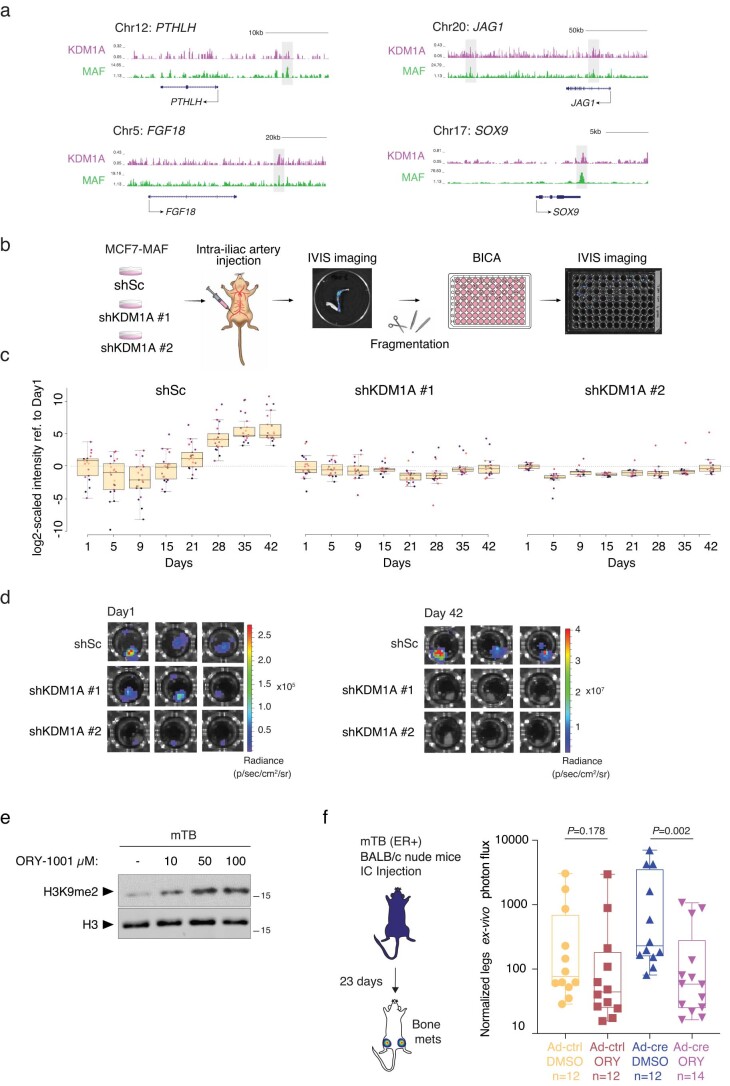
KDM1A supports MAF-dependent metastatic functions. **a**, UCSC genome browser (http://genome.ucsc.edu, Feb. 2009 (GRCh37/hg19)) screenshot of ChIP-seq profiles of the *PTHLH, JAG1, FGF18* and *SOX9* target genes. MAF ChIP-seq tracks from *MAF*-overexpressing MCF7 cells under HD conditions are shown. KDM1A ChIP-seq peaks (published data, Sheng et al., 2018) in MCF7 cells are shown. **b**, Schematic representation of BICA experimental design. Luciferase-tagged control (scramble shRNA, shSc) and KDM1A-knockdown MCF7 cells with MAF overexpression were inoculated into mice through intra-iliac artery injection. Femur and tibia bones were extracted, segmented into pieces and arranged into 96-well plates filled with cell culture media. Cancer cell growth was traced weekly using bioluminescence imaging (IVIS imaging system). **c**, BICA assay showing the reduction of KDM1A-knockdown cell growth compared to control MAF-overexpressing cells. Each dot represents an independent bone fragment. *n* = 17 to 21 bone pieces (from 3 different mice) for each condition. The median (centre line), first and third quartiles (box limits) and the minimum to maximum values (whiskers) are shown (middle). **d**, Representative bioluminescence images of bone fragments containing control (shSc) and KDM1A-knockdown MCF7 cells with MAF overexpression at day 1 and at day 42. **e**, Immunoblot showing methylation of H3K9 (as a surrogate for KDM1A activity) in mouse BCa cells after treatment with different concentrations of ORY-1001. Total histone H3, loading control. **f**, Schematic representation of the experimental design. Normalized *ex vivo* photon flux quantification of bone metastasis in BALB/c nude mice injected intracardially with control (Ad-ctrl) and MAF-overexpressing (Ad-cre) mouse BCa cells and treated with DMSO (Ad-ctrl, *n* = 12 limbs; Ad-cre, *n* = 12 limbs) or ORY-1001 (Ad-ctrl, *n* = 12 limbs; Ad-cre, *n* = 14 limbs) for 23 days. The median (centre line), first and third quartiles (box limits) and the minimum to maximum values (whiskers) are shown (middle). Statistical significance, two-tailed Mann–Whitney test. Source data

To analyse whether a *KDM1A* knockdown affects MAF/E2-mediated gene responses, we compared RNA-seq data from HD versus E2-treated cells (clusters 5 and 6 from Fig. 3a) (Fig. 7d). In *MAF*-overexpressing cells, we observed that the lack of KDM1A activity reduced the expression of genes that were E2/ER-induced and MAF-dependent (for example, *FGF18*, *PTHLH*, *SOX9*, *TMEM2*, *JAK1* and *SHH*) (Fig. 7d,e), but not of other genes (Fig. 7d,e). Furthermore, MAF ChIP-seq peaks in *PTHLH*, *JAG1*, *FGF18* and *SOX9* overlapped with KDM1A ChIP-seq peaks previously defined in MCF7 BCa cells^47^ (Extended Data Fig. 10a). Taken together, these results indicate that KDM1A contributes to MAF-dependent gene responses, including a subset of MAF/E2-dependent gene responses.

### KDM1A blockade antagonizes MAF-dependent metastasis

We next tested whether iadademstat (ORY-1001), a highly selective covalent inhibitor of KDM1A^48^, could block MAF-mediated metastasis. After ORY-1001 treatment, MCF7 and mTB cells showed H3K9me2 accumulation, demonstrating functional inhibition of KDM1A demethylase activity (Fig. 7f,g). To test whether KDM1A inhibition blocks MAF-expressing BCa cells from forming bone metastasis, we used an in vitro bone-in-culture array (BICA)^49^. Intra-iliac BCa cell injection was followed by bone fragmentation and growth in culture (Extended Data Fig. 10b). We observed a significant inhibition of MAF-driven BCa growth in bone after ORY-1001 treatment (Fig. 7h) and of bone colonization after *KDM1A* knockdown (Extended Data Fig. 10c,d). We then inoculated control or *Maf*-expressing mTB cells into the tibia of immunocompetent syngeneic mice (FVB background). Notably, treatment with ORY-1001 showed a significant reduction of both the number and size of bone lesions but only in the *Maf*-expressing group (Fig. 7i and Extended Data Fig. 10e), suggesting that KDM1A-mediated epigenetic changes in *Maf*-expressing cells drive adaptation to bone and metastasis. These results were confirmed by intracardiac injection of mTB cells into BALB/c nude mice (Extended Data Fig. 10e,f).

## Discussion

Here we show that an increased level of one TF (MAF) alters the topography of another (ER) by fine-tuning the enhancers, which in turn alters tissue development, homeostasis and pathology^50^. Transcriptional reprogramming is a common feature of cancer, in which aberrant oncogene or tumour suppressor silencing enables normal cells to undergo malignant transformation. This can be due to copy number changes, chromosomal rearrangement, somatic mutations of protein-coding genes and/or changes in *cis*-elements in non-coding genomic regions^15^. We report a previously unknown MAF–ER interaction that is driven by an epigenomic mechanism and that contributes to a clinically relevant metastasis outcome in BCa. This involves a MAF-driven chromatin perturbation that substantially expands the ER transcriptional reach beyond those targets currently described. These targets include *PTHLH*, *JAG1*, *FGF18* and *SOX9*, which are known to accelerate and support metastatic dissemination, initiation and colonization functions by facilitating phenotypic adaptation to unknown settings and conditions^51^. *PTHLH* and *JAG1* are two well-established mediators of bone–tumour interactions (PTHLH for endochondral bone formation and Ca^2+^ homeostasis through bone remodelling^26,27^, and JAG1 for bone metastasis via Notch signalling in osteoblasts)^29^. FGF18 acts as a mitogenic stimulus and has been related to metastasis^30^. Finally, SOX9 is a stem cell mediator with a critical role in breast epithelial lineage commitment^52^. Collectively, our findings support enhanced bone metastasis in ER^+^ BCa following MAF-amplification in the presence of E2.

Differentiation in luminal tumours is sustained by a cooperative network between the TFs ER, FOXA1 and GATA3, but little is known about how this is regulated during metastasis progression or treatment resistance, aside from some specific mutations in TFs (for example, SY242CS BCa mutation in FOXA1) or chromatin remodellers (for example, histone methyltransferases and histone demethylases)^16,39,53–55^. Here we show that the combined activity of MAF and ER interplays with the above network and the cancer-cell phenotypic plasticity landscape^56^. Importantly, we demonstrate that *MAF* amplification and overexpression in ER^+^ tumours reprogram the canonical cistrome to include previously unknown enhancer and transcriptional activity, thereby enhancing metastasis to bone. This program redirects DNA accessibility and channels KDM1A-mediated transcription to promote metastasis. These data imply that chromatin dysregulation is an early event in BCa metastasis, and that genomic amplifications play a central role in this process.

In BCa, *MAF* amplification can predict metastasis and poor response to adjuvant bisphosphonates^10,12^. Whereas bone metastasis can be managed clinically for years, adjuvant bone-modifying treatments used for patients with *MAF*-amplified BCa tumours are associated with extensive multi-organ metastasis and poor overall survival^10,12^. Recent studies have uncoupled the actions of bone-modifying agents from cancer-cell proliferation in different cancer types^57^. Thus, treatments targeting the metastatic stroma can potentially displace metastasis to other sites, compromising overall survival. MAF supports skeletal metastasis and explains the substantial increase in extraskeletal metastasis observed in MAF-amplified BCa, younger, pre-menopausal patients who have been treated with a bone-modifying bisphosphonate to prevent bone metastasis^10,12^. Importantly, our results open the door to exploring KDM1A inhibition in this clinical context.

## Methods

### Animal studies

All animal work was approved by the Ethical Committee of Animal Experimentation of the Government of Catalonia (protocols 10508-P1 and 9096-P1). Female, 12-week-old mice were used (BALB/c nude for studies involving MCF7 and mBCa cells, and FVB/NJ for studies with mBCa cells) (Harlan). Mice were maintained in a specific-pathogen-free (SPF) facility under a 12-h/12-h light–dark cycle, under controlled temperature and humidity (18–23 °C and 40–60%, respectively) and with ad libitum access to standard diet and water. All of the mice were closely monitored by the authors, facility technicians and by an external veterinary scientist responsible for animal welfare. We monitored tumour growth using intravital bioluminescence at least once a week. Mice that died or were euthanized for ethical reasons before experimental endpoints were excluded. Oestrogen (320 ng µl^−1^) was supplemented in drinking water to sustain the growth of ER^+^ cell tumours.

Mice were anaesthetized with ketamine (100 mg per kg body weight) and xylazine (10 mg per kg body weight). Cells (3 × 10^5^ for MCF7, or 5 × 10^5^ for mBCa) were resuspended in 100 µl of phosphate-buffered saline (PBS) and injected into the left cardiac ventricle of mice using a 26-G needle. Zoledronic acid (ZOL; Merck) was initiated when bone lesions were established (mice were treated once a week with 500 µg per kg body weight).

For orthotopic transplant experiments, mice were anaesthetized as described above, then 1 × 10^6^ cells were resuspended in 1:1 Matrigel growth factor reduced and PBS, and injected into the fourth mammary fat pads. When tumours reached 300 mm^3^, mice were euthanized, and blood was extracted for circulating tumour cells analysis (MCF7 cells) or tissues were imaged for metastasis detection (mTB cells).

Immediately after injection, mice were imaged for luciferase activity using the IVIS SpectrumCT imaging system from Xenogen (Living Image 2.60.1 software) to confirm successful xenograft. Bone metastasis development was followed weekly by bioluminescence imaging using the IVIS SpectrumCT platform, and data were recorded using Living Image software. To measure bone colonization, the photon flux was calculated for each mouse using two circular regions of interest (ROIs) in a hind leg. After subtracting the background value, the photon flux was normalized to the value obtained at xenografting. Metastatic colonization was defined as a photon flux value greater than the bioluminescence signal at day 0. For ex vivo imaging, mice were injected with luciferin before euthanasia. Legs and other organs were tested for metastatic growth by bioluminescence at 5-min post-injection.

For KDM1A inhibition in vivo, control and *MAF*-overexpressing mBCa cells were pre-treated with 50 µM ORY-1001 (Selleckchem) for 96 h before injection. A cell solution (2 × 10^4^) resuspended in 5 µl of PBS was injected into the upper half of the tibia medullary cavity using a 28-G needle in female FVB/NJ mice. Inoculation was confirmed by bioluminescence, and the development of bone lesions was followed by weekly imaging. In vivo ORY-1001 treatment was initiated at one-day post-implantation of cells; mice were treated five days per week by intraperitoneal injection with 0.03 mg per kg body weight. Control groups were treated with dimethylsulfoxide (DMSO).

Experiments were terminated when tumour bioluminescence measurements reached saturation or if the animals presented signs of suffering or poor health status according to protocols approved by the animal experimentation committee of the Barcelona Science Park.

Before injection, MCF7 and mBCa-control cell lines were stably transduced with TK-GFP-luciferase construct and sorted for green fluorescent protein (GFP).

### Generation of *Maf* transgenic mice

Mouse *Maf* complementary DNA (cDNA) was synthesized downstream of a Kozak sequence cloned into a pOC1 plasmid (GeneScript). The *CAGGS* promoter followed by the *lox-STOP-lox* sequence was obtained from the CAGGS-lox-STOP-lox-eGFP plasmid and inserted upstream of the mouse *Maf* cDNA with NheI and KpnI restriction sites. Alternatively, an *IRES* sequence was synthesized within the pOC2 plasmid (GeneScript). *fLuc* fused with e*GFP* from the TGL plasmid^60^ was inserted downstream of the *IRES* sequence by NcoI and BamHI in the pOC2 plasmid. Next, both constructs were fused to obtain the Maf-IRES-eGFP/fLuc (MGL) cassette. Finally, the MGL cassette was cloned into PacI and AscI sites of the pROSA26-PA plasmid. The linearized final vector was electroporated into G4 mouse embryonic stem cells (mESCs) derived from C57BL6/129Sv/F1 mice. Three 96-well plates were picked, and one plate was analysed. Five positive clones were identified for both arms by long-range polymerase chain reaction (PCR). Homologous recombination was verified by Southern blot. One positive clone was injected into C57BL/6 blastocysts to generate chimeras that transmitted the transgene to their offspring. The resulting founders were genotyped by long-range PCR of tail genomic DNA samples using primers located in the *Rosa26* locus of the mouse genome before and after the cloning site together with primers located in the CAGGS promoter and fLuc sequence of the cassette. Transgene insertion was also confirmed by Southern blot using a probe located in the STOP cassette of the transgene to avoid clones with double integrations. The strain was genotyped by Transnetyx.

### Southern blot analysis

Genomic DNA was isolated using standard procedures. To assess homologous recombination of the targeting vector, 5 µg of DNA was digested overnight with the appropriate restriction enzyme and separated on a 0.8% agarose gel overnight at 15 V. DNA fragments were then transferred to a Hybond-N+ membrane (Amersham) overnight by capillarity of 0.4 N NaOH. The next day, the membrane was pre-hybridized with a hybridization solution for 1 h at 42 °C, and the membrane was hybridized overnight at 43 °C with the specific digoxigenin (DIG)-labelled probe. Probes were generated by PCR with specific primers and labelled with DIG-11-dUTP nucleotides (Roche). Membranes were incubated the following day with an anti-DIG-AP antibody for 30 min. Probes were detected using a CDP Star solution and by exposure in an Amersham Hyperfilm ECL membrane (GE Healthcare). Primer sequences for amplification of the 5′ and 3′ probes are shown in Supplementary Table 5.

### Long PCR analysis

Genomic DNA was isolated using standard procedures. Long PCRs to validate the integration of the targeting vector were performed with a GoTaq Long PCR kit (Promega) following the manufacturer’s instructions. For 5′ integration, a forward primer (R26F) upstream of the 5′ homology arm and a reverse primer (CMVR) at the beginning of the CAG promoter were used. Similarly, for the 3′ integration PCR, a forward primer (LucF) at the luciferase sequence and a reverse primer (R26R) downstream of the 3′ homology arm were used. Primer sequences are shown in Supplementary Table 5.

### Cell culture

MCF7 (ref. HTB-22), T47D (ref. HTB-133), MDA-MB-231 (ref. HTB-26) and HEK 293T (ref. CRL-1573) human BCa cell lines were purchased from the American Type Culture Collection (ATCC). All cell lines were maintained in standard conditions (37 °C, 5% CO_2_) in Dulbecco’s modified Eagle medium (DMEM, Gibco) supplemented with 10% fetal bovine serum (FBS), 0.29 mg ml^−1^ glutamine, 100 units ml^−1^ penicillin and 0.1 mg ml^−1^ streptomycin (Biological Industries). Cells were split every two to three days and routinely tested for mycoplasma infection.

To evaluate E2 stimulation, MCF7, T47D and mTB BCa cells were maintained in HD medium (phenol-red free DMEM (Gibco) supplemented with 5% charcoal-stripped FBS (Capricorn Scientific), glutamine 0.29 mg ml^−1^, penicillin 100 units ml^−1^ and streptomycin 0.1 mg ml^−1^) for 72 h before addition of E2 (10 nM, Sigma-Aldrich) or ethanol (vehicle).

### Mouse BCa-induced tumours and derived cell explants

For the induction of mammary tumours, Rosa26^LSLMaf^ female, seven-week-old mice were subcutaneously injected with 15 mg MPA (Depo-Provera). One milligram of DMBA was administered weekly by oral gavage during the following four weeks. Tumours were detected and monitored by manual palpation.

To generate mouse BCa explants, freshly isolated tumours were minced with sterile razor blades and digested for 30 min at 37 °C with 3 mg ml^−1^ collagenase A (Roche), 0.1% trypsin and fungizone in serum-free DMEM. Digestion was stopped by the addition of DMEM with 10% FBS, and the suspensions were dispersed through a 100-μm cell strainer. Cells were initially cultured in DMEM–10%FBS supplemented with 5 µg ml^−1^ insulin, 5 ng ml^−1^ mouse epidermal growth factor (EGF) and 5 ng ml^−1^ cholera toxin (all from Merck). After establishment, mouse cell lines were cultured in DMEM with FBS, 0.29 mg ml^−1^ glutamine, 100 units ml^−1^ penicillin and 0.1 mg ml^−1^ streptomycin.

### Generation of *MAF*-overexpressing cells

For stable *MAF* overexpression, HA-tagged *MAF* (short and long isoforms) was PCR-amplified using primers containing HpaI restriction sites, and cloned into the retroviral plasmid MSCV-neo (Supplementary Table 5). To produce retroviruses, human embryonic kidney 293T cells (HEK 293T) were seeded at 80% confluence in 150-cm^2^ plates and transfected 16 h later with 12 µg MSCV-Mock or MSCV-MAF, 1.2 µg VSVG-R and 10.8 µg GAG-POL plasmids using polyethylenimine (PEI) in 150 mM NaCl solution. The viral supernatant was collected 72 h after transfection, passed through a 0.45-µm cellulose acetate filter (Whatman) and used to infect MCF7, T47D and MDA-MB-231 cells. Specifically, 3 × 10^5^ cells were seeded into a six-well plate followed by the addition of viral medium with 8 µg µl^−1^ polybrene (Sigma-Aldrich) and overnight incubation. Viruses were removed, and medium was added for cell recovery. Selection was performed 24 h after recovery with 1 mg ml^−1^ neomycin (G418; Santa Cruz Biotechnology) for 10 days.

For the expression of full-length and truncated MAF proteins, short, long and truncated MAF sequences were PCR-amplified using primers with EcoRI and BamHI restriction sites incorporated, and cloned downstream of an N-terminal 2xHA tag of a pCMV5 plasmid (Supplementary Table 5). Truncated MAF sequences included MAF L (ΔN-t 1), encoding MAF amino acids 85–404 and lacking part of the transactivation domain, and MAF L (ΔN-t 2), encoding MAF amino acids 120–404 and lacking the entire transactivation domain. MCF7 cells were transfected with the generated plasmids using GenJet DNA transfection reagent (SignaGen Laboratories) according to the manufacturer’s protocol.

In mouse BCa-derived cells, cell explants were infected with Ad-mCherry (control) and AdCre-mCherry particles (multiplicity of infection of 100). After five days, mCherry^+^ cells (control) and mCherry^+^/GFP^+^ double-positive cells (*MAF*-overexpressing cells) were sorted and seeded for expansion.

### Lentiviral production

HEK 293T cells were seeded at 80% confluence in 150-cm^2^ plates and transfected 16 h later with 6 µg of lentiviral vectors expressing shRNA against human *KDM1A*, *AR*, *PTHLH*, *JAG1* and mouse *Ar* (Sigma Mission shRNA Library), 6 µg pMDLg/pRRE, 6 µg pRSV-REV and 6 µg pMD2.G/VSVG using polyethylenimine (PEI) in 150 mM NaCl solution. The viral supernatant was collected 72 h after transfection, passed through a 0.45-µm cellulose acetate filter (Whatman) and used to infect BCa cells. Specifically, 3 × 10^5^ cells were seeded into a six-well plate followed by the addition of viral medium with 8 µg µl^−1^ polybrene (Sigma-Aldrich), and overnight incubation. Viruses were removed and medium was added for cell recovery. Selection was performed 24 h after recovery with 2 µg ml^−1^ puromycin for 72 h. Short hairpin sequences are provided in Supplementary Table 5.

### Proximity-dependent biotin identification (BioID)

Short and long MAF isoforms were PCR-amplified using primers containing EcoRI and KpnI (N-terminal tag) or NheI and HpaI (C-terminal tag) restriction enzyme sites (Supplementary Table 5) and cloned into myc-BioID2-MCS (Addgene plasmid 74223, https://www.addgene.org/74223/) or MCS-BioID2-HA (Addgene plasmid 74224, https://www.addgene.org/74224/).

MCF7 or MDA-MB-231 cells were transfected with control (empty myc-BioID2) or MAF plasmids using GenJet DNA transfection reagent (SignaGen Laboratories) according to the manufacturer’s instructions. Five 15-cm plates per condition were grown to 70% confluence before treatment with biotin (50 μM) for 24 h. MCF7 samples were performed with two biological replicates and MDA-MB-231 cells with one biological replicate. Trypsinized cell pellets were washed with ice-cold PBS and lysed in 5 ml of lysis buffer (50 mM Tris-HCl pH 8, 150 mM NaCl, 0.1% SDS, 2 mM MgCl_2_, 1% Triton X-100, 1 mM EDTA, 1 mM EGTA, 1 mM NaF, 1 mM Na_3_VO_4_, protease inhibitor cocktail (Roche)) with 1:2,000 benzonase (Sigma-Aldrich) by rotating for 1 h at 4 °C. Samples were sonicated 3 × 30 s with Bioruptor (Diagenode) and centrifuged at 16,000*g* for 30 min at 4 °C. Biotinylated proteins were isolated by affinity purification with Dynabeads MyOne Streptavidin C1 beads (Thermo Fisher Scientific) with rotation for 4 h at 4 °C. The beads were washed once with lysis buffer and three times with 50 mM NH_4_HCO_3_, then snap-frozen before tryptic digestion.

Samples were on-bead tryptic-digested at an enzyme concentration of 0.1 μg µl^−1^ in 50 mM NH_4_HCO_3_ at 37 °C overnight. The following morning, an additional 1.08 μg of trypsin was added, followed by incubation for 2 h at 37 °C. To stop the digestion, formic acid was added to a final concentration of 1%. Samples were cleaned up through polyLC C18 tips, and peptides were eluted with 80% acetonitrile/1% TFA. Next, samples were diluted to 20% acetonitrile/0.1% trifluoracetic (TFA), loaded into strong cation exchange columns (SCX), and peptides were eluted in 5% NH_4_OH/30% methanol. Finally, the samples were evaporated to dryness, reconstituted to 50 µl, and diluted 1/8 with 3% acetonitrile/1% formic acid aqueous solution for MS analysis.

The nano-LC-MS/MS set-up was as follows. Digested peptides were diluted in 3% acetonitrile (ACN) and 1% formic acid (FA). The sample was loaded to a 300 μm × 5 mm PepMap100, 5 μm, 100 Å, C18 μ-precolumn (Thermo Scientific) at a flow rate of 15 μl min^−1^ using a Thermo Scientific Dionex Ultimate 3000 chromatographic system (Thermo Scientific). Peptides were separated using a C18 analytical column (NanoEase MZ HSS T3; 75 μm × 250 mm, 1.8 μm, 100 Å; Waters) with a 90-min run, comprising three consecutive steps with linear gradients from 3% to 35% B in 60 min, from 35% to 50% B in 5 min, and from 50% to 85% B in 2 min, followed by isocratic elution at 85% B for 5 min and stabilization to initial conditions (A = 0.1% FA in water, B = 0.1% FA in CH_3_CN). The column outlet was directly connected to an Advion TriVersa NanoMate system (Advion) fitted on an Orbitrap Fusion Lumos Tribrid mass spectrometer (Thermo Scientific). This was operated in a data-dependent acquisition (DDA) mode. Survey MS scans were acquired in the Orbitrap with the resolution (defined at 200 *m*/*z*) set to 120,000. The lock mass was user-defined at 445.12 *m*/*z* in each Orbitrap scan. The top-speed (most intense) ions per scan were fragmented by collision induced dissociation (CID) and detected in the linear ion trap. The ion count target value was 400,000 and 10,000 for the survey scan and for the MS/MS scan, respectively. Target ions already selected for MS/MS were dynamically excluded for 15 s. The spray voltage in the NanoMate source was set to 1.60 kV. The radio frequency (RF) lenses were tuned to 30%. The minimal signal required to trigger the MS to MS/MS switch was set to 5,000. The spectrometer was used in positive polarity mode, and singly-charged state precursors were rejected for fragmentation.

A twin database search was performed with two different software packages—Thermo Proteome Discoverer v2.4.1.15 (PD) and MaxQuant v1.6.14.0 (MQ)—using the Sequest HT and Andromeda search engine nodes for PD and MQ, respectively. The database used was SwissProt Human (released in October 2020), including contaminants and MAF proteins (short and long isoforms). The search was run against targeted and decoy databases to determine the false discovery rate (FDR). The search parameters included trypsin enzyme specificity, allowing for two missed cleavage sites, oxidation in M and acetylation in the protein N terminus as dynamic modifications. The peptide mass tolerance was 10 ppm and the MS/MS tolerance was 0.6 Da. Peptides were filtered at an FDR of 1% based on the number of hits against the reversed sequence database.

Contaminant identifications were removed and only unique peptides (those that are not shared between different protein groups) were used for the quantitative analysis with SAINTexpress-spc v3.6.1^61^. SAINTexpress compares the prey control spectral counts with the prey test spectral counts for all available replicates. For each available bait and for each available replicate, prey count was the maximum count result between PD and MQ. With this combined dataset, the SAINTexpress algorithm was run with BioID2-MAF samples (N- and C-terminal fusion, MAF-S and MAF-L isoforms) and the corresponding control samples. The algorithm was independently executed for MCF7 (two biological replicates with three technical replicates) and MDA-MB-231 (three technical replicates) samples. For MCF7 samples, an ‘-R3’ parameter was used to select the three replicates with the highest spectral counts. High confidence interactors were defined as those with a Bayesian FDR of ≤0.02 and a fold change of ≥3. Input and output SAINTexpress data are provided in the Supplementary Information (Supplementary Table 6), and MS raw data are available in the PRIDE repository^62^ (ID_PXD035936).

### Network analysis

Protein–protein interaction data were downloaded from the STRING v11 database^63^ with the following settings: meaning of network edges—confidence; active interaction sources—experiments and databases; minimum required interaction score—0.9. Disconnected nodes in the network were hidden and the Markov cluster (MCL) algorithm was used with an inflation parameter of 2.2.

### Dot-plot analysis

The SAINTexpress output files of MAF-BioID2 baits (N- and C-terminal fusion, MAF-S and MAF-L isoforms) or controls were processed through ProHits-viz^64^ with the dot-plot generator tool (default options) for visualization of selected high-confidence proximity interactors.

### GO enrichment analysis for BioID interactors

Statistically enriched GO terms for BioID high-confidence interactors were identified using the standard hypergeometric test. The background population was defined by all MAF interactors that were observed in the BioID proteomics experiment. Significance was defined by the adjusted *P* value using the Benjamini and Hochberg multiple testing correction.

### Immunoblotting

Trypsinized cell pellets were resuspended in lysis buffer (50 mM Tris/HCl pH 7.4, 1% Triton X-100, 140 mM NaCl, 1 mM EDTA, 1 mM EGTA, 0.1% SDS, 1 mM NaF, 1 mM Na_3_VO_4_) containing a protease inhibitor cocktail (Roche), incubated for 30 min on ice and centrifuged at 21,000*g* for 10 min at 4 °C. The supernatant was kept as the protein extract and concentration was determined by a standard Bradford assay (BioRad). Protein lysate (40 µg of per sample) was mixed with sample buffer (45 mM Tris pH 6.8, 10% glycerol, 1% SDS, 52 mM dithiothreitol (DTT) and 1% bromophenol blue) and heated at 95 °C for 5 min. Proteins were separated by sodium dodecyl sulfate polyacrylamide gel electrophoresis (SDS–PAGE) and transferred to polyvinylidene difluoride (PVDF) membranes (Millipore). The membranes were blocked in 5% bovine serum albumin (BSA) in TBS-Tween (0.1%) for 1 h at room temperature, and incubated at 4 °C overnight with primary antibodies (Reporting Summary). Membranes were then washed three times with TBS-Tween (0.1%) and incubated for 1 h at room temperature with horseradish peroxidase (HRP)-conjugated secondary antibodies or HRP-conjugated streptavidin for biotin detection (Reporting Summary). Finally, membranes were washed three times again and developed with ECL western blotting substrate (Pierce) according to the manufacturer’s protocol.

### Immunofluorescence

A total of 1 × 10^5^ cells were seeded onto glass coverslips. The next day, the cells were fixed with formalin for 20 min, permeabilized with 0.5% Triton X-100 in PBS for 10 min, blocked with 1% BSA in PBS for 45 min, and probed with primary antibodies overnight at 4 °C (Reporting Summary). Primary antibodies were detected using Alexa Fluor 488-conjugated secondary antibodies (Invitrogen). Biotinylated proteins were detected using Alexa Fluor 546-conjugated streptavidin (Invitrogen). 4′,6-Diamidino-2-phenylindole (DAPI) for nuclear staining was contained in ProLong Gold antifade mounting solution (Invitrogen).

Confocal images were taken on a Zeiss LSM780 microscope (Zeiss) using a Plan Apochromat ×63/NA 1.4 oil immersion objective, with 405- and 488-nm laser excitation at a pixel resolution of 132 nm. *Z*-stacks were acquired every 500 nm.

### Co-IP

Cells were transfected with pCMV5 plasmids for HA-tagged MAF expression (short and long isoforms) or an empty plasmid control using GenJet DNA transfection reagent (SignaGen Laboratories), according to the manufacturer’s instructions. At 48 h post-transfection, cells were collected in lysis buffer (50 mM Tris-HCl pH 7.4, 100 mM NaCl, 1% Triton X-100, 1 mM EDTA, 1 mM EGTA, 50 mM NaF, 2 mM Na_3_VO_4_, 10 mM β-glycerophosphate) supplemented with a protease inhibitor cocktail (Roche), incubated for 30 min on ice, and centrifuged at 21,000*g* for 10 min at 4 °C. The supernatant was kept as a protein extract and the concentration was determined using a standard Bradford assay (BioRad). The input (20 µg of protein lysate) was mixed with 2× sample buffer (45 mM Tris pH 6.8, 10% glycerol, 1% SDS, 52 mM DTT and 1% bromophenol blue) and heated at 95 °C for 5 min before immunoblot analysis, then 500 µg of the remaining protein lysate was incubated with 25 µl of anti-HA magnetic beads (Pierce) overnight at 4 °C with gentle agitation. The next day, the beads were washed five times with lysis buffer using a magnetic stand and eluted by boiling with 2× sample buffer. Proteins were separated by SDS–PAGE before immunoblot analysis.

### PLA

A total of 1 × 10^5^ cells were seeded onto glass coverslips. The following day, the cells were fixed with formalin, permeabilized with 0.5% Triton X-100 in PBS, blocked and probed with primary antibodies overnight at 4 °C (Reporting Summary). PLA experiments were performed with Duolink PLA reagents (Sigma-Aldrich) following the manufacturer’s protocol.

Cells were transfected 24 h before fixation with GenJet DNA transfection reagent (SignaGen Laboratories) according to the manufacturer’s instructions. Then, PLA experiments were performed with Duolink PLA reagents followed by immunofluorescence using anti-HA antibodies (Reporting Summary), and Alexa Fluor 488-conjugated secondary antibodies (Invitrogen) to detect transfected cells.

Confocal images were taken on the Zeiss LSM780 microscope (Zeiss) using a Plan Apochromat ×63/NA 1.4 oil immersion objective, with 405-, 488- and 543-nm laser excitation at a pixel resolution of 132 nm. The *Z*-stacks were acquired every 500 nm to ensure a count of the PLA puncta over the whole cell volume. The percentage of cells with PLA punctate structures was obtained by counting at least 65 cells in each working condition from three independent experiments. Puncta detection was performed using a Fiji tailor-made macro^65^. Briefly, the macro segmented the nuclei by thresholding the DAPI signal and using the Analyze Particles ImageJ plugin. The nuclei mask was then applied on the PLA channel, and puncta were detected using the Find Maxima plugin (red channel). Finally, the numbers of dots and nuclei were automatically counted. The Fiji tailor-made macro was modified to select the green nuclei after segmentation by thresholding the DAPI signal to ensure quantification of the specific signal from transfected cells. The green nuclei mask was applied on the PLA channel (red) to determine the number of dots.

For statistical analysis, PLA signal/nucleus quantifications with a 0.1 added constant were log_2_-transformed. A linear mixed-effects model was fitted with the R package lmerTest^66^ using the transformed values as the response variable, the different conditions as the covariate of interest, the biological replicate as the adjusting factor, and the technical replicate as the random effect to account for the technical replicates variability. Adjustments for multiple testing (single-step correction method) were performed using the R package multcomp^67^.

For PLA experiments with ER truncation constructs, dsDNA sequences for AF1 and AF2 domains were designed and synthesized (Geneart Gene Synthesis, Thermo Fisher Scientific) as truncations of the *ESR1* gene, as previously reported^25^, and tagged with a 9X histidine tail. AF1 and AF2 dsDNA sequences were subcloned in pBabe plasmid with puromycin resistance (1764, Addgene) to generate his-tagged (9X) AF1 pBabe-puro and AF2 pBabe-puro via Gibson assembly (NEB), respectively, and according to the manufacturer’s instructions. After puromycin selection, MDA-MB-231 cells were transfected with pCMV5 plasmids for HA-tagged MAF expression (short and long isoforms) or an empty plasmid control using GenJet DNA transfection reagent (SignaGen Laboratories), according to the manufacturer’s instructions. The PLA protocol was then performed as described above.

### PROTAC for targeted degradation

PROTAC ER degrader^24^ was prepared in DMSO, and different concentrations were tested. The final experiments were performed by adding vehicle control (DMSO) or PROTAC to the cell culture media for 24 h before immunoblots, PLA analyses, co-IP or RNA- and ATAC-sequencing experiments.

### RNA-seq

RNA-seq experiments were performed with three biological replicates of control and *MAF*-overexpressing MCF7 cells treated with 10 nM E2 or vehicle for 6 h. RNA was extracted using a PureLink RNA mini kit (Invitrogen) following the manufacturer’s instructions. RNA samples were quantified using Qubit fluorometric quantification (Thermo Fisher Scientific) and the quality was evaluated using Bioanalyzer (Agilent). Libraries were prepared at the Institute for Research in Biomedicine (IRB Barcelona) and sequencing was performed at the Centre for Genomic Regulation (CRG) Genomics Unit, using 1 µg of total RNA and the Illumina HiSeq2500 sequencer.

#### Preprocessing and differential expression analysis

Reads were aligned to the hg19 genome using STAR 2.7.0e^68^, with outFilterMismatchNoverLmax = 0.05 and all other parameters set to default values. Counts per genomic feature were computed with the R^69^ package Rsubread^70^ and the function featureCounts. Differential expression between conditions, taking the sequencing round as the adjusting factor, was performed using DESeq2 v1.22.1^71^. Comparisons of MAF versus mock, mock E2 versus mock, MAF E2 versus MAF, MAF E2 versus mock E2, and MAF E2 versus mock (with condition versus control labelling) were all considered for hypothesis testing. The regularized log-transformed matrix was used for both selecting candidates and visualization purposes.

Genes with an average of ≤5 reads were filtered out. Six different clusters of genes were determined: (1) MAF upregulated; (2) MAF downregulated; (3) E2 upregulated; (4) E2 downregulated; (5) MAF and E2 upregulated; (6) MAF and E2 downregulated.

For each cluster of genes, statistically enriched GO terms were identified using the standard hypergeometric test. Significance was defined by the adjusted *P* value using the Benjamini and Hochberg multiple testing correction.

### RNA-seq upon ER degradation

RNA-seq experiments were performed with three biological replicates of control and *MAF*-overexpressing MCF7 cells treated with 1 µM PROTAC or DMSO for 24 h, followed by 10 nM E2 or vehicle for 6 h. RNA extraction, library construction and data preprocessing followed the same protocols as described above. Sequencing was performed at the IRB Barcelona Functional Genomics Unit, using the Illumina NextSeq550 sequencer.

Reads were aligned to the hg19 genome (hsapiens_gene_ensembl (GRCh37.p13) and TxDb.Hsapiens.UCSC.hg19.knownGene (v3.2.2)) using STAR 2.7.0b^68^ with outFilterMismatchNoverLmax = 0.05 and all others parameters set to default values. Counts per genomic feature were computed with the R^69^ package Rsubread^70^ with the function featureCounts. For the DMSO and PROTAC samples, independently, differential expression between conditions (taking the replicate as the adjusting factor) was performed using the DESeq2 R package^71^. Comparisons of MAF versus mock, mock + E2 versus mock, MAF + E2 versus MAF, MAF + E2 versus mock + E2 and MAF + E2 versus mock (with condition versus control labelling) were all considered for hypothesis testing. A heatmap of the regularized log-transformed and scaled matrix was presented for a subset of genes of interest.

### RNA-seq (comparison to samples after *KDM1A* knockdown)

RNA-seq experiments were performed with two biological replicates of *MAF*-overexpressing MCF7 cells treated with 10 nM E2 or vehicle for 6 h upon KDM1A knockdown. Treatment, extraction, library construction and data preprocessing followed the same protocols as described above.

#### Graphical representation (heatmap of cluster 5/6)

Both shSc and shKDM1A sets of samples were normalized together using the DESeq2 rlog function, ignoring the batch without shKDM1A samples to remove any possible source of technical variability. The average normalized expression for MAF-shSc, MAF-shKDM1A, MAFE2-shSc and MAFE2-shKDM1A was calculated. MAFE2 versus MAF expression differences in shSc and shKDM1A samples were further centred for all genes in clusters 5 and 6.

### Reverse transcription quantitative polymerase chain reaction (qRT–PCR)

Total RNA from control or *MAF*-overexpressing MCF7 cells treated with 10 nM E2 or vehicle for 6 h was extracted using a PureLink RNA mini kit (Invitrogen) following the manufacturers’ instructions. RNA quality and quantity were evaluated using a NanoDrop One spectrophotometer (Thermo Fisher Scientific). cDNA was generated by reverse transcription from 1 μg of RNA using a high-capacity cDNA reverse transcription kit (Applied Biosystems) and a C1000 Touch thermal cycler (BioRad). Real-time PCR reactions were performed using TaqMan universal PCR master mix and specific TaqMan probes (both from Applied Biosystems; Supplementary Table 7). Expression values were normalized to the housekeeping gene *GAPDH* using the comparative Cycle Threshold (CT) method. Statistical significance was assessed using a two-tailed unpaired Student’s *t*-test.

### Correlation and survival data analysis of patients’ cohorts

TCGA BCa counts data^39^ were log_2_ (+0.25 to avoid zero misspecifications) transformed and quantile-normalized. Only stage I, II and III tumours were considered for analysis. Expression values in a log scale were adjusted genewise by HER2 status and tumour stage information.

The METABRIC expression matrix^38,72^ for stage I, II and III tumours only was adjusted genewise by HER2 status, tumour grade, lymph nodes (in log scale), breast surgery status, chemotherapy status, tumour stage and hormone therapy status.

The MSKCC/EMC cohort containing four micro-array studies (GSE2034, GSE2603, GSE5327 and GSE12276) was processed and merged following the same strategy as in a previous publication^13^. The ER status was imputed using the ESR1 intensity, as the pathological status was not available for all studies^13^.

#### Correlation analysis

The Spearman correlation between MAF and several target genes was estimated using the TCGA and METABRIC corrected expression matrices. Assessing the statistical significance of the estimated correlation values was performed with the cor.test R function.

#### Survival analysis

ER binding sites were assigned a gene using Homer annotations^59^. Those genes in cluster 1 of the RNA-seq with an associated ER binding site in both MAF and MAFE2 conditions defined the MAF-tested signature.

The bone relapse prognostic value of this MAF-tested signature was evaluated in the MSKCC/EMC cohort. A Kaplan–Meier representation of the survival curve was considered by distinguishing between two groups of samples: lowly expressed (expression of MAF-tested signature smaller than minus one standard deviation from the mean) and mid/highly expressed (expression of MAF-tested signature larger than minus one standard deviation from the mean) samples. Significance was determined using the log-rank test.

### In vitro BrdU incorporation assay

Control or *MAF*-overexpressing MCF7 cells were cultured in hormone-deprived medium for 72 h, then the cells were treated with 10 nM E2 or vehicle for 24 h. For BrdU incorporation, cells were treated with 10 μM BrdU for 4 h before collection. Finally, the cells were collected and cell pellets were fixed in 10% buffered formalin (Sigma) for 24 h and embedded in paraffin.

### Histopathology and immunohistochemistry

For routine histological analyses, tissues and cell pellets were fixed in 10% buffered formalin (Sigma) and embedded in paraffin. For histopathological analysis, samples were sectioned and stained by conventional haematoxylin and eosin (H&E). Antibodies used for immunostaining include those raised against ER [CRET94D] (CNIO), CK18 (Abcam, ab668), CK17 (Abcam, ab109725), p63 (IR662, Dako-Agilent), BrdU (ab8955, Abcam), MAF (Inbiomotion), GFP (Amsbio, TP401) and RFP (Rockland, 600-401-37).

### Cell proliferation assays and half-maximum inhibitory concentration (IC_50_) determination

Control or *MAF*-overexpressing cells were cultured in hormone-deprived medium for 72 h, then cells were plated at 2,000 cells per well in triplicates in 96-well plates and treated with 10 nM E2 or vehicle 24 h after plating. Proliferation was assessed at the indicated time points using a CyQUANT Cell Proliferation kit according to the manufacturer’s instructions, and the cell number was quantified using a Biotek FL600 fluorescence microplate reader at 485–530 nm.

For 4-OHT IC_50_ calculations, mTB cells were plated at 2,000 cells per well in triplicates in 96-well plates and grown for 24 h. Cells were treated with a dilution series of 4-OHT (Merck). Control cells were incubated with medium containing EtOH. Cell viability was assessed as previously described with CyQUANT after 48 h of treatment. To calculate the IC_50_, values were plotted against the inhibitor concentrations and fit to a sigmoid dose–response curve using GraphPad software.

### ATAC-seq

ATAC-seq experiments were performed as described in ref. ^73^. Briefly, 50,000 control or *MAF*-overexpressing MCF7 cells treated with 10 nM E2 or vehicle for 1 h were collected and treated with transposase Tn5 (Nextera DNA library preparation kit, Illumina). DNA was purified using a PureLink quick gel extraction and PCR purification combo kit (Invitrogen). All samples were PCR-amplified using NEBNextHigh-Fidelity 2× PCR Master Mix (New England BioLabs) with primers containing a barcode to generate libraries and a C1000 Touch Thermal Cycler (BioRad). The DNA was purified again using a PureLink quick gel extraction and PCR purification combo kit. Samples were quantified using Qubit fluorometric quantification (Thermo Fisher Scientific) and the quality was evaluated using Bioanalyzer (Agilent). Sequencing was performed at the Centre for Genomic Regulation (CRG) Genomics Unit, using the Illumina HiSeq2500 sequencer.

#### Preprocessing and peak calling

Cleaning of adapters was completed using Trimmomatic v0.38^74^. Cleaned reads were then aligned to the hg19 human genome using Bowtie2 v2.2.2^75^ with the ‘very sensitive’ option and the remaining parameters set to default values. Genrich v0.5^76^ was employed to call peaks, separately for samples in mock, samples in mock E2, samples in MAF and samples in MAF E2 (options -j -y -r -e chrM). Duplicate sequences were filtered out, sorted and indexed using sambamba v-0.6.7^77^. The union of called peaks by Genrich (consensus peaks) was annotated with Homer^59^ and used for downstream analysis. Peaks were further labelled in three broad categories: intergenic, gene body (exon, intron, 3′UTR, 5′UTR, TTS from homer annotations) and promoter-TSS (±2-kbp TSS range). Counts per consensus peaks were obtained for every sample using the featureCounts function, R package Rsubread^70^, ignoring duplicated reads.

For every peak, distance to the nearest TSS was estimated with Homer^59^. Log counts per peak/sample (using all consensus peaks) were normalized by size factors and aggregated for equally distanced bins of size 25 bp (considering only peaks within 25-kbp distance to a TSS). The resulting intensities were averaged out by condition. The difference between any of MAF, MockE2, MAFE2 versus mock was considered for graphical representation using the smooth.spline R function.

#### Differential analysis and clustering ATAC-seq consensus peaks

Differential chromatin states between all pairwise conditions (mock, mock E2, MAF and MAF E2) were assessed using DESeq2 v1.22^71^ considering the sequencing round as the adjusting variable. ATAC-peak targets were clustered in six groups based on their signal in E2, MAF and E2 + MAF samples. Only peaks within 20-kbp TSS distance were considered. Clusters A and B (MAF-regulated peaks), A positively and B negatively regulated, presented a test-statistic greater than 2 (for A) or smaller than −2 (for B) in both MAF versus mock and MAF E2 versus mock E2 comparisons. Similarly, clusters C and D (E2-regulated peaks), C positively and D negatively regulated, consisted of peaks that had a test-statistic greater than 2 (for C) or smaller than −2 (for D) in both MAF E2 versus MAF and mock E2 versus mock comparisons. Finally, clusters E and F (MAF + E2-regulated peaks), E positively and F negatively regulated, were determined by the consensus peaks that had a test-statistic greater than 2 (or smaller than −2 for F) in both MAF E2 versus MAF and MAF E2 versus mock E2 comparisons.

Overlap regions between clustered peaks and H3K27ac/H3K4me3 histone marks were found using the IRanges R function findOverlaps (parameter maxgap=100). Peak breadth was determined by log_10_ of the peaks’ width. De novo motif analysis on clusters A–F was performed using Homer^59^ (findMotifsGenome.pl function with default parameters).

#### Statistical analyses

Independently for every cluster, the percentage of promoter-TSS chromatin opening was compared to the observed genome distribution of all consensus peaks. Similarly, the percentage of peaks with an overlap with either H3K27ac or H3K4me3 histone marks was compared to the expected overlap in all consensus peaks. In both cases, significance was determined by using a one-tailed permutation test (with 1,000 iterations). Peak breadth mean differences between Promoter-TSS and Intergenic peaks were assessed with a two-tailed Wilcoxon rank sum test.

### ATAC-seq upon ER degradation

MCF7 cells were treated with 1 µM PROTAC or DMSO for 24 h, followed by 10 nM E2 or vehicle for 1 h. Sample collection, DNA tagmentation, amplification, library preparation and data preprocessing followed the same protocols as described above. Sequencing was performed at the IRB Barcelona Functional Genomics Unit, using the Illumina NextSeq550 sequencer.

#### Preprocessing and peaks calling

Reads were aligned to the hg19 human genome using Bowtie2 v2.3.5.1 with the ‘very sensitive’ option, and the remaining parameters were set to default values. Genrich v0.5^76^ was employed to call peaks, separately for samples in mock, samples in mock E2, samples in MAF and samples in MAF E2 for both DMSO and PROTAC data (options -j -y -r -e chrM). Duplicate sequences were filtered out, sorted and indexed using sambamba v-0.6.7^77^. The union of called peaks by Genrich (consensus peaks) was annotated with Homer and used for downstream analysis. Counts per consensus peaks were obtained for every sample using the featureCounts R function, package Rsubread, ignoring duplicated reads. Differential analysis of consensus peaks was performed with DESeq2 for the comparison of MAF E2 versus MAF. This was done independently for PROTAC and DMSO samples, taking the replicate as an adjusting variable.

### Integration of ATAC-seq and RNA-seq data

To integrate the ATAC-seq and RNA-seq data, thresholds to obtain ATAC-seq clusters were relaxed as follows: cluster A (or B) candidate peaks presented a test-statistic greater than 1.5 (or smaller than −1.5 for B) in both MAF versus mock and MAF E2 versus mock E2 comparisons. Cluster C (or D) candidate peaks had a test-statistic greater than 1.5 (or smaller than −1.5 for D) in both MAF E2 versus MAF and mock E2 versus mock comparisons. Cluster E (or F) candidate peaks had a test-statistic greater than 1.5 (or smaller than −1.5 for F) in both MAF E2 versus MAF and MAF E2 versus mock E2 comparisons. For all clusters, only those peaks classified as promoter-TSS were considered. For clusters A and E, we further selected those peaks that were considered as broad peaks (peak breadth >3).

### ER and MAF ChIP-seq

Four 15-cm plates per condition were prepared at 70–80% confluency. After treating control (mock-infected) or *MAF*-overexpressing MCF7 cells with 10 nM E2 or vehicle for 1 h, cells were crosslinked in 1% formaldehyde in DMEM for 10 min at room temperature. Next, glycine was added to a final concentration of 0.125 M and cells were incubated for 5 min at room temperature to stop the fixation. After two washes with ice-cold PBS, cells were collected by gently scrapping on ice and centrifuged at 3,000*g* for 5 min. Cell pellets were stored at 80 °C until use. Chromatin preparation and ChIP experiments were performed with the ChIP-IT High Sensitivity Kit (Active Motif) according to the manufacturer’s protocol. ChIPs were performed using 5 μg per ChIP on ER antibodies (Reporting Summary) and control immunoglobulin-G (Abcam). Library preparation was performed at the Centre for Genomic Regulation (CRG) Genomics Unit using the Illumina HiSeq2500 sequencer.

#### Preprocessing and peaks calling

ChIP-seq samples were mapped against the hg19 human genome assembly using Bowtie with the option –m 1 to discard those reads that could not be uniquely mapped to just one region^78^. Model-based Analysis of ChIP-Seq (MACS) was run with the default parameters but with the shift size adjusted to 100 bp to perform the peak calling against the corresponding control sample^79^.

Obtained peaks were annotated with Homer^59^, and the genome distribution of each set of peaks was simplified to the following three broad categories: promoter-TSS was defined in the region between 2 kbp upstream and 2 kbp downstream of the TSS; genic regions corresponded to the rest of the gene (exon, intron, 3′UTR, 5′UTR and TTS from Homer annotations); and the rest of the genome was considered to be intergenic.

#### Graphical representation (heatmaps, boxplots and circos plot)

Coverage tracks were computed for every sample using deepTools^58^, the bamCoverage function with the parameters --binSize 10, --normalizeUsing RPGC and --extendReads 50. The heatmaps displaying the genomic signal around ChIP-seq peak summits were generated using the EnrichedHeatmap^80^ R package. In that regard, normalized data were obtained using the normalizeToMatrix function with the coverage tracks as signal, the peaks regions as targets, and the function parameters extend = 2000, mean_mode = ‘w0’, and w = 50.

Signal-strength boxplots showed the coverage at the summit of each peak corrected by the sequencing depth differences across samples. The circos plot was created using the R package circlize^81^.

Heatmaps, quantifications for boxplots, Venn diagrams and genome-wide profiles were generated with SeqCode^82^.

The MatInspector program^83^ from the Genomatix software was used to identify MAF binding motifs (MARE half and MAF) within ER ChIP-seq peaks identified in the MAF E2 sample. The number of input sequences with at least one match of the MARE (half) or MAF matrix and the number of matches in all input sequences were calculated. The expected match numbers in an equally sized sample of the genome were calculated assuming that the matches were equally distributed in the genome. Motif overrepresentation was computed as the fold factor of match numbers in regions compared to an equally sized sample of the genome. A *z*-score of motif overrepresentation was calculated with a continuity correction using the formula *z* = (*X* − *E* − 0.5)/*S*, where *X* is the number of found matches in ER ChIP-seq peaks identified in the MAF E2 sample, *E* is the expected value and *S* is the standard deviation.

The UCSC genome browser was used to generate the screenshots of ChIP-seq profiles^84^.

### H3K27Ac and H3K4me3 ChIP-seq

Four 15-cm plates per condition were prepared at 70–80% confluency. After treating control (mock-infected) or *MAF*-overexpressing MCF7 cells with 10 nM E2 or vehicle for 1 h, cells were crosslinked in 1% formaldehyde in DMEM for 10 min at room temperature. Next, glycine was added to a final concentration of 0.125 M and cells were incubated for 5 min at room temperature to stop the fixation. After two washes with ice-cold PBS, cells were collected by gently scrapping on ice and centrifuged at 3,000*g* for 5 min. Pellets were stored at 80 °C until use.

For chromatin preparation, cells were lysed in hypotonic lysis buffer (5 mM PIPES pH 8, 85 mM KCl, 0.5% NP40, plus protease inhibitors). Nuclei were recovered by centrifugation, lysed in nuclear lysis buffer (50 mM Tris-HCl pH 8, 1% SDS, 10 mM EDTA, plus protease inhibitors) and sonicated with a Bioruptor Pico (Diagenode; 20 cycles 30 min on, 30 min off) to yield chromatin fragments of 150–300 bp.

For ChIP experiments, chromatin (25 µg DNA) was diluted 1/10 in ChIP buffer (16.7 mM Tris-HCl pH 8, 167 mM NaCl, 1.2 mM EDTA, 1.1% Triton X-100 plus protease inhibitors) and mixed with 2% *Drosophila* spike-in chromatin. Aliquots were removed as input material (1%). ChIP samples were incubated with the specific antibody (5 µg) plus spike-in antibody (1 µg) overnight 4 °C, and immunoprecipitated with Protein A agarose beads (Diagenode) for 2 h at 4 °C. Beads were washed three times with low-salt buffer (20 mM Tris-HCl pH 8, 150 mM NaCl, 2 mM EDTA, 0.1% SDS, 1% Triton X-100) and once with high-salt buffer (20 mM Tris-HCl pH 8, 400 mM NaCl, 2 mM EDTA, 1% Triton X-100, 0.1% SDS). ChIPed material was eluted from the beads in elution buffer (1% SDS, 100 mM NaHCO_3_) at 65 °C in a shaker (123*g*) for 1 h. Eluted material and inputs were de-crosslinked overnight at 65 °C, treated with Proteinase K and the DNA purified using the QIAquick PCR purification kit (Qiagen).

The following antibodies were used in the ChIP experiments: H3K4me3 (Diagenode, C15410003); H3K27Ac (Millipore, #07–360); *Drosophila* H2Av (Active Motif #61686). Library preparation for ChIP-seq experiments was performed at the UPF/CRG Genomics Unit. Libraries were sequenced using the Illumina HiSeq2000 sequencer.

#### Preprocessing and peaks calling

Sequence reads of human with fly spike-in samples were mapped to the human + fruit fly genome (hg19+dm3) using Bowtie with the option –m 1. MACS^79^ was run with default parameters.

### Integration of ChIP-seq and RNA-seq data

ChIP-seq enrichment curves near E2-induced genes were represented by adopting the methodology proposed in ref. ^14^, taking a linear increase of 10 kb from the TSS (that is, evaluating 10 kb, 20 kb, 30 kb, …, 100 kb distances) and 100 random permutations, showing in grey the fifth top ranked permutation in every tested distance to the TSS.

The sets of binding sites considered for the analysis were selected taking into consideration both MAF-ChIP and ER-ChIP studies: MAF unique (MAF-only sites in response to either MAF or MAFE2 conditions); ER unique (ER-only sites in response to E2); ER unique MAF/E2 (ER-only sites in response to exclusively MAFE2); and shared (ER and MAF overlapping sites in response to E2/MAFE2 and MAF conditions, respectively).

The promoter–enhancer links between BCa ATAC-seq peaks^44^ occupied by MAF/ER binding sites and E2-induced genes, were evaluated following the same strategy as proposed in ref. ^14^. The observed percentages of successful links were compared to 10,000 instances of random selections of ATAC-seq peaks. Circos plots were created using the R package circlize^81^.

### Super-enhancer identification and assignment

Super-enhancers were identified using the ROSE (https://bitbucket.org/young_computation/rose) algorithm. Enhancers were defined by stitching H3k27ac peaks together within 12.5-kb distance. Peaks fully contained within ±2 kbp from a RefSeq promoter were excluded from stitching. These stitched enhancers were then ranked by their ER (MAF/E2) or MAF signal with the input signal subtracted.

### CRISPRi

Control and *MAF*-overexpressing MCF7 cells were transduced with lenti-dCas9-KRAB (Addgene 89567, https://www.addgene.org/89567/) and lentiGuide-puro (Addgene 52963, https://www.addgene.org/52963/) expressing Scramble, PTHLH or JAG1 sgRNAs. For the design of PTHLH and JAG1 constructs, ER chromatin-binding sequences in predicted enhancers near these genes were extracted and scanned using the JASPAR database^85^ to search for predicted MARE binding sites. The top score sequences were cloned and assessed and the one presenting the best downregulation was selected (Supplementary Table 5).

### Circulating tumour cells

A total of 1 × 10^6^ Mock and *MAF*-overexpressing MCF7 cells were resuspended in 1:1 Matrigel and PBS, and injected into the fourth mammary fat pads. When the tumours reached 300 mm^3^, mice where euthanized and blood was collected in tubes containing EDTA. The fluid was transferred to 2-ml plastic tubes and centrifuged for 10 min at 86*g* at 4 °C. The supernatant was discarded. If the pellet was bloody, 1 ml of ACK lysis buffer (Lonza) was added for 5 min at room temperature and, after that, the collected sample was mixed with PBS to a total volume of 10 ml, centrifuged again and decanted. The RNA of the remaining cells was extracted. Human glyceraldehyde 3-phosphate dehydrogenase (GAPDH) and mouse B2M TaqMan probes were used to assess the amount of human versus mouse RNA in mouse blood.

### Cell competition assay

Mock and *MAF*-overexpressing MCF7 cells and Ad-ctrl and Ad-cre (MAF^+^) mTB cells were infected with retroviral particles expressing mCherry-Luc (for control cells) and GFP-Luc (for *MAF*-overexpressing cells). Control-mCherry and MAF-GFP cells were mixed at different ratios (1:9, 1:1 and 9:1) and immediately injected in the tibia, as described above. Bone metastasis growth was tracked by bioluminescence. The composition of metastatic lesions and the distribution of control and MAF^+^ cells were analysed by GFP and mCherry immunohistochemistry.

### BICA

MCF7 cells (2 × 10^5^) were resuspended in 100 µl PBS and inoculated into anaesthetized female BALB/c nude mice (12 weeks old) through intra-illiac artery injection as described in ref. ^86^. Immediately after injection, mice were imaged for luciferase activity using the IVIS SpectrumCT imaging system from Xenogen (Living Image 2.60.1 software). Next, mice were euthanized, and femur and tibia bones were extracted, fragmented using micro dissecting scissors and forceps, and transferred to 96-well plates in a cell culture hood.

To achieve KDM1A inhibition, bone fragments derived from multiple animals were randomized and mixed in each group and cultured in DMEM/F12 media with 5% FBS containing DMSO or 10 µM ORY-1001. The medium was changed every 2–3 days and tumour cell growth was monitored weekly using the IVIS imaging system. Values were calibrated within mouse by subtracting the minimum value. The log_2_-scaled luminescence intensity was normalized to the mean value in day 1 within mouse, and then subjected to statistical analysis. For BICA experiments using MCF7 cells with KDM1A downregulation, several bone fragments derived from one mouse per condition were used. Values were calibrated within mouse by subtracting the minimum value. The log_2_-scaled luminescence intensities were normalized to the mean value in day 1 within mouse. Differences between ORY-1001 and DMSO were assessed by a linear mixed-effects model using the lmerTest R package^66^, with treatment, day and their interaction as fixed effects and mouse as random effect.

### Statistics and reproducibility

For the statistical analyses, R and GraphPad software were used. A minimum of three biological independent samples were required for statistical significance. For animal experiments, each hind limb was an independent sample. Fisher’s exact tests were used for binomial variables, and Kaplan–Meier estimates and log-rank test for time-to-event data. For continuous variables, Student’s *t*-test was used for normally distributed data (data distributions were assumed to be normal, but this was not formally tested), and the Mann–Whitney/Wilcoxon rank test for non-Gaussian populations. Two-tailed and unpaired tests were used for data analysis unless otherwise stated. Comparison *P* values were adjusted by multiple comparisons using the Benjamini–Hochberg method within each experiment. Spearman’s rank correlation tests were considered to evaluate gene-to-gene associations. Permutation tests were used to evaluate the association between chromatin accessible regions and other genomic features. The threshold for statistical significance was set at 5%. All experiments were reproduced at least three times, unless otherwise indicated. No statistical method was used to predetermine sample size but followed those reported previously^87^. No data were excluded from the analyses. To test treatment response, mice were randomized in each condition to control and treatment groups. The investigators were not blinded to allocation during experiments or outcome, with the exception of immunohistochemistry quantifications.

### Reporting summary

Further information on research design is available in the Nature Portfolio Reporting Summary linked to this Article.

## Online content

Any methods, additional references, Nature Portfolio reporting summaries, source data, extended data, supplementary information, acknowledgements, peer review information; details of author contributions and competing interests; and statements of data and code availability are available at 10.1038/s41556-023-01281-y.

### Supplementary information

Reporting Summary

Supplementary Table 1 List of differentially expressed genes in each of the defined clusters by RNA-seq.

Supplementary Table 2 Hallmark enrichment analysis in the defined clusters by RNA-seq.

Supplementary Table 3 Homer motif enrichment analysis in MAF-ER shared ChIP-seq peaks considering all MAF ChIP-seq peaks as background.

Supplementary Table 4 Homer motif enrichment analysis on ATAC-seq clusters A–F.

Supplementary Table 5 Primers, strings and shRNAs sequences.

Supplementary Table 6 Input and output SAINTexpress data for MCF7 and MDA-MB-231 BioIDs.

Supplementary Table 7 TaqMan probes.

### Source data

Source Data Fig. 1 Statistical source data.

Source Data Fig. 1 Unprocessed western blots.

Source Data Fig. 2 Statistical source data.

Source Data Fig. 2 Unprocessed western blots.

Source Data Fig. 3 Statistical source data.

Source Data Fig. 4 Statistical source data.

Source Data Fig. 7 Statistical source data.

Source Data Fig. 7 Unprocessed western blots.

Source Data Extended Data Fig./Table 1 Statistical source data.

Source Data Extended Data Fig./Table 1 Unprocessed western blots.

Source Data Extended Data Fig./Table 2 Statistical source data.

Source Data Extended Data Fig./Table 2 Unprocessed western blots.

Source Data Extended Data Fig./Table 3 Statistical source data.

Source Data Extended Data Fig./Table 3 Unprocessed western blots.

Source Data Extended Data Fig./Table 4 Statistical source data.

Source Data Extended Data Fig./Table 5 Statistical source data.

Source Data Extended Data Fig./Table 5 Unprocessed western blots.

Source Data Extended Data Fig./Table 9 Statistical source data.

Source Data Extended Data Fig./Table 9 Unprocessed western blots.

Source Data Extended Data Fig./Table 10 Statistical source data.

Source Data Extended Data Fig./Table 10 Unprocessed western blots.

## Data Availability

RNA-seq, ChIP-seq and ATAC-seq data that support the findings of this study have been deposited in the Gene Expression Omnibus (GEO) under SuperSeries accession codes GSE210608 and GSE232389. Mass spectrometry data have been deposited in the PRIDE repository with the primary accession code PXD035936 (https://www.ebi.ac.uk/pride). The human breast cancer data were derived from the TCGA Research network at https://portal.gdc.cancer.gov/projects/TCGA-BRCA. The METABRIC dataset was derived from the Molecular Taxonomy of Breast Cancer International Consortium at https://ega-archive.org/studies/EGAS00000000083. The MSKCC/EMC data were derived from the three cohorts from these institutions. The datasets that support the findings of this study are available from the GEO under accession codes GSE2034, GSE2603, GSE5327 and GSE12276. All other data supporting the findings of this study are available from the corresponding author on reasonable request. Source data are provided with this paper.
