# Supplementary material for: MAF amplification licenses ERα through epigenetic remodelling to drive breast cancer metastasis
Source: Nat Cell Biol. 2023 Nov 9;25(12):1833–47. doi: 10.1038/s41556-023-01281-y (PMC10709142; doi:10.1038/s41556-023-01281-y)
Supplement: Supplementary file 1 — Reporting Summary [file 41556_2023_1281_MOESM1_ESM.pdf]

nature portfolio

Corresponding author(s): Roger R. Gomis, Maria T. Blasco

Last updated by author(s): 21 SEPTEMBER, 2023

Reporting Summary

Nature Portfolio wishes to improve the reproducibility of the work that we publish. This form provides structure for consistency and transparency in reporting. For further information on Nature Portfolio policies, see our Editorial Policies and the Editorial Policy Checklist.

Statistics

For all statistical analyses, confirm that the following items are present in the figure legend, table legend, main text, or Methods section.

n/a	Confirmed	
☐	☒	The exact sample size (*n*) for each experimental group/condition, given as a discrete number and unit of measurement
☐	☒	A statement on whether measurements were taken from distinct samples or whether the same sample was measured repeatedly
☐	☒	The statistical test(s) used AND whether they are one- or two-sided<br>*Only common tests should be described solely by name; describe more complex techniques in the Methods section.*
☐	☒	A description of all covariates tested
☐	☒	A description of any assumptions or corrections, such as tests of normality and adjustment for multiple comparisons
☐	☒	A full description of the statistical parameters including central tendency (e.g. means) or other basic estimates (e.g. regression coefficient) AND variation (e.g. standard deviation) or associated estimates of uncertainty (e.g. confidence intervals)
☐	☒	For null hypothesis testing, the test statistic (e.g. *F*, *t*, *r*) with confidence intervals, effect sizes, degrees of freedom and *P* value noted<br>*Give P values as exact values whenever suitable.*
☒	☐	For Bayesian analysis, information on the choice of priors and Markov chain Monte Carlo settings
☒	☐	For hierarchical and complex designs, identification of the appropriate level for tests and full reporting of outcomes
☒	☐	Estimates of effect sizes (e.g. Cohen's *d*, Pearson's *r*), indicating how they were calculated

Our web collection on statistics for biologists contains articles on many of the points above.

Software and code

Policy information about availability of computer code

Data collection	Histological images acquisition: Zeiss LSM780 microscope, NanoZoomer-2.0 HT C9600 scanner (Hamamatsu) equipped with a 20x objective.<br>Real time PCRs: QuantStudio Real Time-PCR Software<br>Intravital bioluminiscence: Living Image versions 2.60.1 and 4.5.5 19626 (64bit)<br>Illumina HiSeq2500 sequencer, Illumina NextSeq550 sequencer
Data analysis	We used LivingImage software (versions 2.60.1 and 4.5.5 19626 (64bit), R.Q manager 1.2 software, NPD.view 2 U12388-01 software version 2.9.25, DataAssist 3.01 software, ImageJ version 1.53K, QuPath (v0.1.2), HOMER suite 5.0 software, GraphPad Prism 8 software, Sambamba (v-0.6.7), featureCounts function R (v4.0.1), R package Rsubread (v2.4.3), R package multcomp (v1.4-25), R package lmerTest (v3.1-3), cor.test R function, IRanges R function findOverlaps (v2.24.1), R package circlize (v-0.4.15), MACS2 (v2.1.1), ROSE (https//bitbucket.org/young_computation/rose) algorithm. Thermo Proteome Discoverer v2.4.1.15 (PD) and MaxQuant v1.6.14.0 (MQ), using Sequest HT and Andromeda search engine nodes for PD and MQ, respectively. The database used in the search was SwissProt Human (released in October 2020), SAINTexpress-spc v3.6.1, STRING v11 database, STAR 2.7.0e, Trimmomatic v0.38, Bowtie2 v2.2.2, Genrich v0.5, DESeq2 v1.22., MatInspector program.

For manuscripts utilizing custom algorithms or software that are central to the research but not yet described in published literature, software must be made available to editors and reviewers. We strongly encourage code deposition in a community repository (e.g. GitHub). See the Nature Portfolio guidelines for submitting code & software for further information.

Data

Policy information about availability of data

All manuscripts must include a data availability statement. This statement should provide the following information, where applicable:

- Accession codes, unique identifiers, or web links for publicly available datasets
- A description of any restrictions on data availability
- For clinical datasets or third party data, please ensure that the statement adheres to our policy

ChIP-sequencing, ATAC-sequencing and RNA-sequencing data are available at Gene Expression Omnibus (GEO) database:
- ATACSeq raw fastq files available in GEO (GSE210603 and GSE232270). Bowtie Human/hg19 index from UCSC (https://benlangmead.github.io/aws-indexes/bowtie). Homer human annotations v6.3 (http://homer.ucsd.edu/homer/data/organisms/human.v6.3.zip). Comparison to H3K27ac and H3k4me3 histone marks (GSE232706).
- RNASeq raw fastq files available in GEO (GSE210607 and GSE232175). Human/hg19 annotations from Ensembl (GRCh37.p13) and complemented with UCSC (TxDb.Hsapiens.UCSC.hg19.knownGene, v3.2.2). Biological enrichment using Human MSigDB collections (v5.1). Association with TCGA breast cancer cohort (https://portal.gdc.cancer.gov/projects/TCGA-BRCA, RNASeq v1.12.0), Metabric (https://ega-archive.org/studies/EGAS00000000098) and MSKCC/EMC cohort containing four micro-array studies (GSE2034, GSE2603, GSE5327, and GSE12276).
- CHIPSeq raw fastq files available in GEO (GSE210604 and GSE210605). Bowtie Human/hg19 index from UCSC (https://benlangmead.github.io/aws-indexes/bowtie). Homer human annotations v6.3 (http://homer.ucsd.edu/homer/data/organisms/human.v6.3.zip). Association with TCGA breast cancer cohort (https://www.ncbi.nlm.nih.gov/pmc/articles/PMC6408149/, Table S2 and S7, ATACSeq peaks).

All proteomic data can be found at PRIDE repository ID_PXD035936. Thermo Proteome Discoverer v2.4.1.15 (PD), MaxQuant v1.6.14.0 (MQ), using Sequest HT and Andromeda search engine nodes for PD and MQ, respectively. SAINTexpress-spc v3.6.1. Protein-protein interaction data was downloaded from the STRING v11 database.

All other data supporting the findings of this study are available from the corresponding authors upon reasonable request.

Human research participants

Policy information about studies involving human research participants and Sex and Gender in Research.

Reporting on sex and gender	We complement our results and conclusions with the analysis of public gene expression data sets from human breast cancers and metastasis. All ethical and legal aspects of the use of such data are covered and does not require ethical approval as sample anonymizations were compiled by the original authors that generated the public information.<br><br>Sex/Gender dimension – From a biomedical standpoint, the consequences of breast cancer (BCa) affect men and women alike, and so do the related pathologies. BCa incidence in men is rare, and the interaction with estrogen signaling even more, so our main effort contemplates the physiology and disease that are of relevance for females. Our research was performed in female mice for purely pragmatic reasons (female mice are the target, but the underlying biology of basal-like breast cancer is partly shared with male mice). Further, human sample analyses also focused on females. All the Ethical examinations have been approved by Institutional and governmental bodies.
Population characteristics	Does not apply
Recruitment	Does not apply
Ethics oversight	Does not apply

Note that full information on the approval of the study protocol must also be provided in the manuscript.

Field-specific reporting

Please select the one below that is the best fit for your research. If you are not sure, read the appropriate sections before making your selection.

☒ Life sciences ☐ Behavioural & social sciences ☐ Ecological, evolutionary & environmental sciences

For a reference copy of the document with all sections, see nature.com/documents/nr-reporting-summary-flat.pdf

Life sciences study design

All studies must disclose on these points even when the disclosure is negative.

Sample size	No statistical test was used to determined sample size upfront. Instead, sample size was determined empirically based on prior knowledge of the variation in the experimental setup and followed those reported previously (Gawrzak et al). We used at least 7 mice per group (otherwise indicated) for the in-vivo settings, which is sufficient to detect meaningful differences based on historical record with similar experiments. For the majority of in-vitro experiments, we used n equal or higher to 3, according to previous experience with similar experiments.

Data exclusions | Animals were excluded from the study if not properly injected or severe cachexia was reported. Criteria was pre-established. No data from in-vitro experiments were excluded.

Replication | Experimental findings were reliably reproduced. Experiments were performed at least three times unless otherwise noted in the manuscript.

Randomization | Animals, upon arrival or breeding, were randomly allocated into cages with five mice each. The mice were randomly assigned to experimental groups. No specific method of randomization was used. For the comparison of metastasis formation capacity between different cells, the different tumor cell population were injected in a intercalated order to avoid biases as a result of cell viability. For in vivo experiments involving treatments (ORY1001) animals were randomized in treatment groups containing mice with similar tumor burden according to BLI measurements.

Blinding | Investigator was not aware of group allocation when assessing the outcome of bioluminescent analyses. Samples for immunohistochemistry were coded and investigators were blinded during staining and quantification.

Reporting for specific materials, systems and methods

We require information from authors about some types of materials, experimental systems and methods used in many studies. Here, indicate whether each material, system or method listed is relevant to your study. If you are not sure if a list item applies to your research, read the appropriate section before selecting a response.

Materials & experimental systems

n/a	Involved in the study
☐	☒ Antibodies
☐	☒ Eukaryotic cell lines
☒	☐ Palaeontology and archaeology
☐	☒ Animals and other organisms
☒	☐ Clinical data
☒	☐ Dual use research of concern

Methods

n/a	Involved in the study
☐	☒ ChIP-seq
☒	☐ Flow cytometry
☒	☐ MRI-based neuroimaging

Antibodies

Antibodies used

- HA-Tag. Sigma Aldrich, H3663
- HA-Tag. Cell signaling, 3724S
- His-Tag. Thermofisher Scientific, MA1-21315
- ERα. Abcam, ab16660
- ERα. Santa Cruz, sc-543
- MYC-Tag. Cell signaling, 2272
- MAF. Inbiomotion, 130(5)
- c-MAF (E-7). Santa Cruz, sc-518062
- α-Tubulin. Sigma Aldrich, T6199
- ARID1A. Sigma Aldrich, HPA005456
- KDM1A/LSD1. Abcam, ab17721
- MTA1. Santa Cruz, sc-17773
- NCoR1. Santa Cruz, sc-515934
- NCoA3. Santa Cruz, sc-5305
- H3. Abcam, ab1791
- H3K9me2. Abcam, ab1220
- H3K4me2. Abcam, ab7766
- H3K4me3. Diagenode, C15410003
- H3K27Ac. Millipore, #07–360
- Anti-Drosophila H2Av. Active Motif, #61686
- ER [CRET94D]. CNIO
- p63. Dako-agilent, IR662
- CK18. Abcam, ab668
- CK17. Abcam, ab109725
- BrdU. Abcam, ab8955
- GFP. Life Technologies, A11122
- RFP. Rockland 600-401-379
- Rabbit IgG HRP-conjugated. GE HealthCare, NA93 4
- Mouse IgG HRP-conjugated. Thermofisher Scientific, 31452
- Streptavidin HRP-conjugated. Abcam, ab7403
- Streptavidin Alexa Fluor 546-conjugated. Invitrogen, S11225
- Rabbit IgG Alexa Fluor 488-conjugated. Invitrogen, A-11008
- Mouse IgG Alexa Fluor 488-conjugated. Invitrogen, A-11001

Validation

- HA-Tag. Sigma Aldrich, H3663. Applications: western-blot, immunofluorescence, PLA. Dilution: 1:500. Validation: doi.org/10.18632/oncotarget.1817
- HA-Tag. Cell signaling, 3724S. Applications: immunofluorescence, PLA. Dilution: 1:1000. Validation: doi.org/10.1016/

j.gendis.2020.08.0010
- His-Tag. Thermofisher Scientific, MA1-21315. Applications: immunofluorescence, PLA. Dilution: 1:1000. Validation: doi.org/10.1186/s13046-022-02389-z
- ERα. Abcam, ab16660. Applications: western-blot, immunofluorescence, PLA. Dilution: 1:500. Validation: doi.org/10.7554/eLife.15828
- ERα. Santa Cruz, sc-543. Applications: ChIPseq. Amount: 5 micrograms. Validation: doi.org/10.1158/1541-7786.MCR-13-0588 and this paper.
- MYC-Tag. Cell signaling, 2272. Applications: western-blot, immunofluorescence. Dilution: 1:1000. Validation: doi.org/10.1073/pnas.1917675117
- MAF. Inbiomotion, 130(5). Applications: western-blot, immunohistochemistry. Dilution: 1:50. Validation: this paper
- c-MAF (E-7). Santa Cruz, sc-518062. Applications: ChIPseq. Amount: 5 micrograms. Validation: doi.org/10.1155/2021/5517143 and this paper.
- α-Tubulin. Sigma Aldrich, T6199. Applications: western-blot. Dilution: 1:1000. Validation: doi.org/10.1073/pnas.1700234114
- ARID1A. Sigma Aldrich, HPA005456. Applications: western-blot. Dilution: 1:1000. Validation: doi.org/10.1158/0008-5472.CAN-21-0206
- KDM1A/LSD1. Abcam, ab17721.Applications: western-blot. Dilution: 1:1000. Validation: doi.org/10.1016/j.ijbiomac.2020.08.221
- MTA1. Santa Cruz, sc-17773. Applications: western-blot. Dilution: 1:200. Validation: doi.org/10.1186/s12964-019-0318-6
- NCoR1. Santa Cruz, sc-515934. Applications: western-blot. Dilution: 1:200. Validation: doi.org/10.1038/s41467-019-09598-9
- NCoA3. Santa Cruz, sc-5305. Applications: western-blot. Dilution: 1:200. Validation: doi.org/10.1210/en.2010-1281
- H3. Abcam, ab1791. Applications: western-blot. Dilution: 1:1000. Validation: doi.org/10.3390/epigenomes6040036
- H3K9me2. Abcam, ab1220. Applications: western-blot. Dilution: 1:1000. Validation: doi.org/10.1038/s41467-021-25951-3
- H3K4me2. Abcam, ab7766. Applications: western-blot. Dilution: 1:1000. Validation: doi.org/10.4049/jimmunol.2001146
- H3K4me3. Diagenode, C15410003. Applications: ChIPseq. Amount: 5 micrograms. Validation: doi.org/10.1126/sciadv.abh1684
- H3K27Ac. Millipore, #07–360. Applications: ChIPseq. Amount: 5 micrograms. Validation: doi.org/10.7554/eLife.06283
- Anti-Drosophila H2Av. Active Motif, #61686. Amount: 5 micrograms. Applications: ChIPseq. Validation: doi.org/10.1038/s41467-021-21893-y
- ER [CRET94D]. CNIO. Applications: western-blot. Dilution: 1:5. Validation: this paper
- p63. Dako-agilent, IR662. Applications: immunohistochemistry. Dilution: 1:500. Validation: this paper
- CK18. Abcam, ab668. Applications: immunohistochemistry. Dilution: 1:500. Validation: doi.org/10.1038/s41467-020-14737-8
- CK17. Abcam, ab109725. Applications: immunohistochemistry. Dilution: 1:500. Validation: doi.org/10.3389/fmed.2020.572494
- BrdU. Abcam, ab8955. Applications: immunohistochemistry. Dilution: 1:2000. Validation: doi.org/10.1523/JNEUROSCI.0356-15.2015
- GFP. Life technologies A11122. Applications: immunohistochemistry. Dilution: 1:750. Validation: doi.org/10.1038/s41467-021-21426-7
- RFP. Rockland 600-401-379. Applications: immunohistochemistry. Dilution: 1:500. Validation: doi.org/10.7554/eLife.76912
- Rabbit IgG HRP-conjugated. GE HealthCare, NA934. Applications: western-blot. Dilution: 1:5000. Validation: doi.org/10.1038/s41598-021-90284-6
- Mouse IgG HRP-conjugated. Thermofisher Scientific, 31452. Applications: western-blot. Dilution: 1:5000. Validation: doi.org/10.1016/j.yexmp.2011.10.010
- Streptavidin HRP-conjugated. Abcam, ab7403. Applications: western-blot. Dilution: 1:1000. Validation: doi.org/10.1038/s41419-021-03605-y
- Streptavidin Alexa Fluor 546-conjugated. Invitrogen, S11225. Applications: immunofluorescence. Dilution: 1:500. Validation: doi.org/10.4049/jimmunol.176.10.5815
- Rabbit IgG Alexa Fluor 488-conjugated. Invitrogen, A-11008. Applications: immunofluorescence. Dilution: 1:500. Validation: doi.org/10.3892/or.2023.8579
- Mouse IgG Alexa Fluor 488-conjugated. Invitrogen, A-11001. Applications: immunofluorescence. Dilution: 1:500. Validation: doi.org/10.3390/cells12040636

Eukaryotic cell lines

Policy information about cell lines and Sex and Gender in Research

Cell line source(s)	The human BCa cell lines T47D, MCF7, MDA-MB-231 and human embryonic kidney 293T cells, were purchased from ATCC. (MDA-MB-231 ref. HTB-26; MCF7 ref. HTB-22; T47D ref. HTB-133; HEK-293T ref. CRL-1573). The mouse BCa cell mTB was derived from Rosa26LSLMaf female mice subcutaneously injected with 15 mg Medroxyprogesterone acetate (MPA) (Depo-Provera) at 7 weeks of age. One milligram of 7,12-dimethylbenzanthracene (DMBA) was administered weekly by oral gavage during the following 4 weeks. mTB cells were established from freshly isolated tumors following standard procedures.
Authentication	Cell lines were authenticated in our lab for the presence of ER and HER2, by WB, IHC or FISH, respectively. Cell lines were purchased with the certificate from the vendor.
Mycoplasma contamination	Cell lines were tested for mycoplasm routinely in the lab and only mycoplasma-negative cell lines were used.
Commonly misidentified lines (See ICLAC register)	None of the cell lines used in this study was found in the database of commonly misidentified cell lines that are maintained by ICLAC.

Animals and other research organisms

Policy information about studies involving animals; ARRIVE guidelines recommended for reporting animal research, and Sex and Gender in Research

Laboratory animals	All animal work was approved by the Ethical Committee of Animal Experimentation of the Government of Catalonia (protocol numbers 10508-P1 and 9096-P1).<br>Injections of cells were performed in BALB/c nude and FvB/NJ female hosts aged 12 weeks. Also, genetically engineered

Rosa26LSLMaf FvB/NJ female mice were used for MPA-DMBA treatment starting at 8 weeks of age.
Mouse embryonic stem cells derived from C57BL6/129Sv/F1 and blastocyts derived from C57BL6 mice were used to generate chimeras that transmitted MAF transgene to their offspring.
All mice were closely monitored by authors and facility technicians and by an external veterinary responsible for welfare. Mice were maintained in a specific-pathogen-free facility with a 12-h light-dark cycle, under controlled temperature and humidity (18-23°C and 40-60% respectively) and given ad libitum access to standard diet.

Wild animals

The study did not involved wild animals

Reporting on sex

From a biomedical standpoint, the consequences of breast cancer (BCa) affect men and women alike, and so do the related pathologies. BCa incidence in men is rare, and the interaction with estrogen signaling even more, so our main effort contemplates the physiology and disease that are of relevance for females. Our research was performed in female mice for purely pragmatic reasons (female mice are the target, but the underlying biology of basal-like breast cancer is partly shared with male mice). Further, human sample analyses also focused on females. All the Ethical examinations have been approved by Institutional and governmental bodies.

Field-collected samples

This study did not include field collected samples

Ethics oversight

All animal work was approved by The Ethical Committee of Animal Experimentation of the Government of Catalonia (protocol numbers 10508-P1 and 9096-P1).

Note that full information on the approval of the study protocol must also be provided in the manuscript.

ChIP-seq

Data deposition

☒ Confirm that both raw and final processed data have been deposited in a public database such as GEO.

☒ Confirm that you have deposited or provided access to graph files (e.g. BED files) for the called peaks.

Data access links
May remain private before publication.

ChIP-sequencing data have been deposited in the Gene Expression Omnibus (GEO) database. The SuperSeries accession numbers of the data reported in this paper are GSE210608 and GSE232389.

Files in database submission

GSM6433415 MockHD_ER
GSM6433416 InputMockHD_ER
GSM6433417 MockE2_ER
GSM6433418 InputMockE2_ER
GSM6433419 MAFHD_ER
GSM6433420 InputMAFHD_ER
GSM6433421 MAFE2_ER
GSM6433422 InputMAFE2_ER
GSM6433423 MockE2_MAF
GSM6433424 InputMockE2_MAF
GSM6433425 MAFHD_MAF
GSM6433426 InputMAFHD_MAF
GSM6433427 MAFE2_MAF
GSM6433428 InputMAFE2_MAF
GSM7372123 H3K27ac_CT
GSM7372124 H3K27ac_CTE
GSM7372125 H3K27ac_MA
GSM7372126 H3K27ac_MAE
GSM7372127 H3K4me3_CT
GSM7372128 H3K4me3_CTE
GSM7372129 H3K4me3_MA
GSM7372130 H3K4me3_MAE
GSM7372131 INPUT_CT
GSM7372132 INPUT_CTE
GSM7372133 INPUT_MA
GSM7372134 INPUT_MAE

Genome browser session
(e.g. UCSC)

No longer applicable

Methodology

Replicates

We performed genome-wide mapping of H3K27ac, H3K4me3, ER and MAF sites in control and MAF-overexpressing MCF7 cells to assess the consequences of estrogen (E2) stimulation and MAF overexpression recruitment to chromatin. To this end, we cultured MCF7 cells in hormone-deprived (HD) medium for 72 h and then E2 or vehicle was added for 1h prior to chromatin immunoprecipitation (ChIP). Samples were generated in triplicate.

Sequencing depth

All samples contain reads of 50 bps (single-end)

SAMPLE / Total reads / Uniquely mapped reads

	GSM7372123 H3K27ac_CT / 56793272 / 43324730<br>GSM7372124 H3K27ac_CTE / 59873093 / 45735306<br>GSM7372125 H3K27ac_MA / 57813202 / 44362477<br>GSM7372126 H3K27ac_MAE / 56260537 / 43103200<br>GSM7372127 H3K4me3_CT / 57430621 / 47007885<br>GSM7372128 H3K4me3_CTE / 55073014 / 45086353<br>GSM7372129 H3K4me3_MA / 56054578 / 45038112<br>GSM7372130 H3K4me3_MAE / 63116866 / 50763364<br>GSM7372131 INPUT_CT / 55769132 / 43138992<br>GSM7372132 INPUT_CTE / 53439249 / 41360023<br>GSM7372133 INPUT_MA / 51965381 / 40267439<br>GSM7372134 INPUT_MAE / 54697010 / 42409933
Antibodies	- H3K4me3. Diagenode, C15410003<br>- H3K27Ac. Millipore, #07–360<br>- ERα. Santa Cruz, sc-543<br>- c-MAF (E-7). Santa Cruz, sc-518062<br>- Anti-Drosophila H2Av. Active Motif #61686
Peak calling parameters	(1 mapping)<br>$command = "bowtie -p 4 -t -m 1 -S -q $GENOME/genome $fastq_file $MAPS/$name.sam 2> info/bowtie_$name.txt";<br>$GENOME = "\$HUMAN19_FLY3_SPIKEIN";<br>(2 peak calling)<br>$command = "macs14 -t $bam_file1 -c $bam_file2 $GENOME -n $name1 $profiles --nomodel --shiftsize 100 2> $newdir/info.txt";
Data quality	All peaks retrieved by MACS using default cutoffs were used in the study<br>Pvalue cutoff for peak detection. DEFAULT: 1e-5
Software	BOWTIE for mapping, MACS for peak calling, SEQCODE for downstream analysis
