# Supplementary figures and images for: MAF amplification licenses ERα through epigenetic remodelling to drive breast cancer metastasis
Source: Nat Cell Biol. 2023 Nov 9;25(12):1833–47. doi: 10.1038/s41556-023-01281-y (PMC10709142; doi:10.1038/s41556-023-01281-y)

Figure 1f

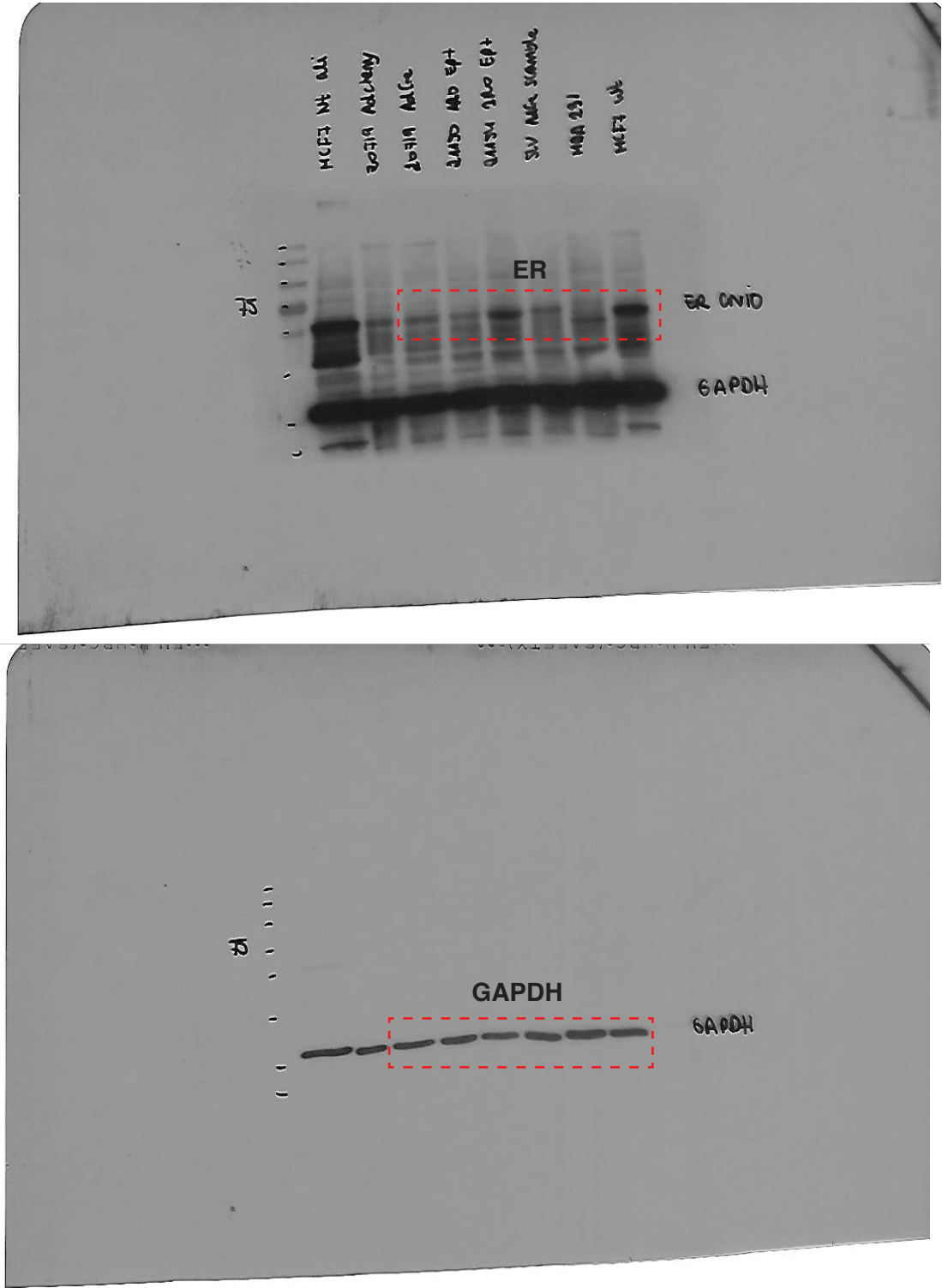

Figure 1h

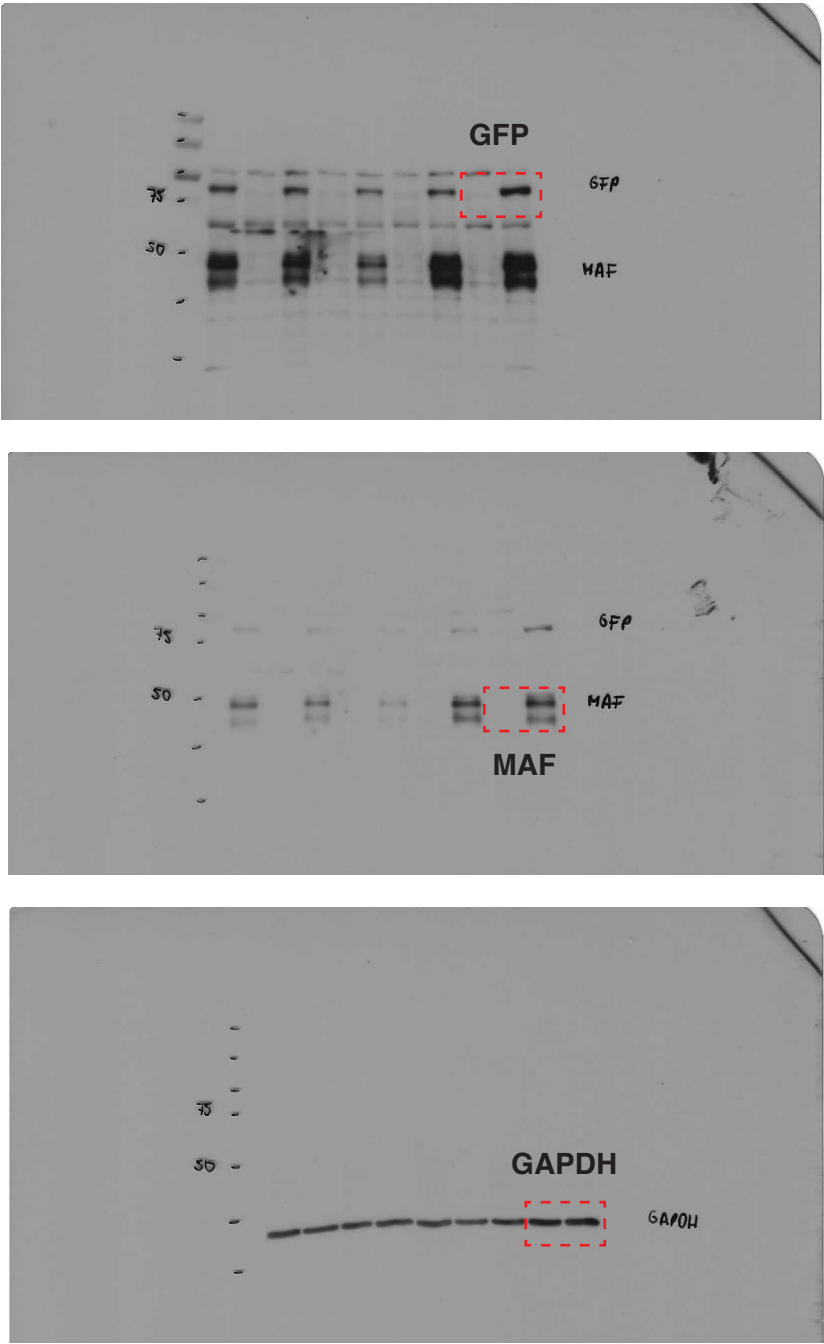

Supplement: Supplementary file 10 — Unprocessed western blots. [file 41556_2023_1281_MOESM10_ESM.pdf]

Figure 2d

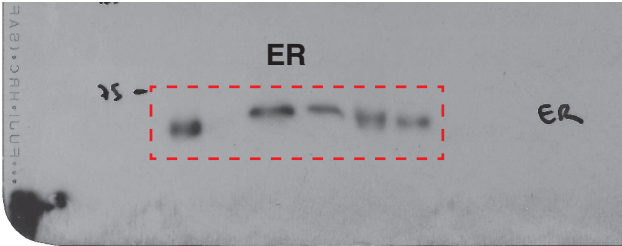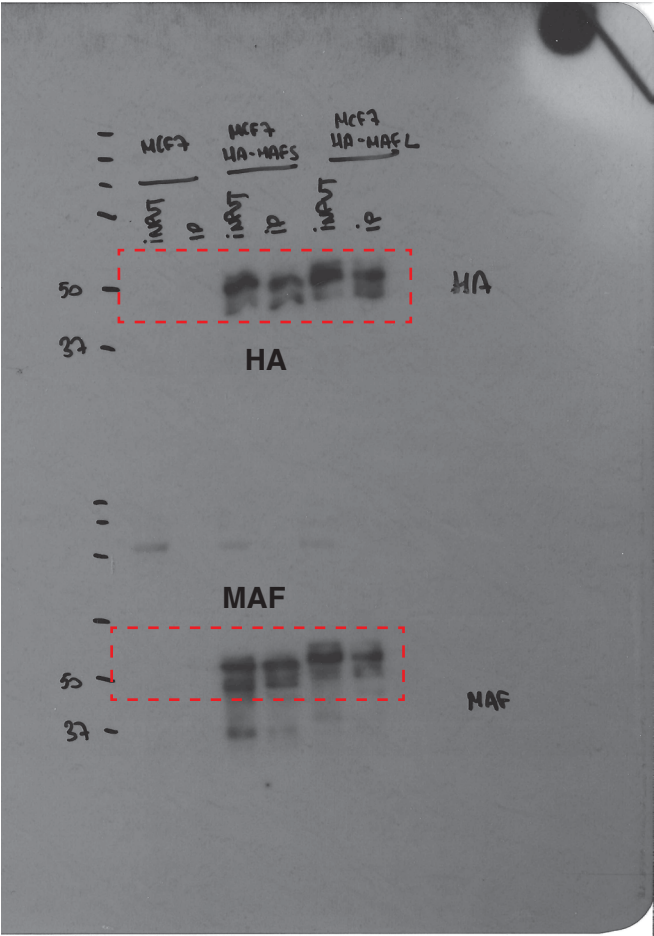

Figure 2g

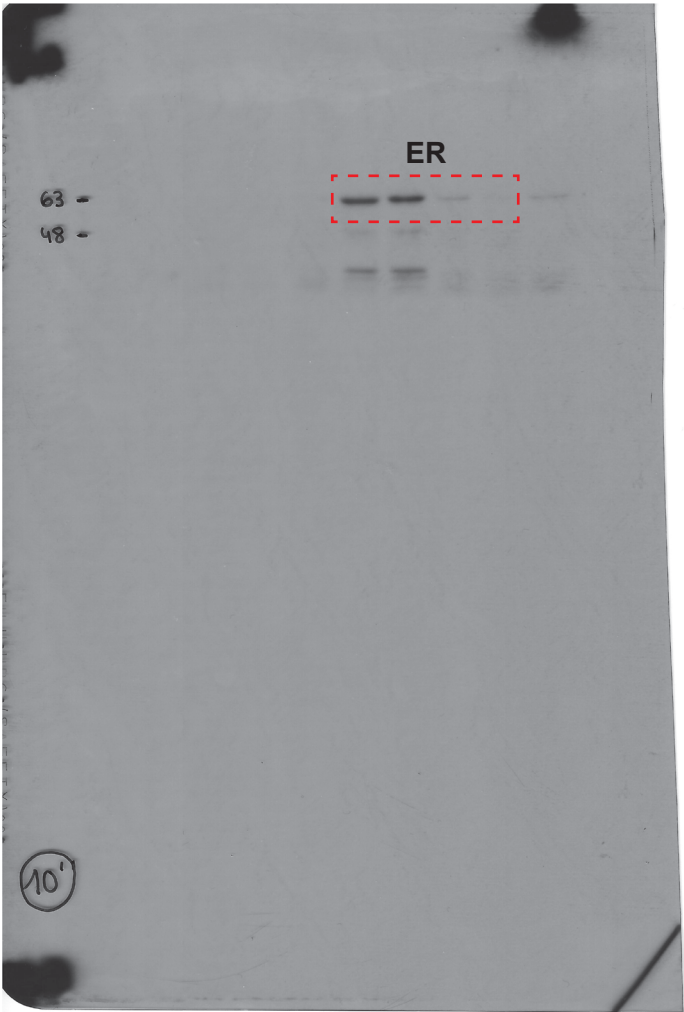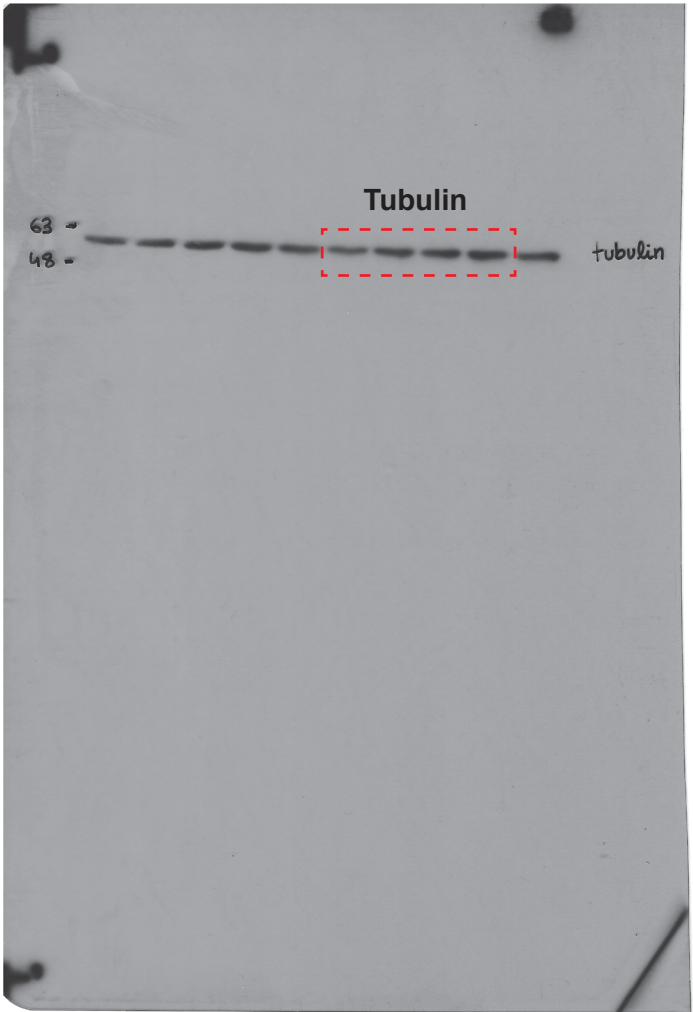

Supplement: Supplementary file 12 — Unprocessed western blots. [file 41556_2023_1281_MOESM12_ESM.pdf]

Figure 7b

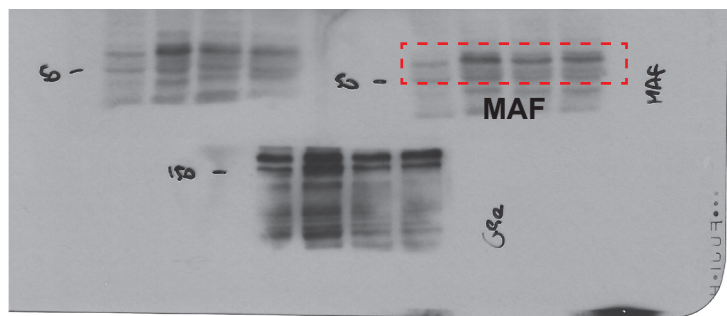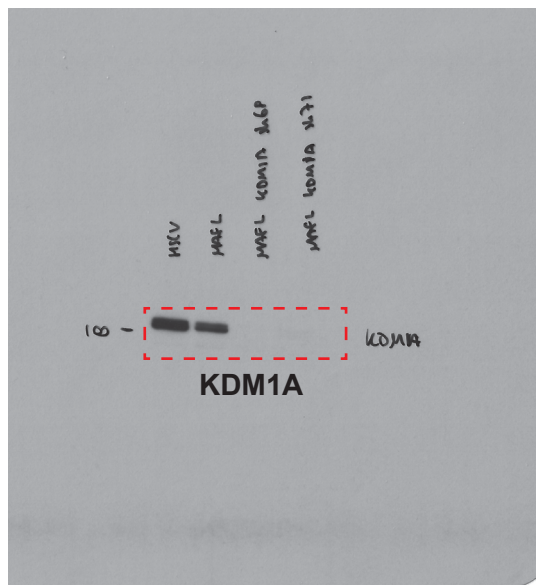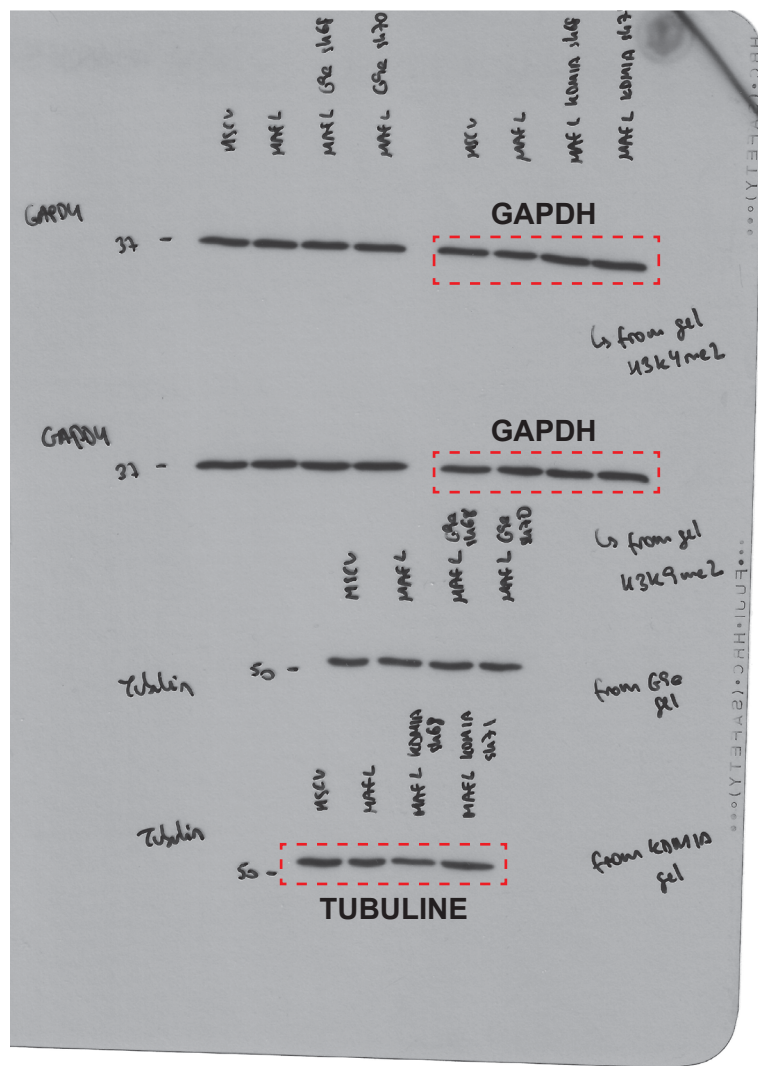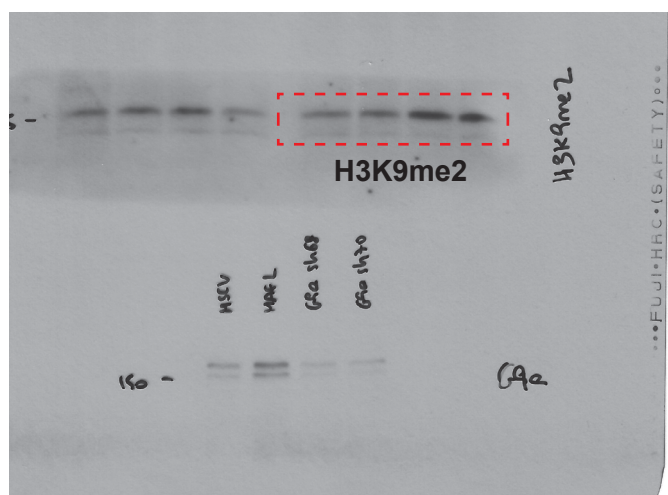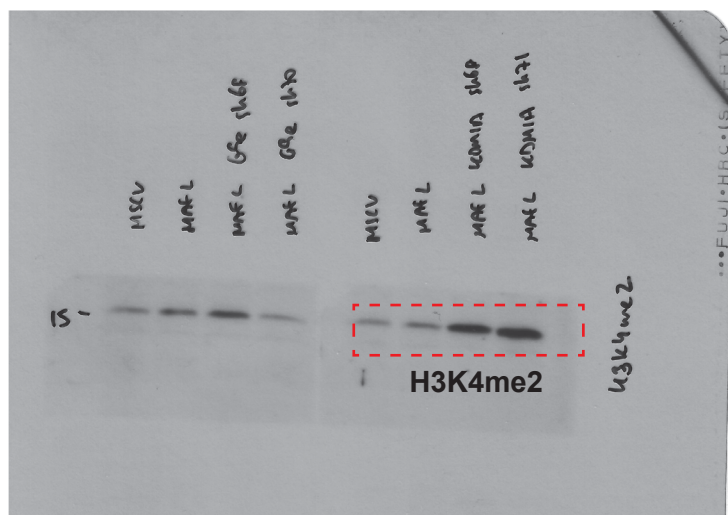

Figure 7f

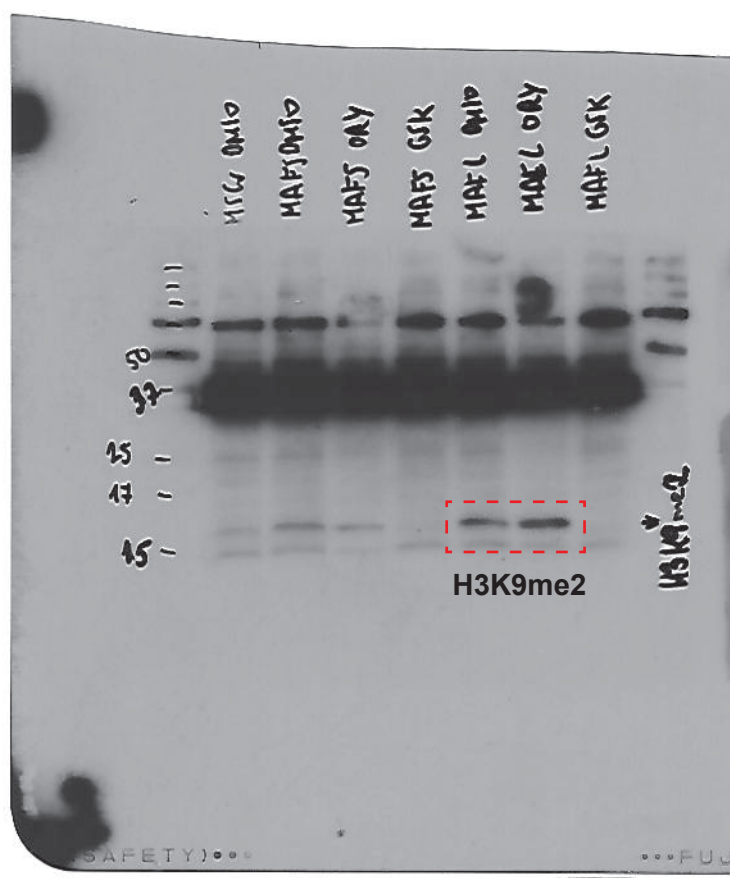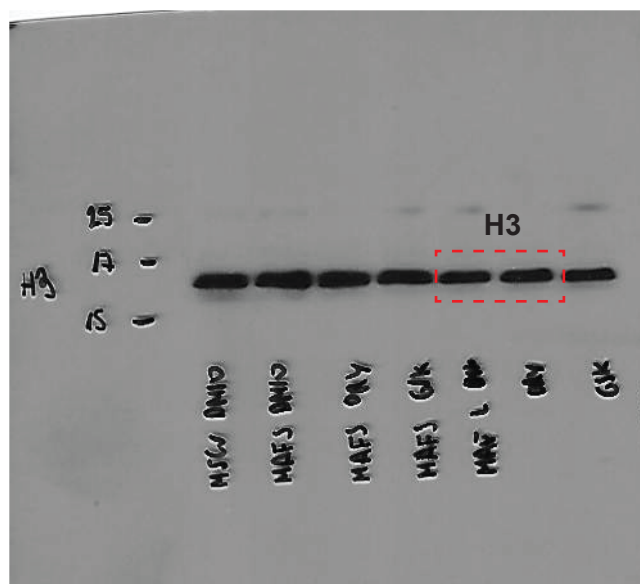

Figure 7g

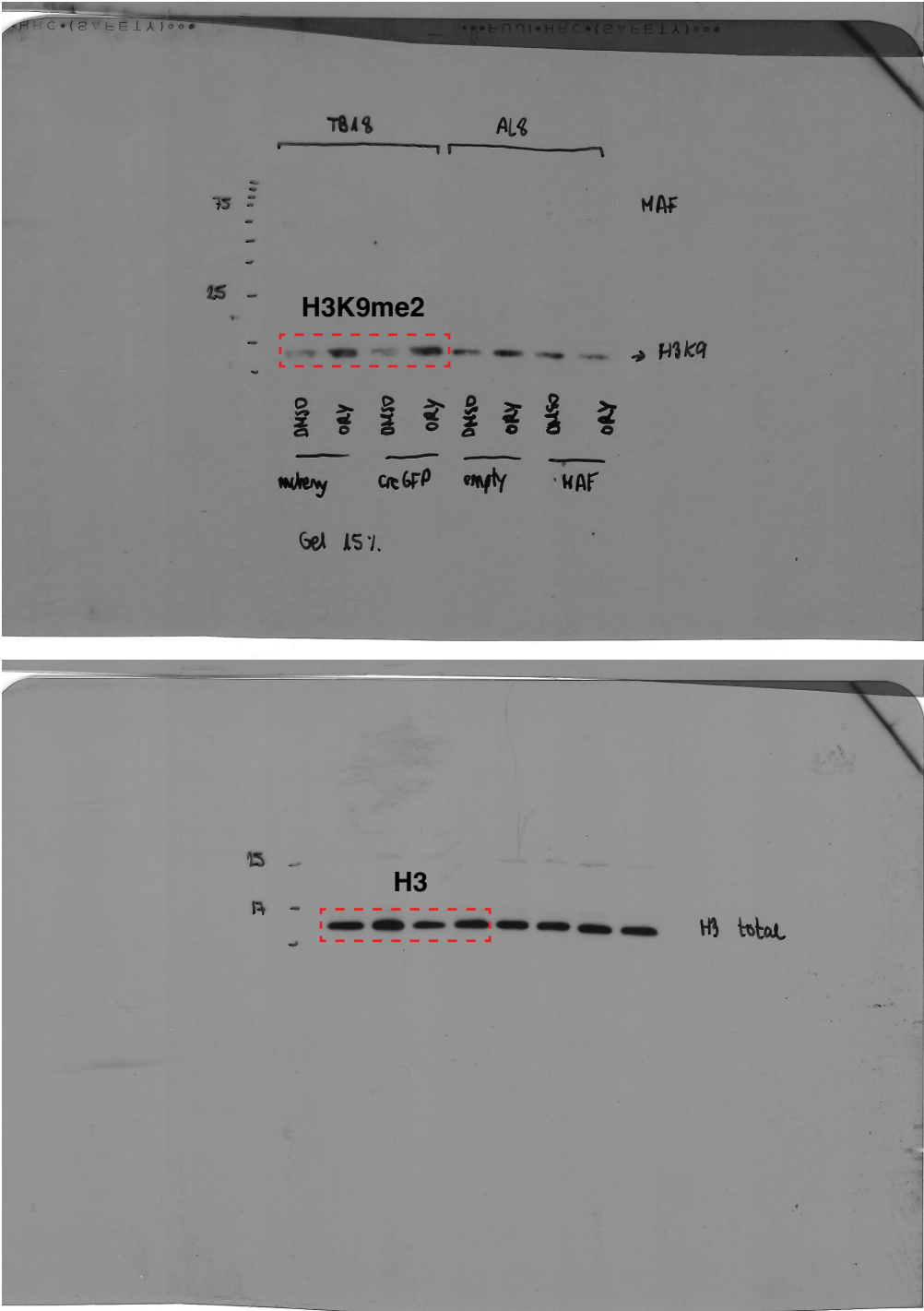

Supplement: Supplementary file 16 — Unprocessed western blots. [file 41556_2023_1281_MOESM16_ESM.pdf]

Extended Data Fig.1h

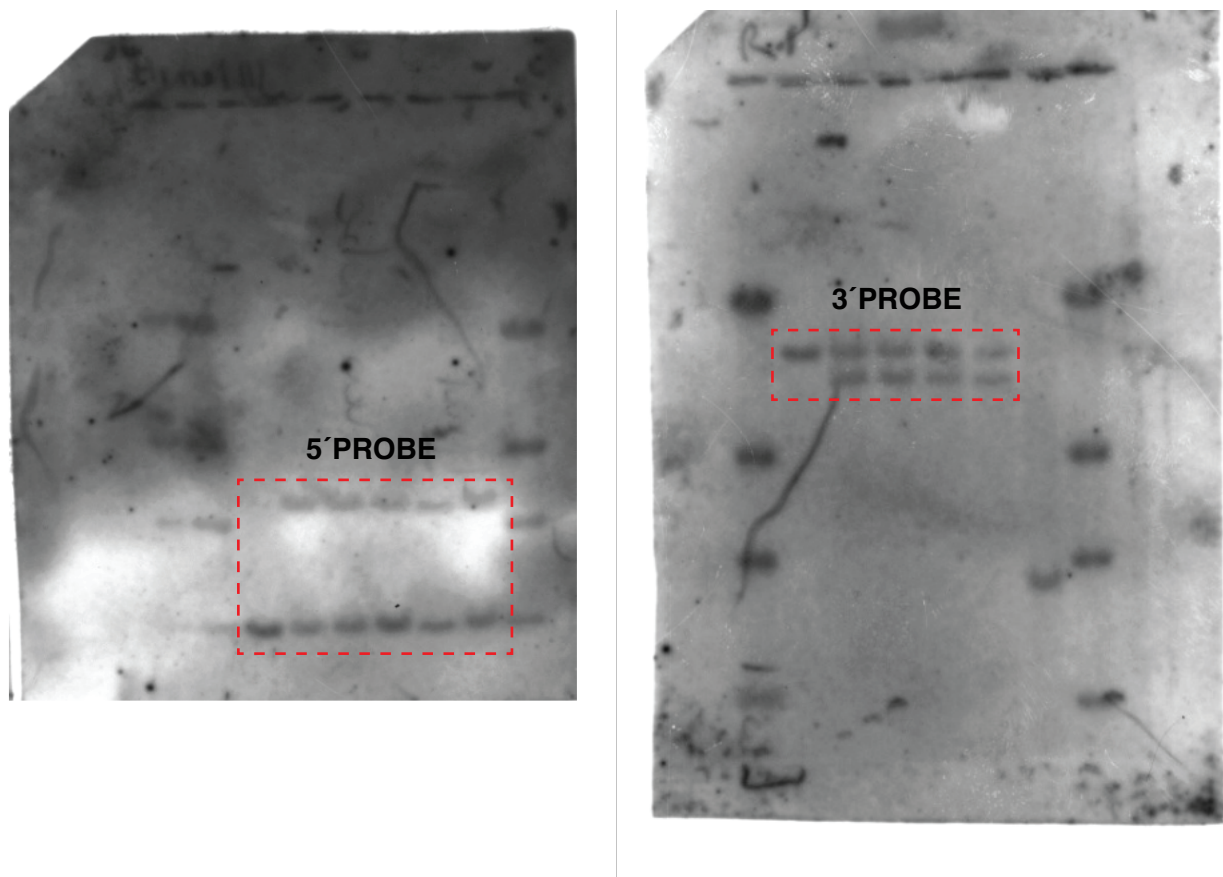

Extended Data Fig. 1i

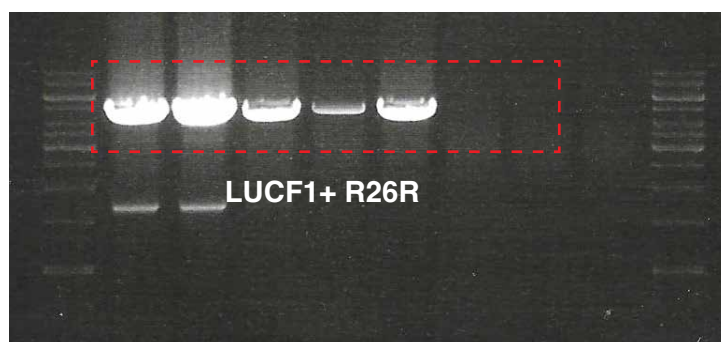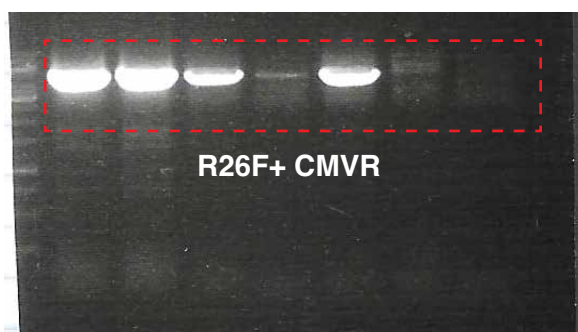

Supplement: Supplementary file 18 — Unprocessed western blots. [file 41556_2023_1281_MOESM18_ESM.pdf]

Extended Data Fig. 5g

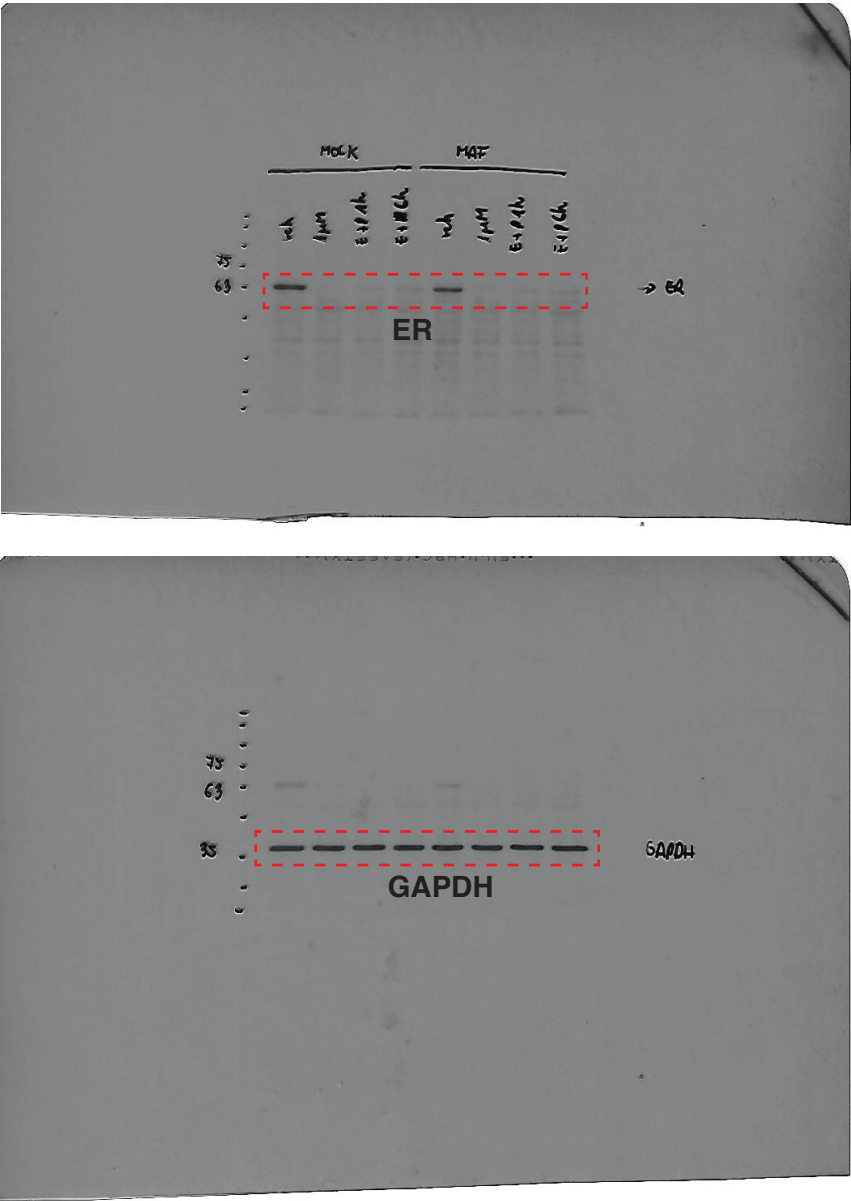

Supplement: Supplementary file 25 — Unprocessed western blots. [file 41556_2023_1281_MOESM25_ESM.pdf]

Extended Data Fig. 9c UP

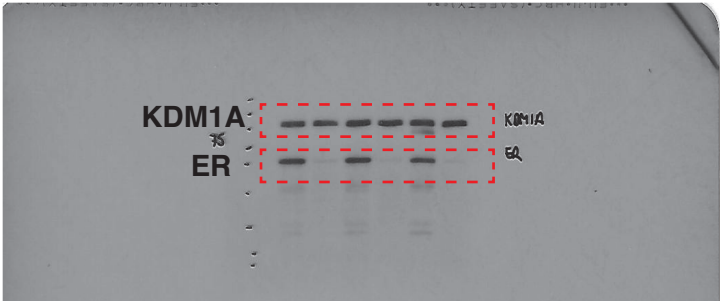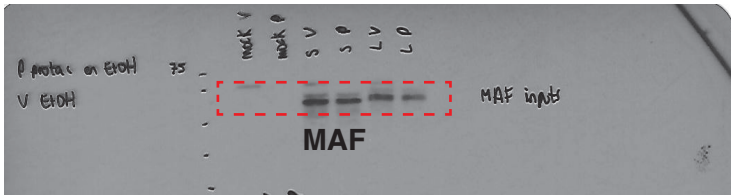

Blot development with LICOR

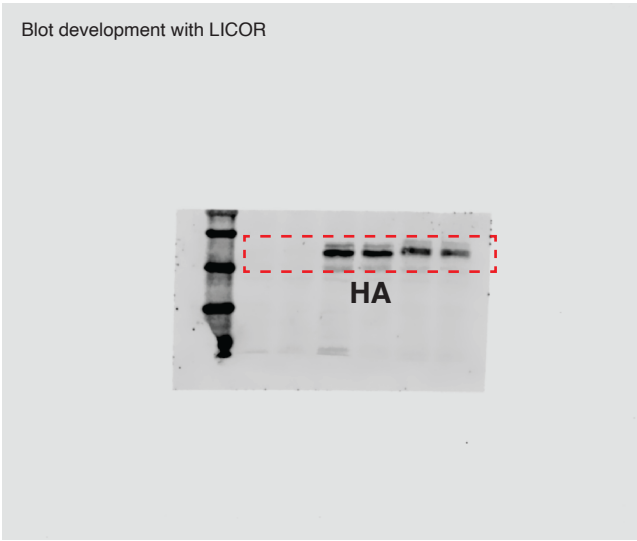

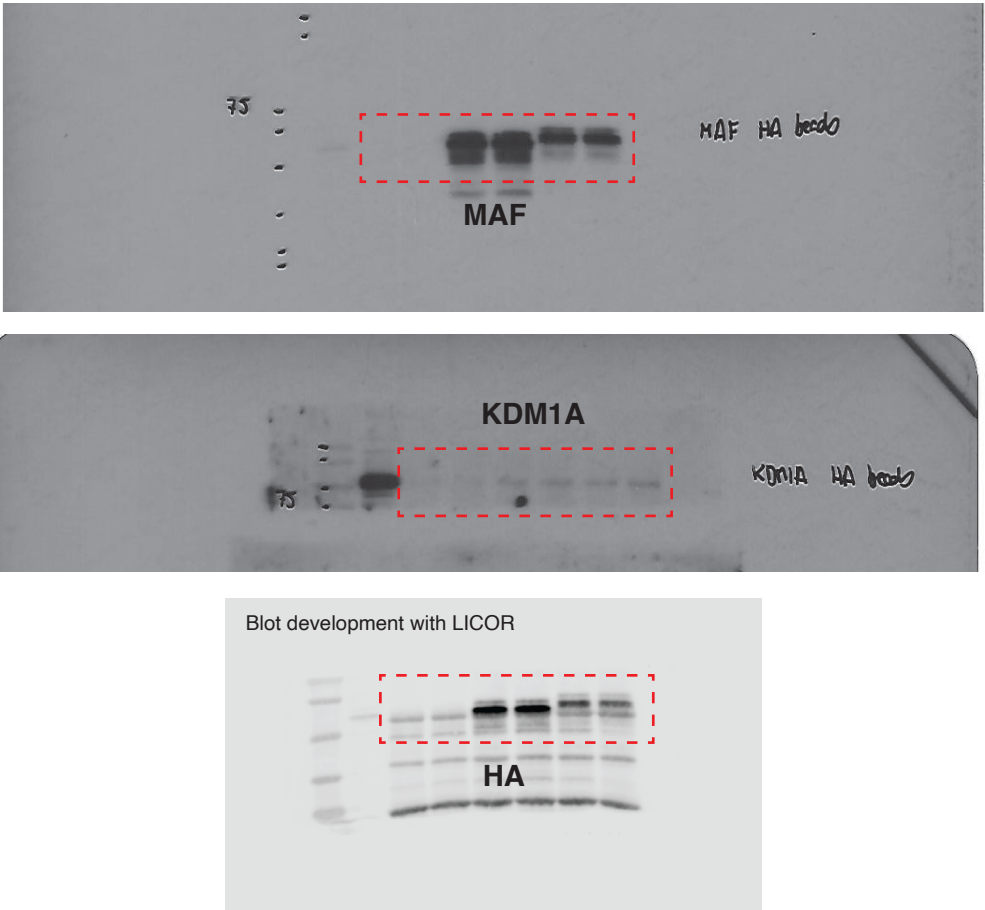

Extended Data Fig. 9f UP

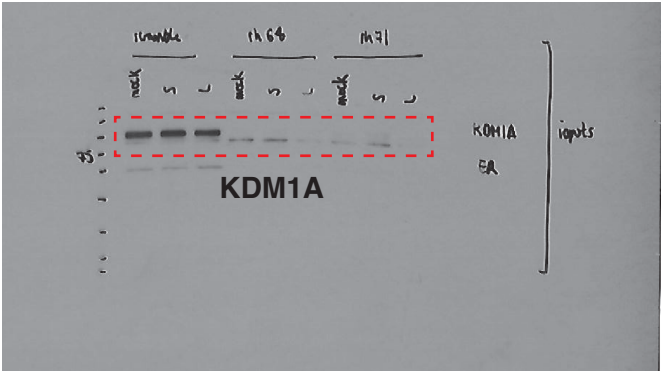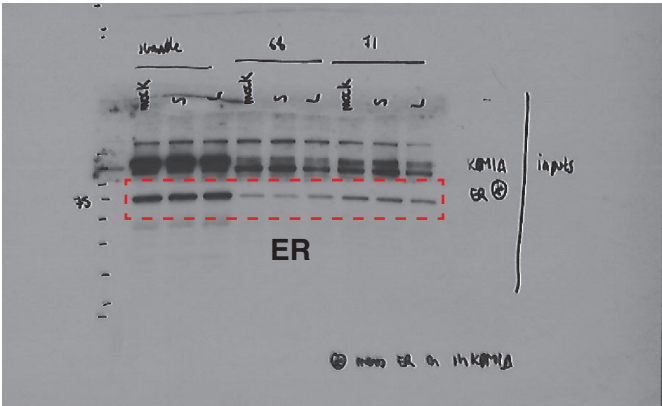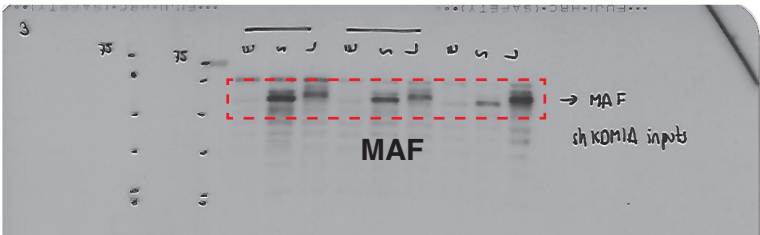

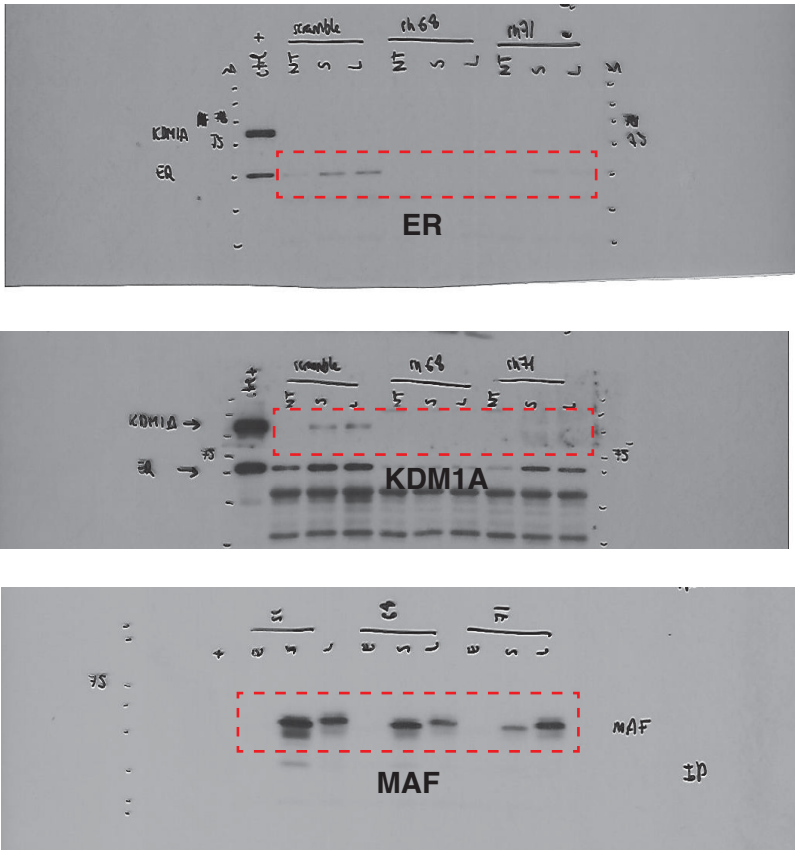

Supplement: Supplementary file 27 — Unprocessed western blots. [file 41556_2023_1281_MOESM27_ESM.pdf]

Extended Data Fig. 10e

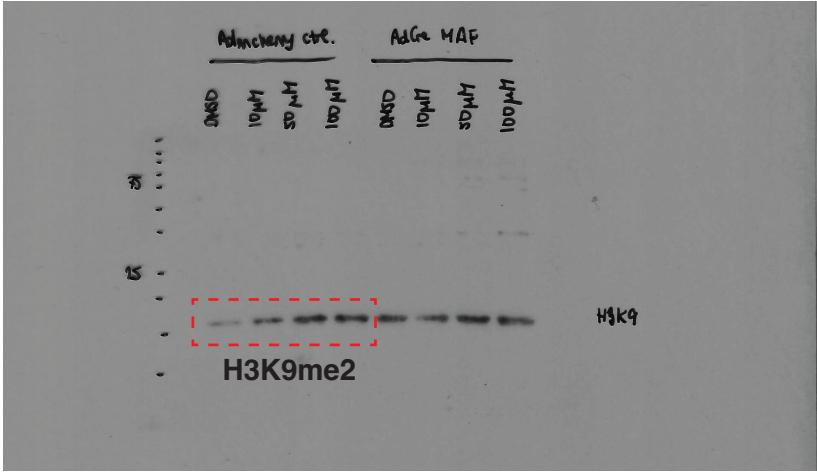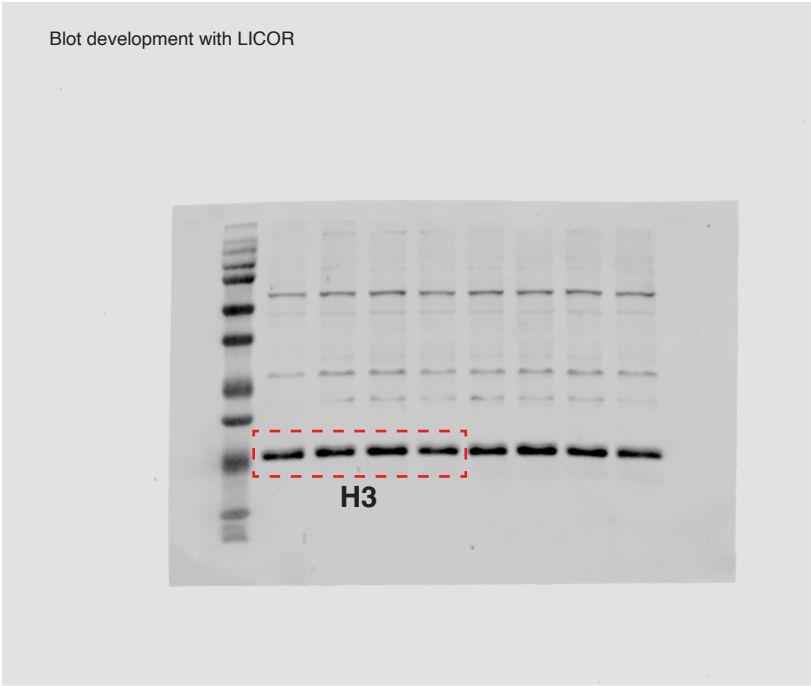

Supplement: Supplementary file 29 — Unprocessed western blots. [file 41556_2023_1281_MOESM29_ESM.pdf]
