# Supplementary material for: MAF amplification licenses ERα through epigenetic remodelling to drive breast cancer metastasis
Source: Nat Cell Biol. 2023 Nov 9;25(12):1833–47. doi: 10.1038/s41556-023-01281-y (PMC10709142; doi:10.1038/s41556-023-01281-y)

Uncropped Western blots related to Extended Data figures

Extended Data Fig.1h

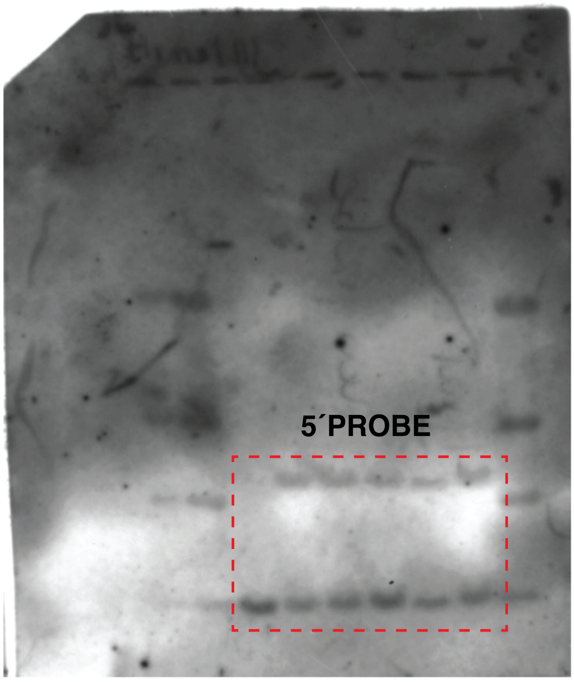

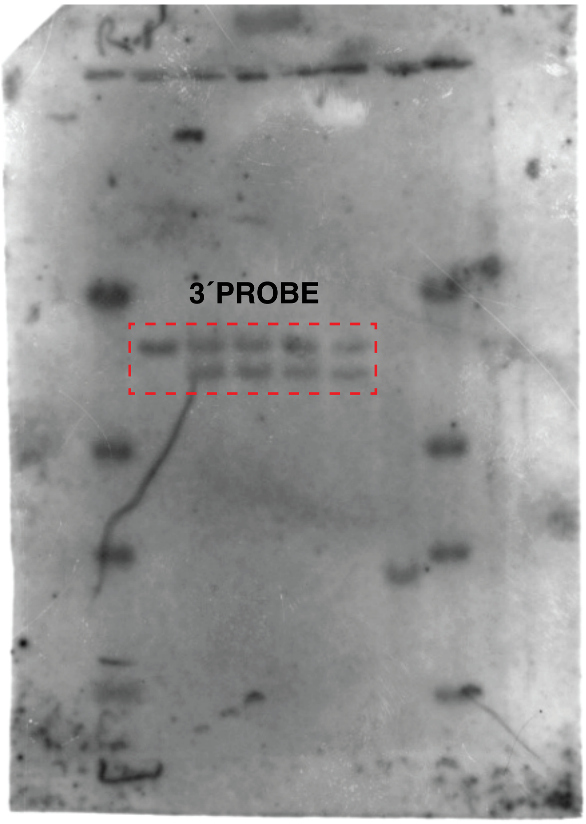

Extended Data Fig. 1i

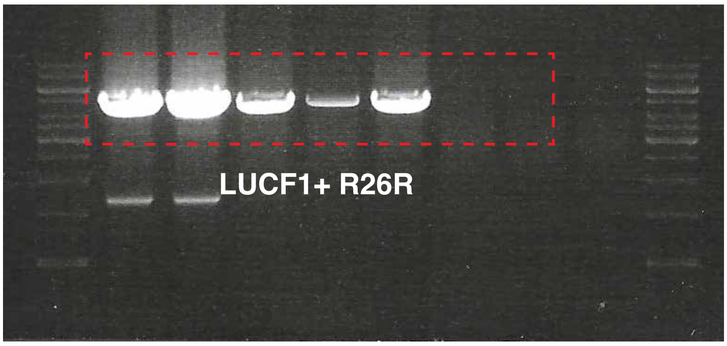

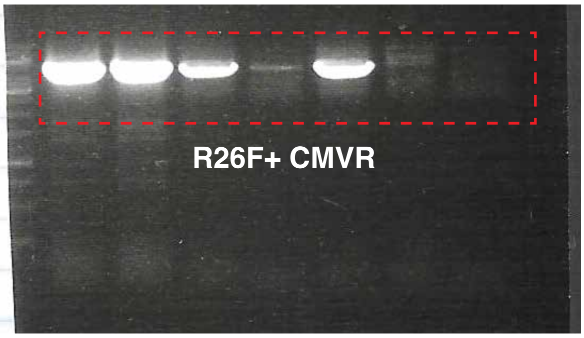

Extended Data Fig. 2e

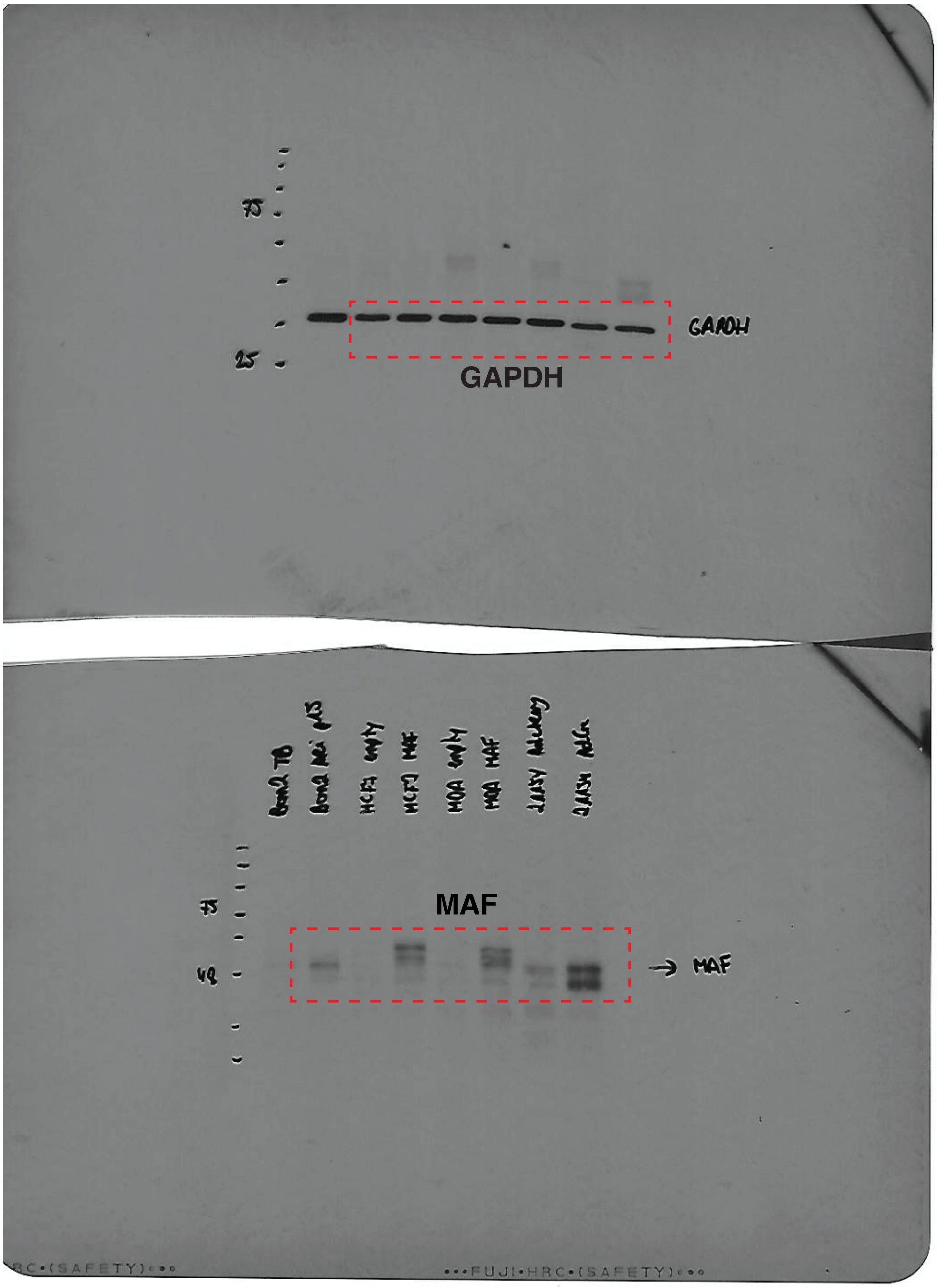

Extended Data Fig. 3a

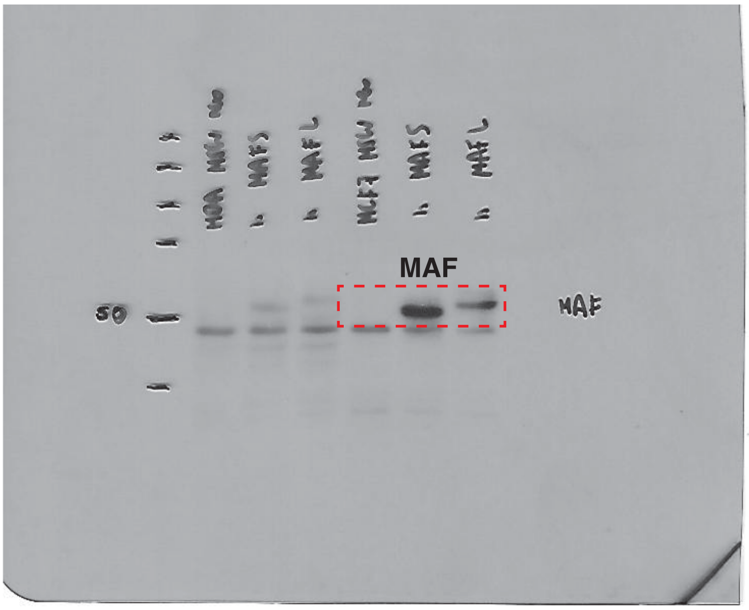

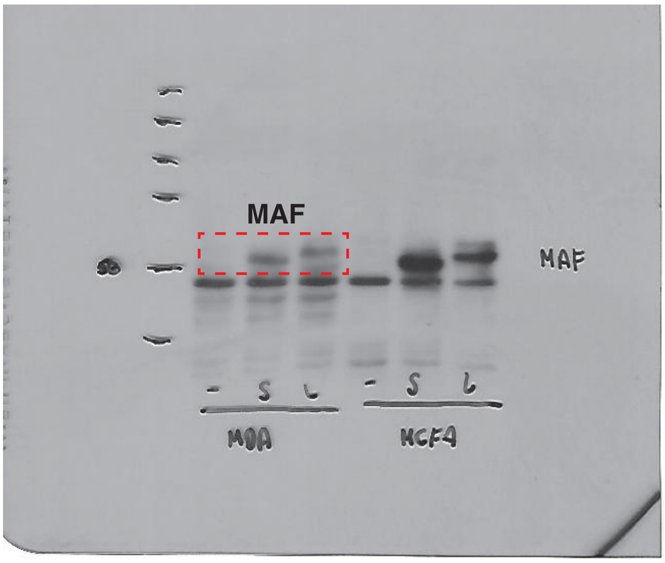

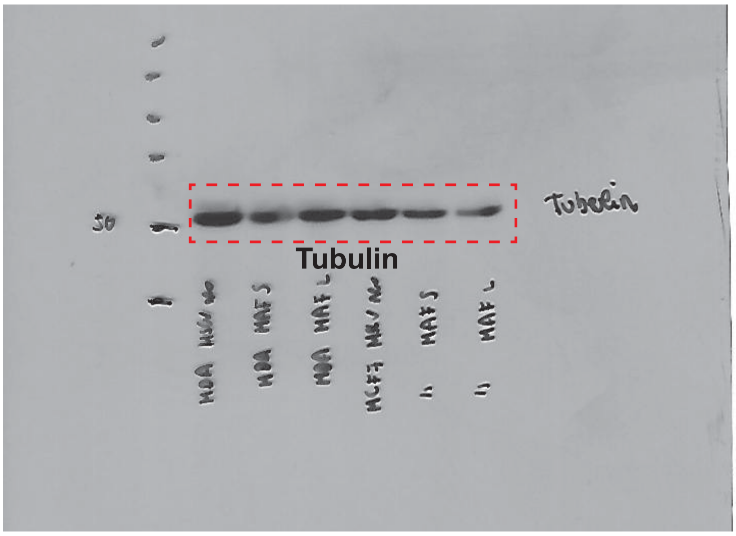

Extended Data Fig. 3b LEFT

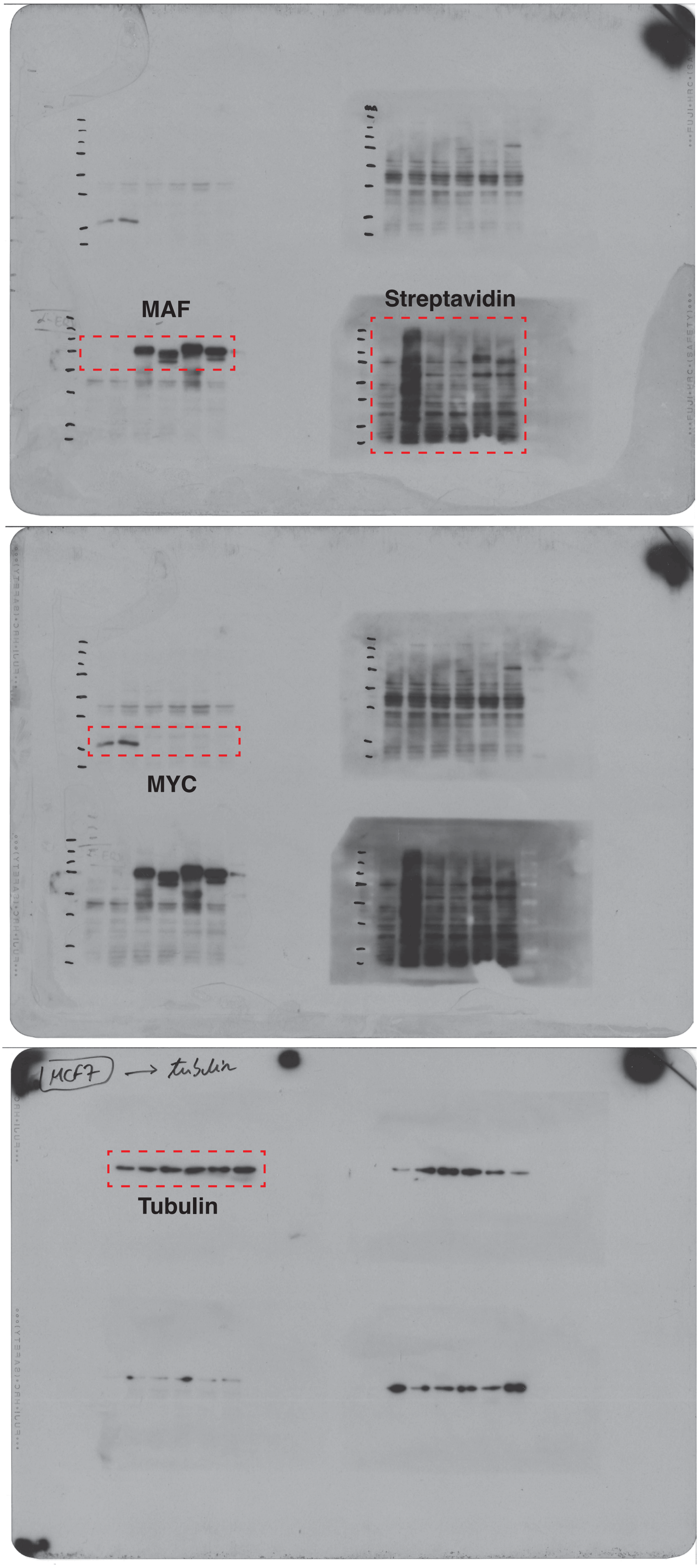

Extended Data Fig. 3b RIGHT

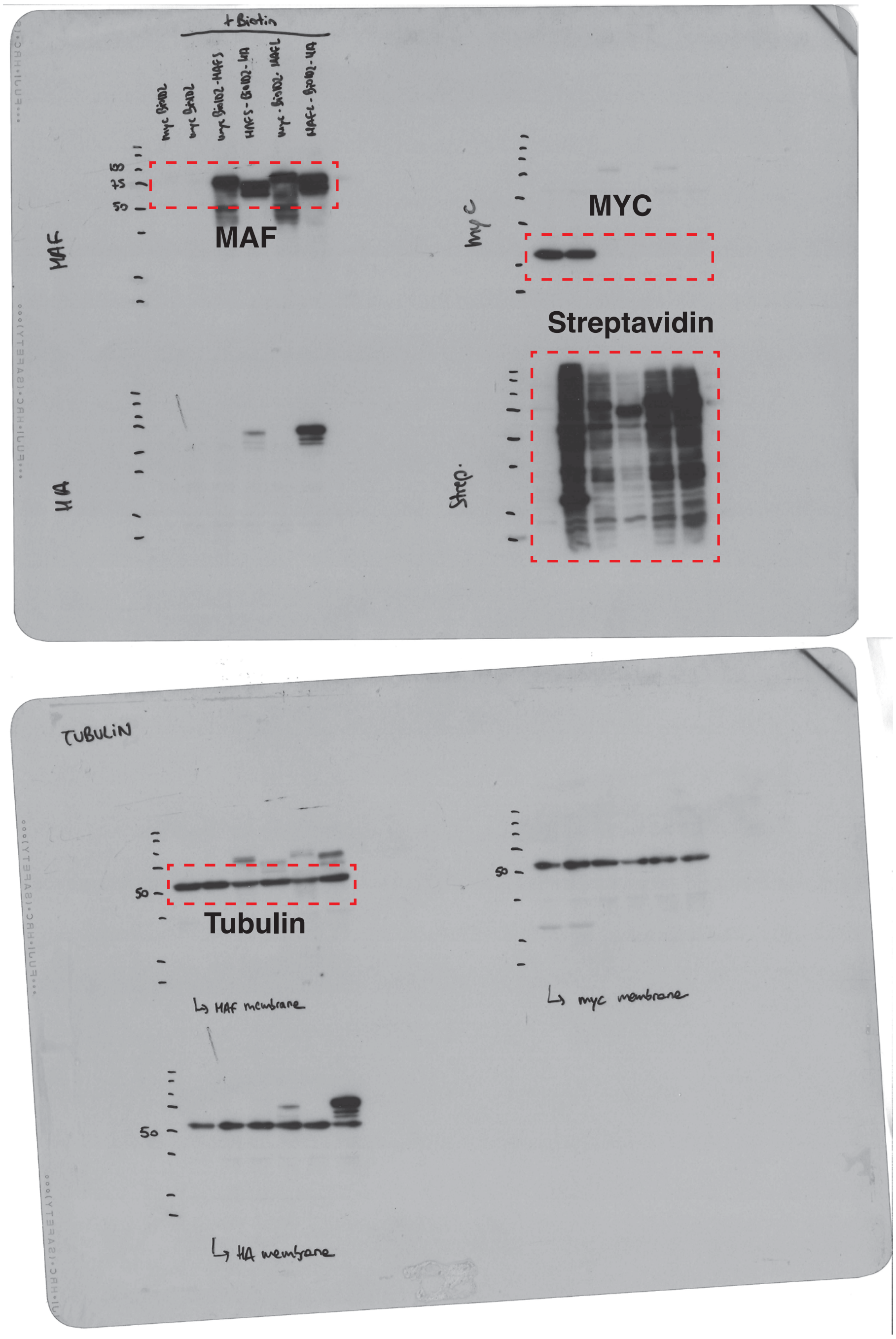

Extended Data Fig. 3d

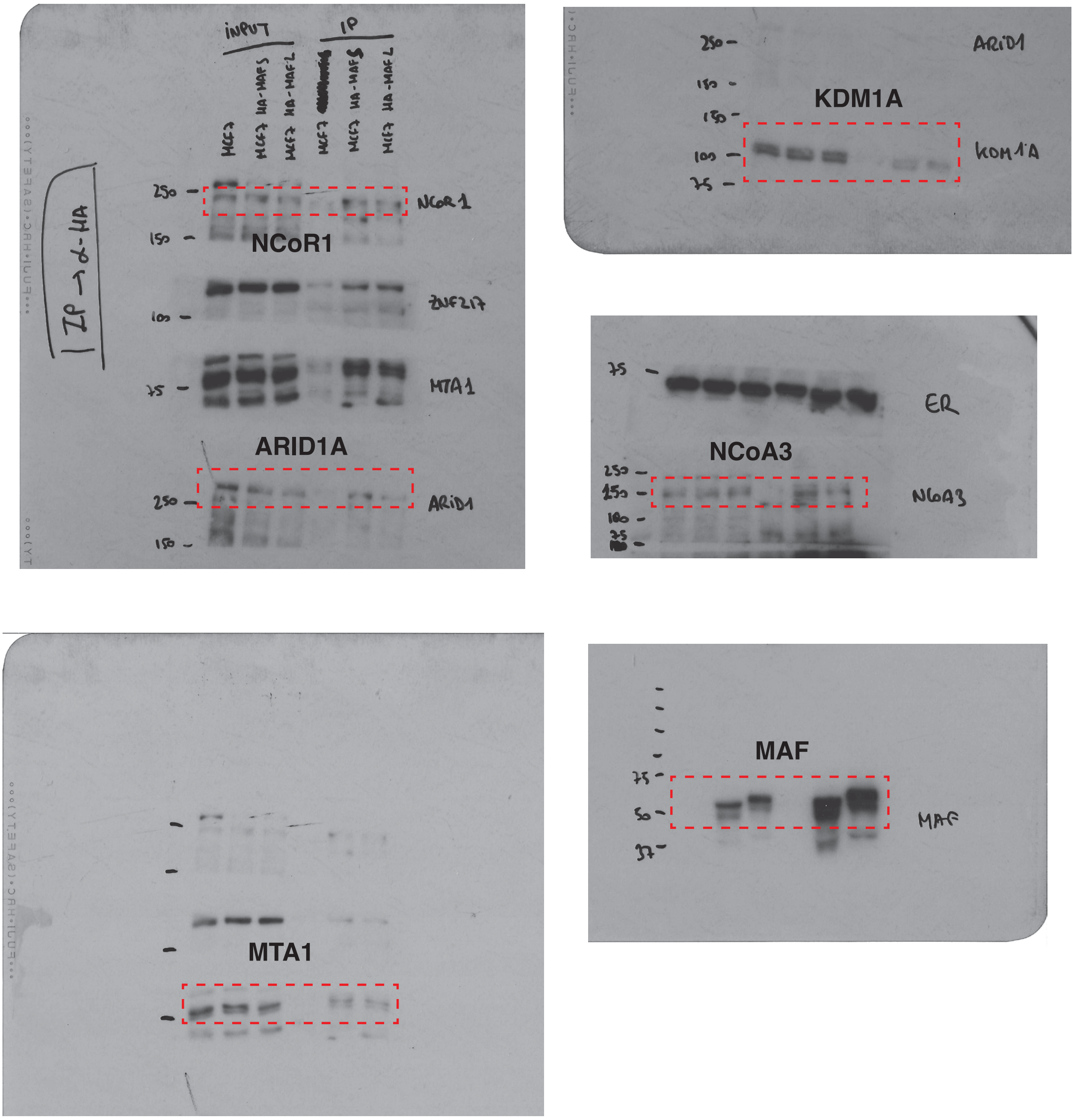

Extended Data Fig. 3g

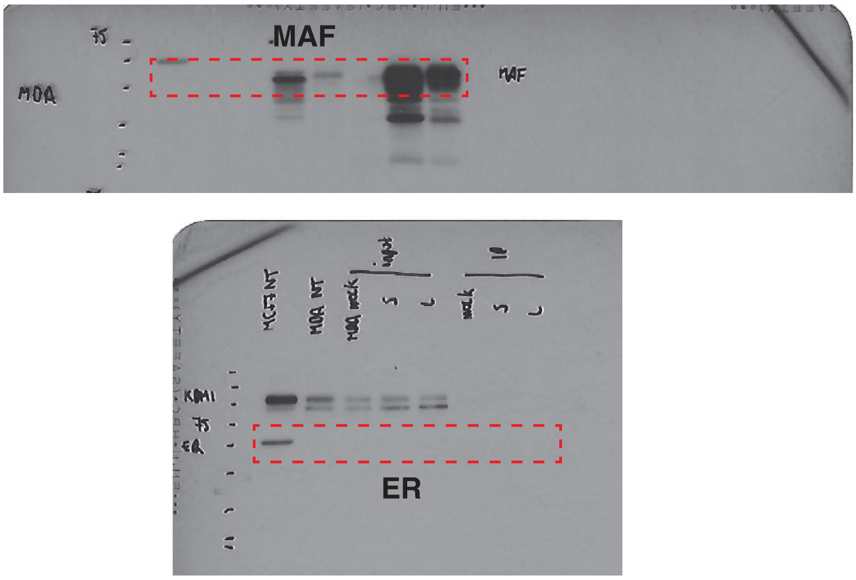

Extended Data Fig. 5g

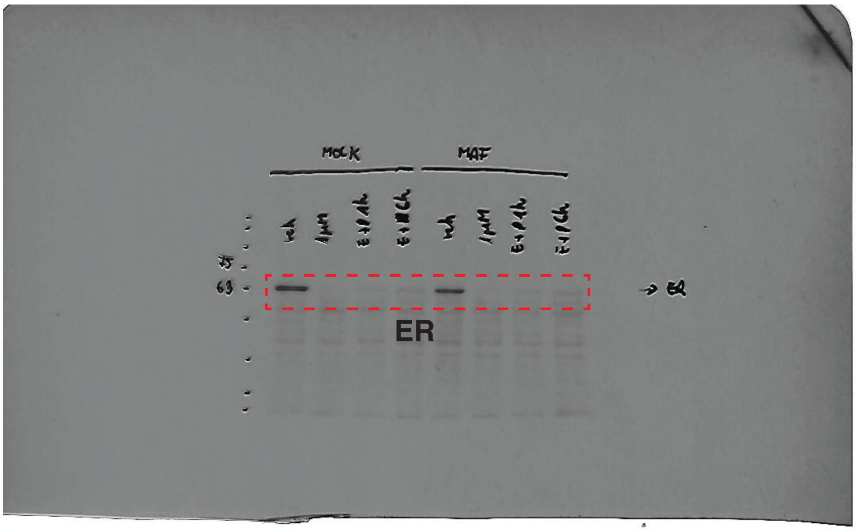

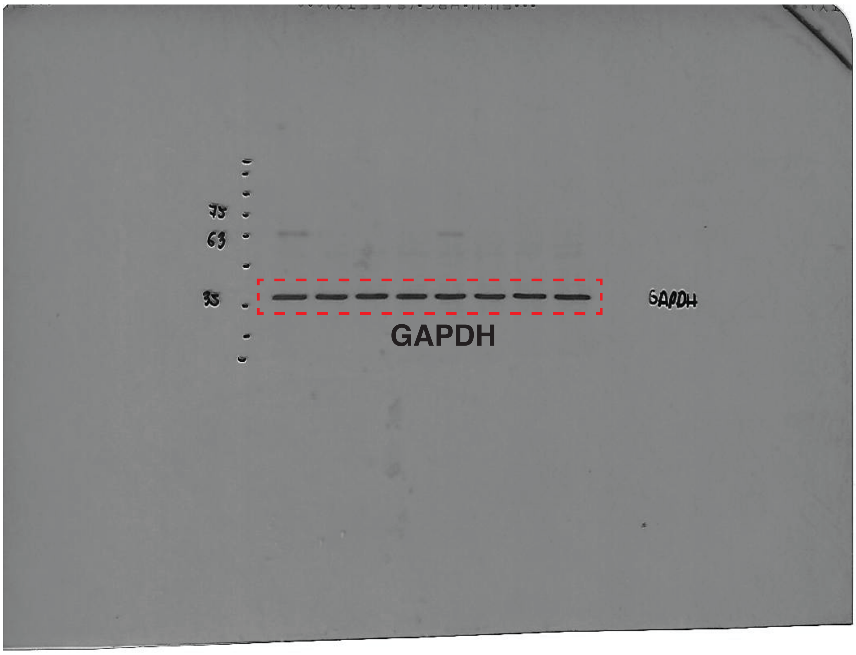

Extended Data Fig. 9c UP

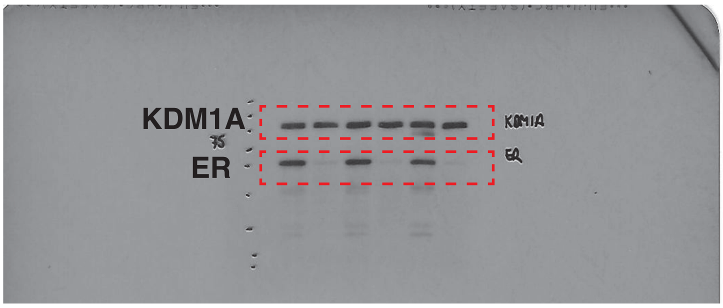

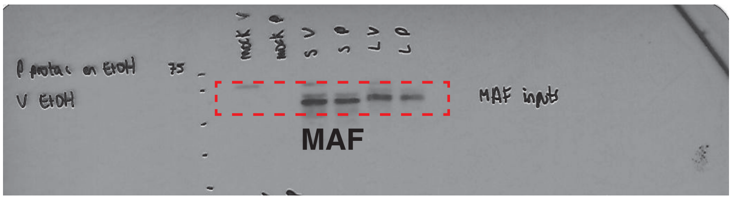

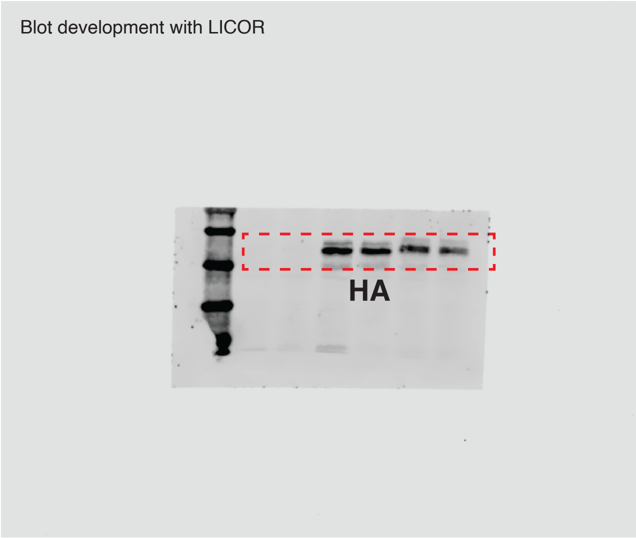

Extended Data Fig. 9c DOWN

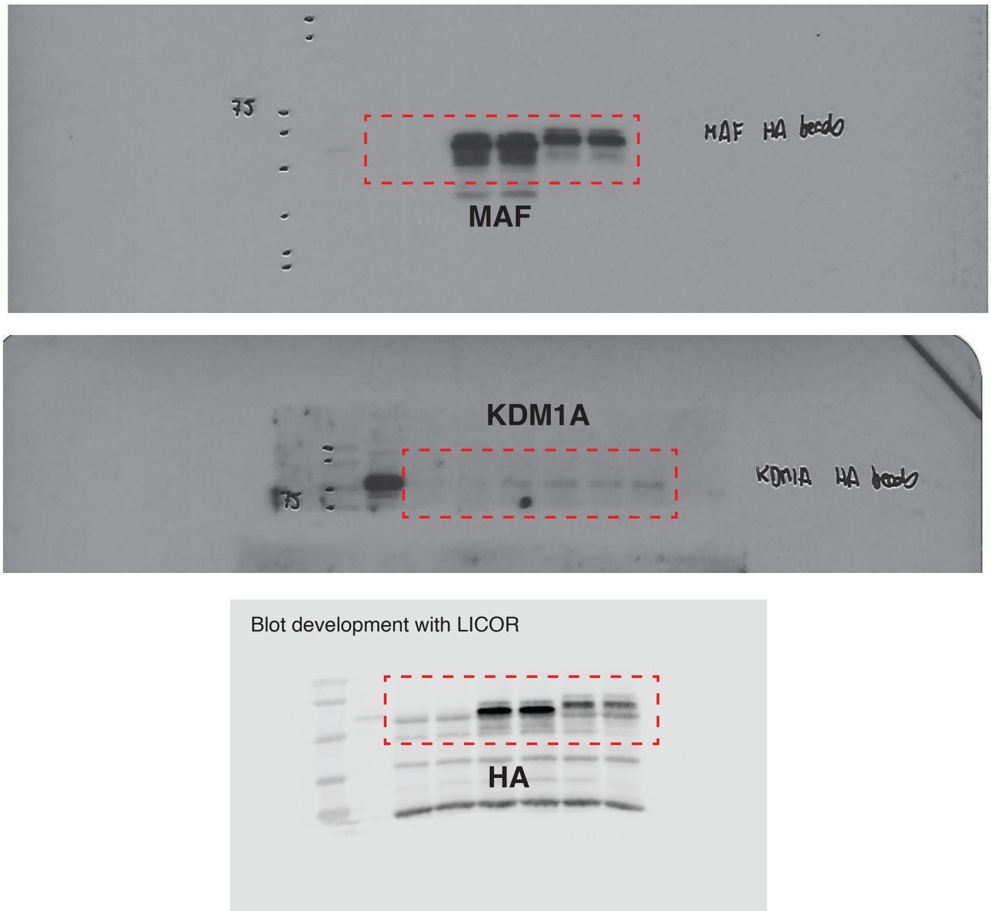

Extended Data Fig. 9f UP

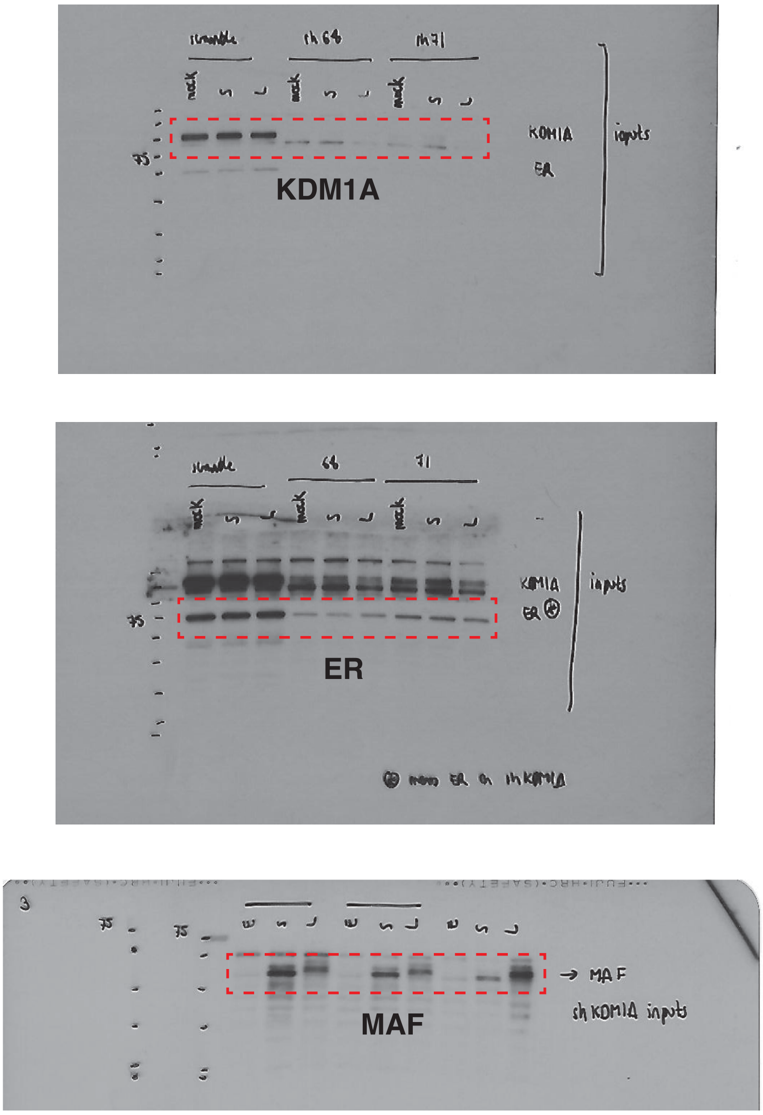

Extended Data Fig. 9f DOWN

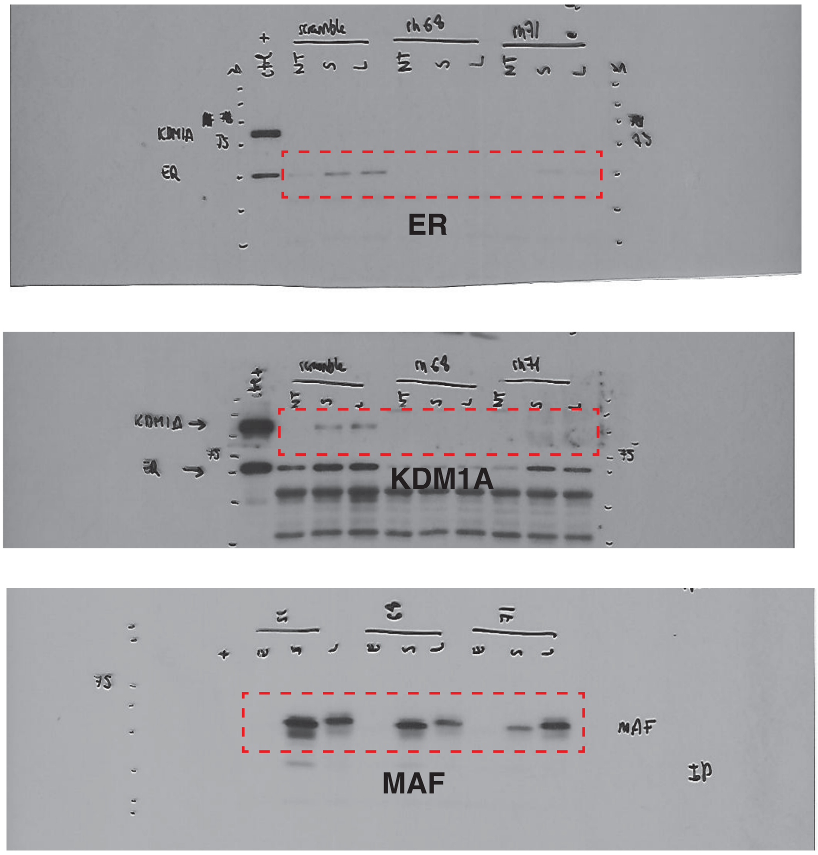

Extended Data Fig. 10e

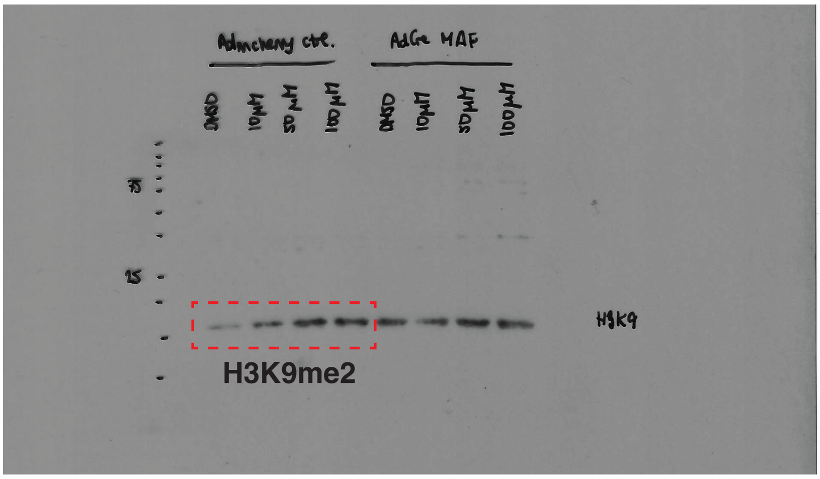

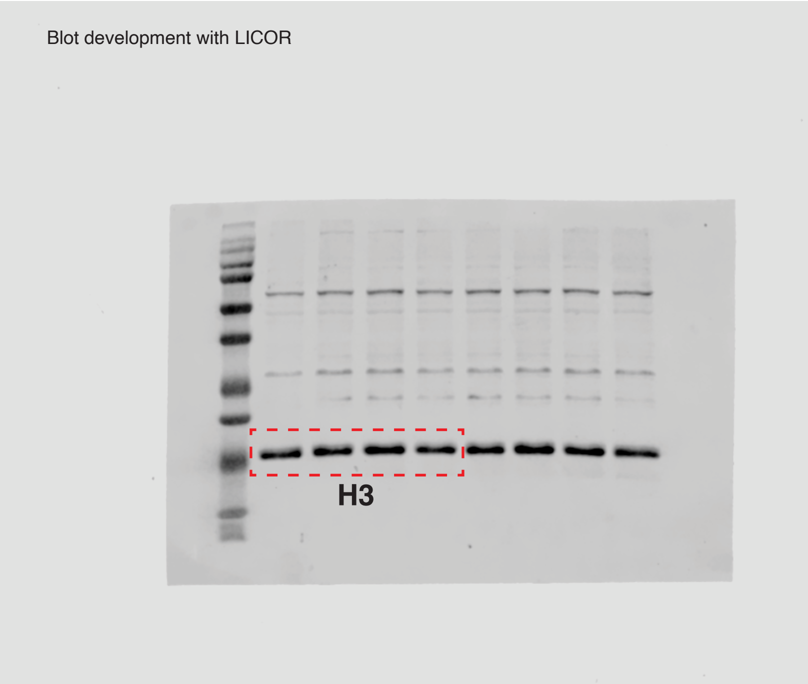

Supplement: Supplementary file 20 — Unprocessed western blots. [file 41556_2023_1281_MOESM20_ESM.pdf]
