# Supplementary material for: MAF amplification licenses ERα through epigenetic remodelling to drive breast cancer metastasis
Source: Nat Cell Biol. 2023 Nov 9;25(12):1833–47. doi: 10.1038/s41556-023-01281-y (PMC10709142; doi:10.1038/s41556-023-01281-y)

Extended Data Fig. 3a

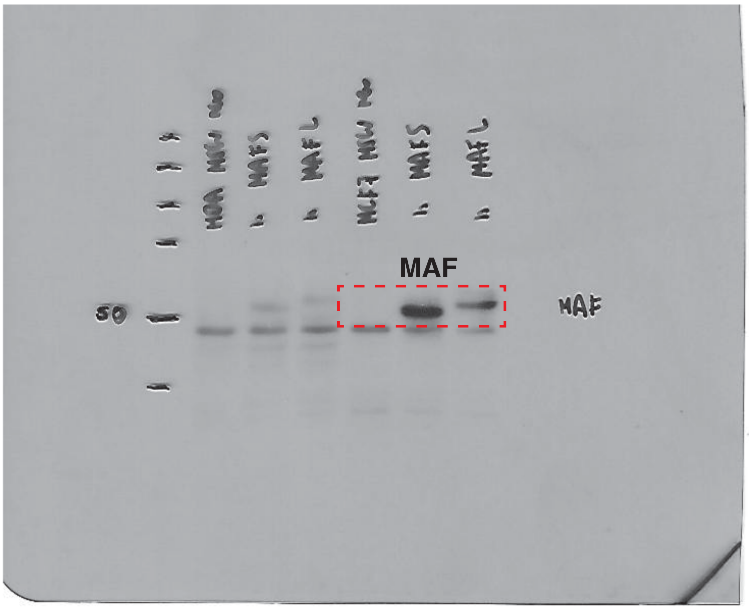

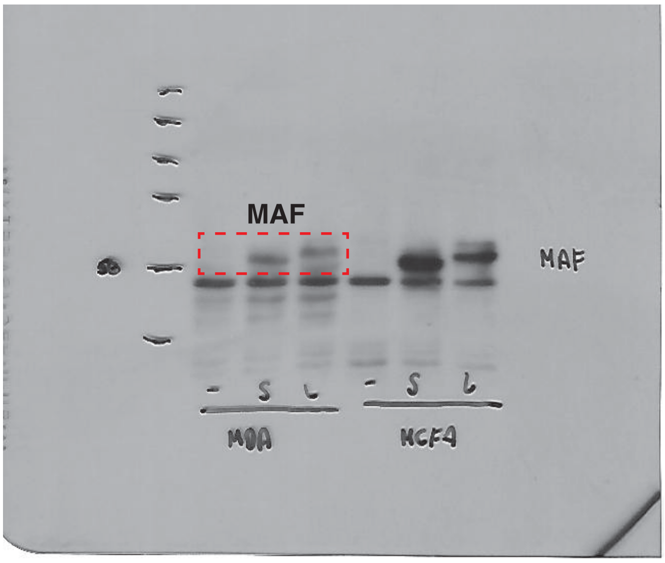

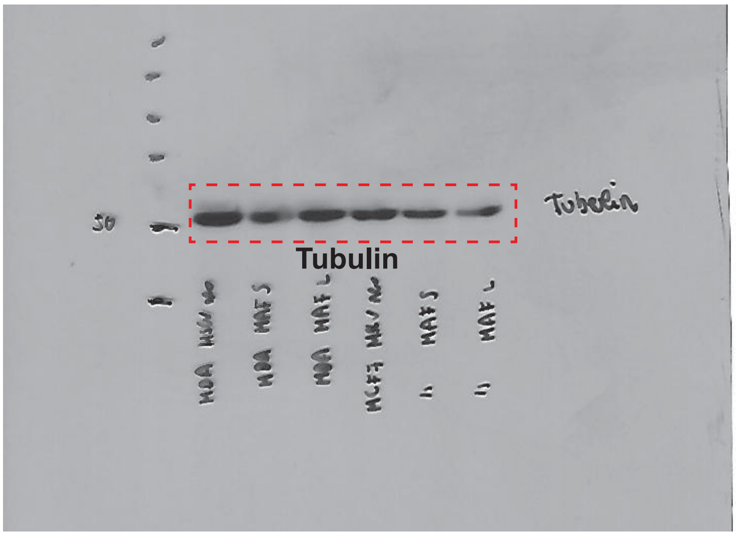

Extended Data Fig. 3b LEFT

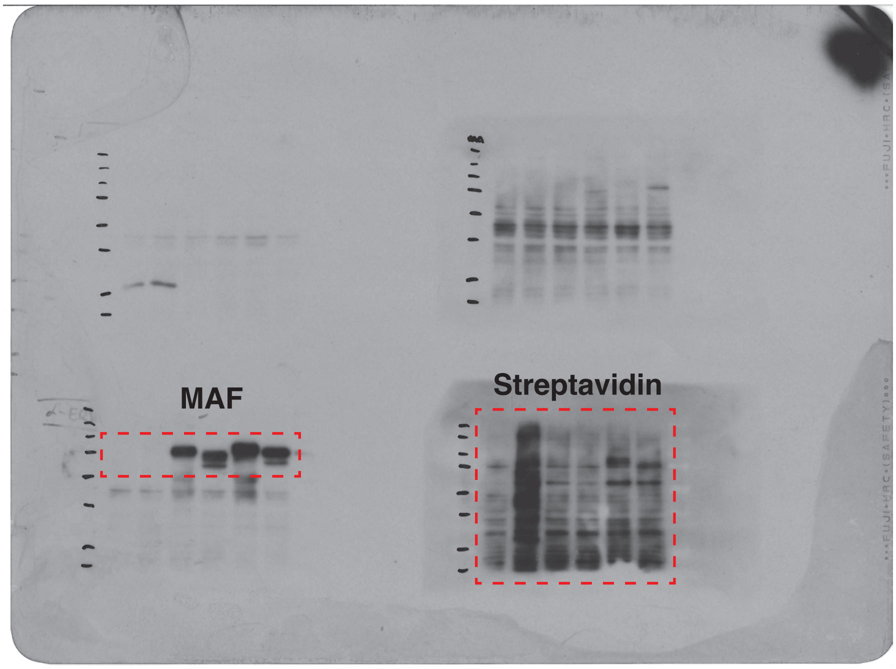

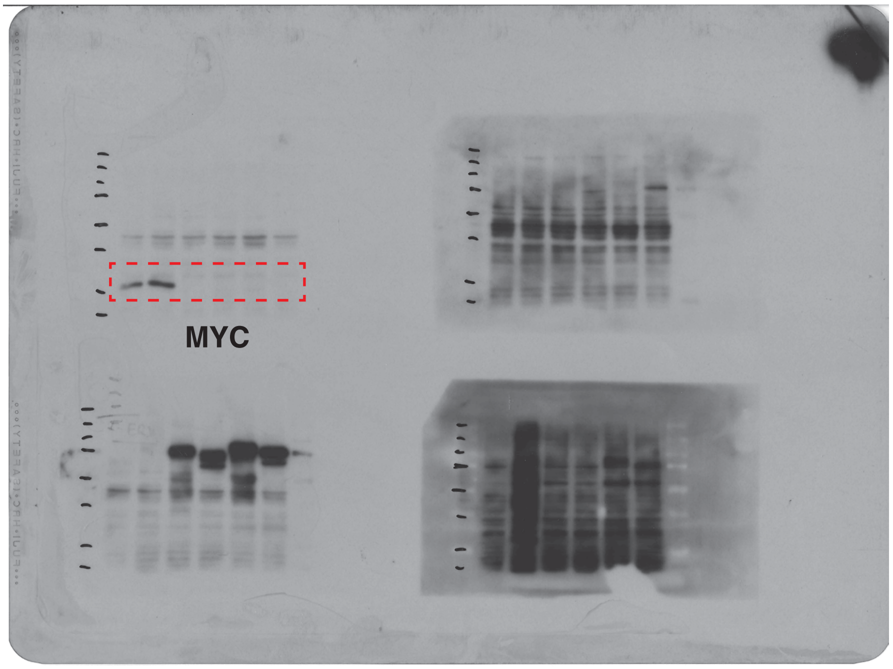

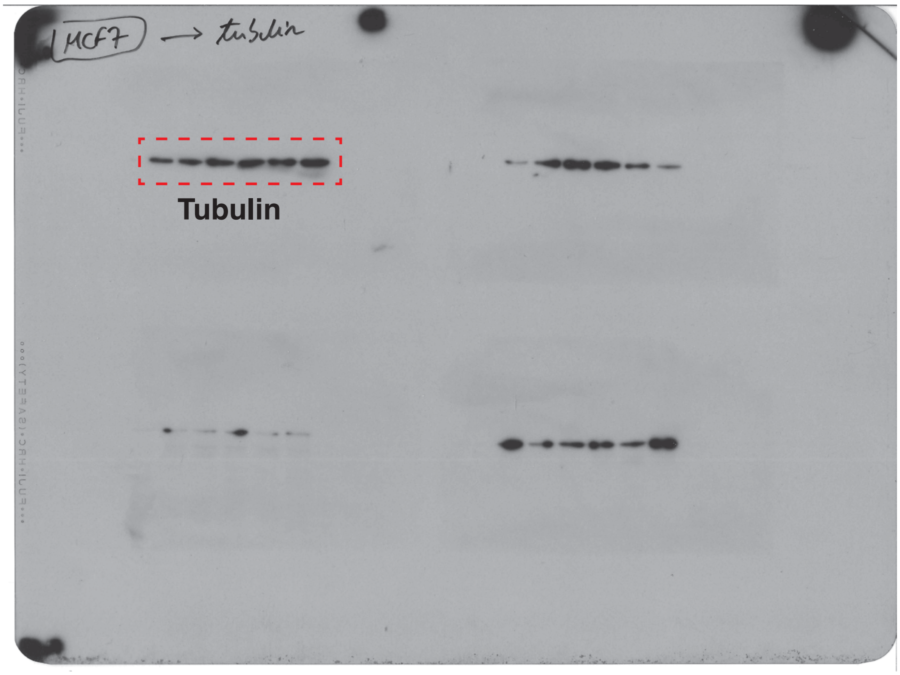

Extended Data Fig. 3b RIGHT

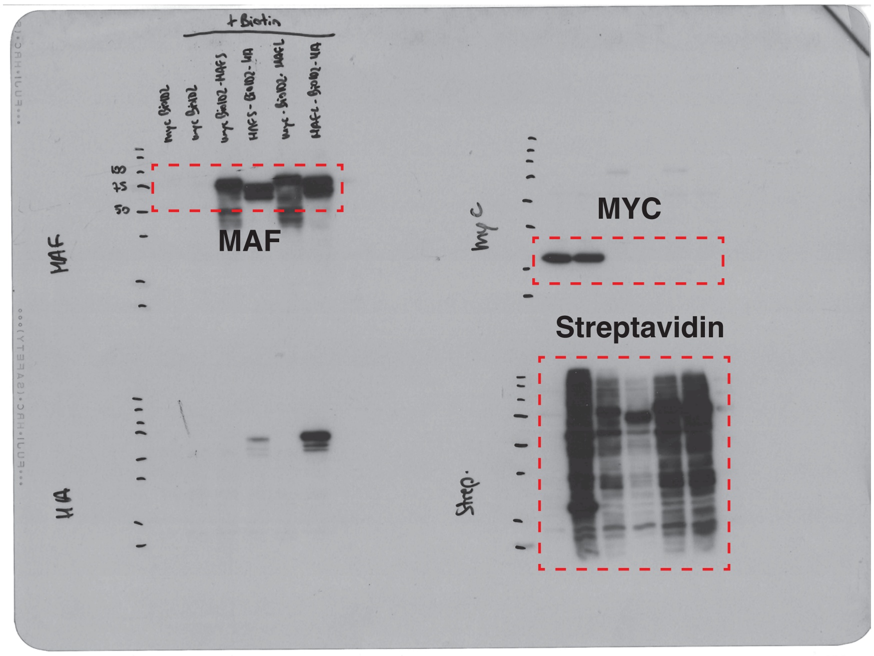

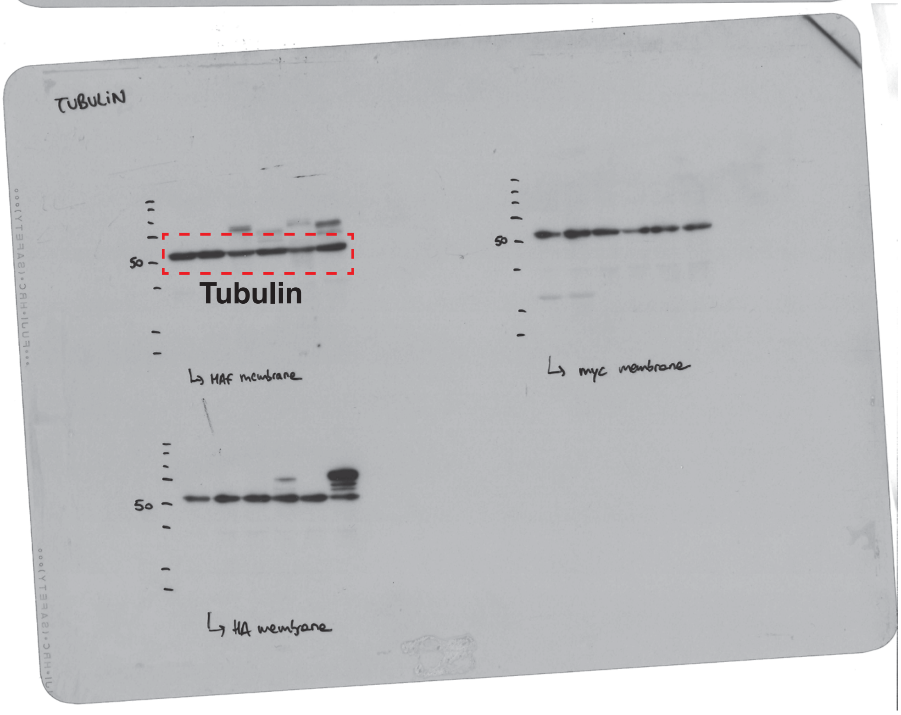

Extended Data Fig. 3d

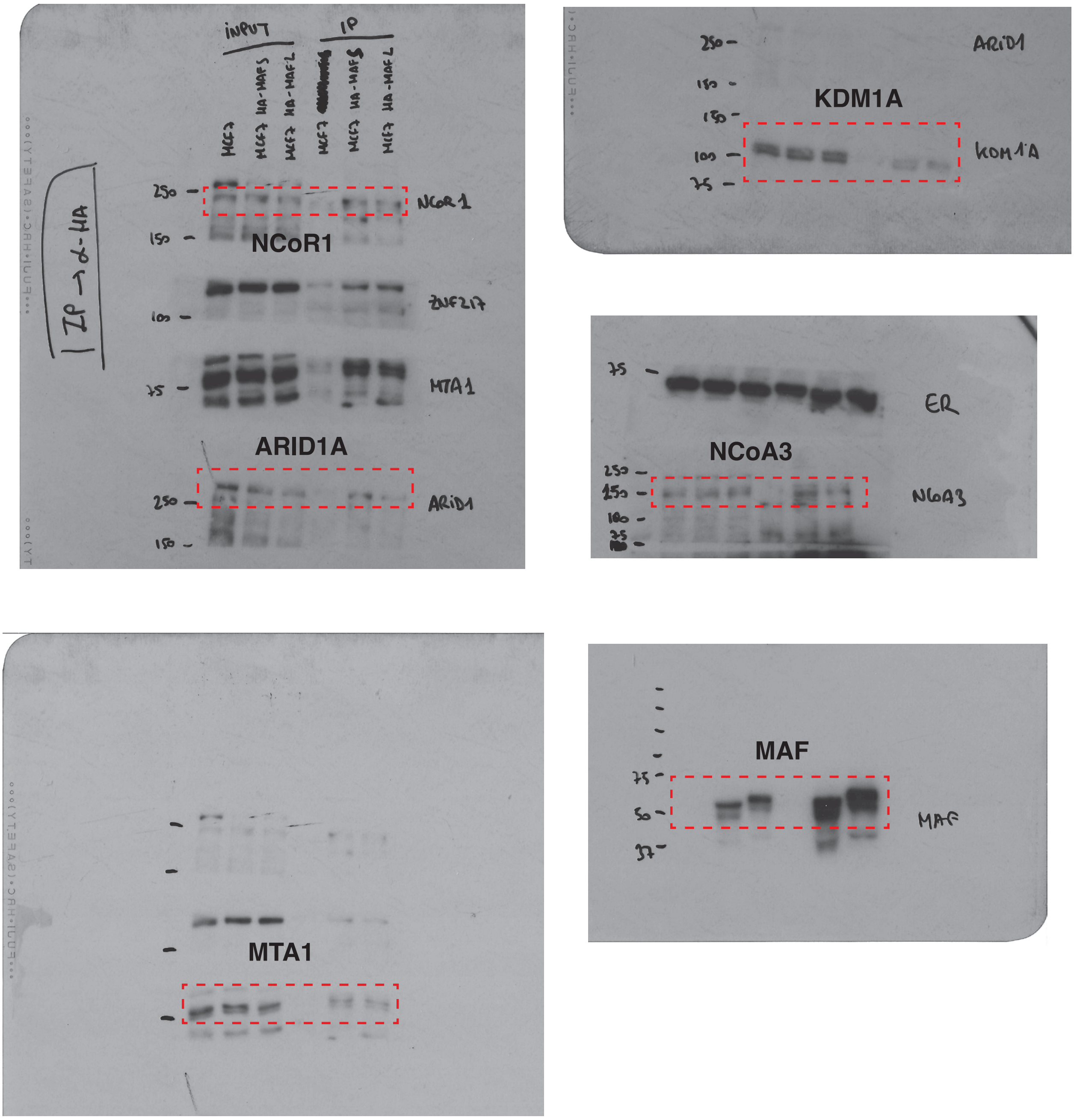

Extended Data Fig. 3g

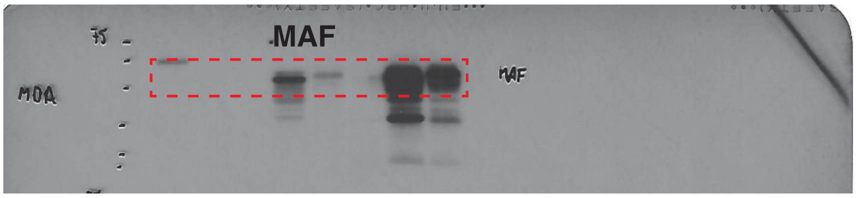

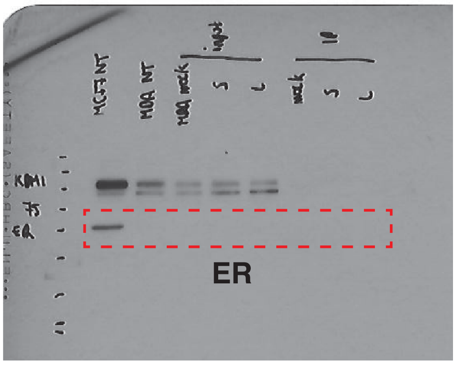

Supplement: Supplementary file 22 — Unprocessed western blots. [file 41556_2023_1281_MOESM22_ESM.pdf]
